# Effect of the combination of photobiomodulation therapy and the intralesional administration of corticoid in the preoperative and postoperative periods of keloid surgery: A randomized, controlled, double-blind trial protocol study

**DOI:** 10.1371/journal.pone.0263453

**Published:** 2022-02-15

**Authors:** Jefferson André Pires, Erick Frank Bragato, Marcos Momolli, Marina Bertoni Guerra, Leonel Manea Neves, Meire Augusto de Oliveira Bruscagnin, Anna Carolina Ratto Tempestini Horliana, Kristianne Porta Santos Fernandes, Sandra Kalil Bussadori, Raquel Agnelli Mesquita Ferrari

**Affiliations:** 1 Department of Biophotonics Applied to Health Sciences, Universidade Nove de Julho (UNINOVE), São Paulo, São Paulo, Brazil; 2 Department of Plastic Surgery, Mandaqui Hospital Complex, São Paulo, São Paulo, Brazil; 3 Department of Rehabilitation Science Applied to Health Sciences, Universidade Nove de Julho (UNINOVE), São Paulo, São Paulo, Brazil; University of Texas Medical Branch at Galveston, UNITED STATES

## Abstract

Keloid scars are characterized by the excessive proliferation of fibroblasts and an imbalance between the production and degradation of collagen, leading to its buildup in the dermis. There is no “gold standard” treatment for this condition, and the recurrence is frequent after surgical procedures removal. *In vitro* studies have demonstrated that photobiomodulation (PBM) using the blue wavelength reduces the proliferation speed and the number of fibroblasts as well as the expression of TGF-β. There are no protocols studied and established for the treatment of keloids with blue LED. Therefore, the purpose of this study is to determine the effects of the combination of PBM with blue light and the intralesional administration of the corticoid triamcinolone hexacetonide on the quality of the remaining scar by Vancouver Scar Scale in the postoperative period of keloid surgery. A randomized, controlled, double-blind, clinical trial will be conducted involving two groups: 1) Sham (n = 29): intralesional administration of corticoid (IAC) and sham PBM in the preoperative and postoperative periods of keloid removal surgery; and 2) active PBM combined with IAC (n = 29) in the preoperative and postoperative periods of keloid removal surgery. Transcutaneous PBM will be performed on the keloid region in the preoperative period and on the remaining scar in the postoperative period using blue LED (470 nm, 400 mW, 4J per point on 10 linear points). The patients will answer two questionnaires: one for the assessment of quality of life (Qualifibro-UNIFESP) and one for the assessment of satisfaction with the scar (PSAQ). The team of five plastic surgeons will answer the Vancouver Scar Scale (VSS). All questionnaires will be administered one, three, six, and twelve months postoperatively. The keloids will be molded in silicone prior to the onset of treatment and prior to excision to assess pre-treatment and post-treatment size. The same will be performed for the remaining scar at one, three, six, and twelve months postoperatively. The removed keloid will be submitted to histopathological analysis for the determination of the quantity of fibroblasts, the organization and distribution of collagen (picrosirius staining), and the genic expression of TGF-β (qPCR). All data will be submitted to statistical analysis.

**Trial registration:** This study is registered in ClinicalTrials.gov (ID: NCT04824612).

## Introduction

A keloid is a raised scar characterized by the excessive proliferation of fibroblasts and an imbalance between the production and degradation of collagen, leading to its buildup in the dermis. Keloids do not regress spontaneously and surpass the line of the scar with disorganized, uneven growth [1–5]. Besides the unpleasant esthetic aspect, keloids are often painful and pruritic and can lead to functional disability when occurring on joints. This combination of factors exerts a negative impact on psychosocial aspects and quality of life [6, 7]. Keloids do not regress spontaneously and surpass the line of the scar with disorganized, uneven growth [1–5]. Besides the unpleasant esthetic aspect, keloids are often painful and pruritic and can lead to functional disability when occurring on joints. This combination of factors exerts a negative impact on psychosocial aspects and quality of life [6, 7].

The genesis of this condition is not yet fully understood due mainly to the lack of *in vivo* studies and the fact that keloids are not described in animals, which hinders the adequate biological study of this entity [1, 2, 7, 8]. However, studies have demonstrated that the inflammatory aspects [9] and hyperproliferation of fibroblasts is related to an increase in the expression of transforming growth factors beta 1 (TGF-β1) in endothelial cells during the process of neovascularization, which already have a high expression of vascular endothelial growth factor (VEGF) as well as an increase in the expression of connective tissue growth factor (CTGF) [4, 7, 10–14].

TGF-β plays a key role in the regulating the proliferation of fibroblasts and the synthesis of collagen. In the normal healing process, TGF-β levels diminish at the end of the tissue repair process. However, this does not occur in the case of keloids, where TGF-β remains high and dysregulated [2, 15, 16].

The literature reports several treatments for keloids, but many studies employ questionable methods and report imprecise results, which hinders the establishment of new treatment protocols. Thus, there is no consensus on the best therapy to minimize the risk of recurrence with minimal undesirable side effects [7, 10, 11, 17–29].

The intralesional administration of the corticoid triamcinolone hexacetonide (Triancil®, Apsen Farmacêutica S.A.) is considered the first line of treatment for keloids, with an established dose of 2.5 to 20 mg for facial skin and 20 to 40 mg for other regions of the body. Its mechanism is based on the reduction in the synthesis of collagen and glycosaminoglycans as well as the inhibition of fibroblast production [30]. Due to its anti-inflammatory and vasoconstrictive effects, reductions in pruritus and pain also occur. Studies report a variable recurrence rate of up to 50% [7, 18, 31–33].

There is no consensus on the quantity or time of treatment for the triamcinolone hexacetonide corticoid. Prolonged use can lead to ulcers, changes in the color of the skin, and the emergence of telangiectasias [7]. There are also reports of the reappearance of Cushing syndrome after its use [18, 30–33]. Besides these side effects, the isolated use of the corticoid often does not lead to the complete regression of the keloid and can result in unappealing residual scars, with a color change, broadening of the scar, telangiectasias, and depressions [18, 33].

Combined therapy involving surgical excision and the use of the injectable corticoid has demonstrated to be safety, with a little reduction in the recurrence rate by about 29% [34–39]. However, the considerable variation in the protocols and results hinders a precise evaluation.

Due to the high recurrence rate with the use of current treatments, a keloid can remain for a long time, causing a reduction in one’s quality of life [6]. This situation has led to the search for novel, minimally invasive products and technologies that can offer better treatment with fewer side effects and a lower recurrence rate.

Considering this context and these characteristics, the benefits of photobiomodulation (PBM) have been listed in the literature. The PBM consists of the use of low-intensity light sources (normally less than 500 mW) with non-ionizing irradiation in both the visible band (400–760 nm) and infrared band (760–1000 nm) of the electromagnetic spectrum, producing a positive biological effect on cells [40, 41].

Studies have reported the benefits of PBM on the healing process of postoperative scars from different types of surgery, demonstrating the safety and effectiveness of this form of therapy [42–51]. *In vitro* experimental studies using fibroblasts from keloids and human dermis have also demonstrated positive effects, such as an increase in the apoptosis rate and reduction in the cell division rate of these fibroblasts as well as reductions in the synthesis of collagen and the expression of TFG-β [10, 52–59].

Blue light (wavelength ranging from 410 to 480 nm), in particular, is reported to have in inhibitory effect on fibroblasts and TGF-β when administered with a higher energy density (up to 640 J/cm^2^) [10, 52–59].

It is possible that blue light interacts with mitochondrial chromophores in the same way as red and infrared light, as heme centers in cytochromes have significant peak absorption that coincides with the Soret band of porphyrins [60]. In the blue light spectrum, porphyrins and flavoproteins, such as NADH-dehydrogenase and succinate dehydrogenase, can function as photoreceptors [61].

An important aspect to emphasize is the low cost of the LED, as many studies show that in photobiomodulation it has the same effect as lasers [62].

Studies reporting the beneficial effects of PBM on the healing process suggest a promising path for the establishment of this therapeutic modality in the treatment of keloids. Therefore, the purpose of this study is to determine through a randomized and controlled clinical trial the effects of the combination of PBM with blue light and the intralesional administration of the corticoid triamcinolone hexacetonide on the quality of the remaining scar by Vancouver Scar Scale in the postoperative period of keloid surgery. Also, the recurrence rate, its volume, the histopathological analysis, and thequality of life.

## Methods

### Study design

**Fig 1:**
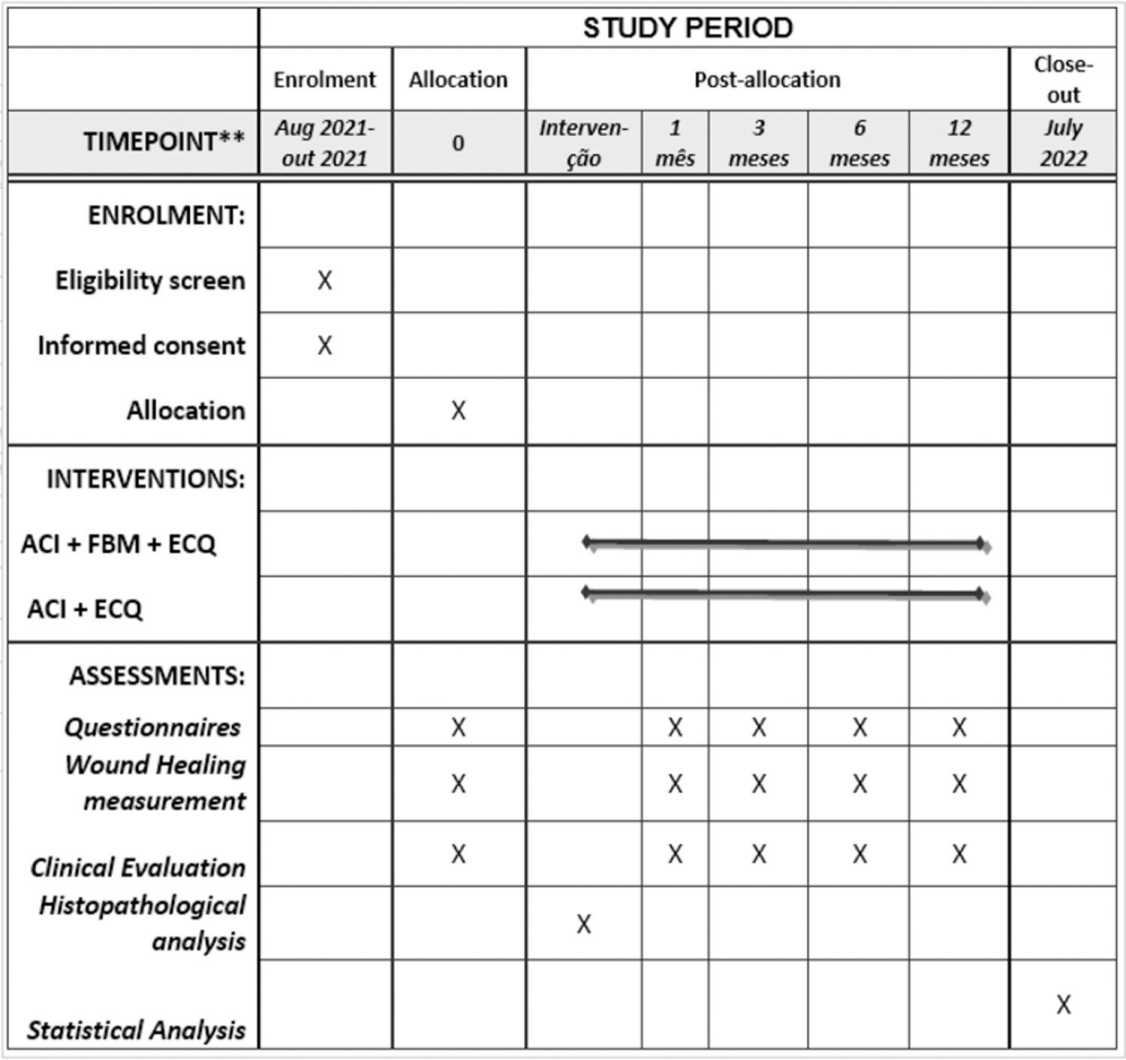
SPIRIT figure as recommended by 2013 SPIRIT statement.

This study protocol was designed as a prospective, randomized, double-blind, controlled trial according to the 2013 SPIRIT (Standard Protocol Items: Recommendations for Interventional Trials) Statement (S7 File) and SPIRIT figure (Fig 1) and will be conducted at Nove de Julho University and Mandaqui Complex Hospital in Brazil in 2021–2023.

### Sample size and participants

The sample size was calculated considering a significance level of 0.05 and 80% power to obtain the means of improvement at three months postoperatively using the Vancouver Scar Scale (corticoid group: mean = 1.95; SD = 1.84) based on the paper by HEWEDY *et al*. [63]. Due to the lack of data in the literature regarding the study group, we estimated the same variability found in Group 1 (G1 1.95 vs. G2 0.25, difference of 1.7). Considering a 10% dropout rate, a total of 58 participants will be recruited: N = 29 per group. All participants will be recruited from the Mandaqui Hospital Complex and Nove de Julho University as well as through announcements in digital media.

#### Inclusion criteria

- Age: 18 to 65 years;
- Fitzpatrick skin phototype I-VI [64]
- Keloid with no type of previous treatment;
- Recurring keloid after surgical excision;
- Recurring keloid after use of other therapies and at least three months without treatment.

The proposed clinical trial (S1 and S2 Files) will be conducted in accordance with the precepts stipulated in the Declaration of Helsinki (revised in Fortaleza, 2014) and was approved by the Ethics committees in human research of the Mandaqui Hospital Complex (number: 4.281.616 (S3 File) and of the *Universidade Nove de Julho*, certificate number: 4.594.799 (S4 File). The participants will receive verbal and written clarifications regarding the objectives and procedures; those who agree to participate in the study will sign a statement of informed consent. After the conclusion of the study, the research data will be available in the thesis databank of *Universidade Nove de Julho* (http://www.uninove.br). The participants’ privacy will be preserved, and their data will be used solely and exclusively for the performance of scientific studies, according to the Confidentiality Term. The information will be published in scientific articles and congresses related to the subject, anonymously.

It is also important to note that all research development will be monitored by the researching surgeon doctor and any complications will be promptly attended to according to technical standards.

#### Exclusion criteria

- Keloid in treatment;
- Pregnant and lactating women;
- Keloid with primary synthesis of the skin and no possibility of excision.
- Contraindications for undergoing surgery (e.g.: coagulopathies, diabetes myellitus, drug allergies);
- Contraindications for the use of corticosteroids;
- All types of Collagenosis.

### Strategies to improve adherence of the participants to the protocol

The participants will be included in a phone message group through which they will be reminded of the follow-up appointments by the researchers. They will also be contacted by the secretaries of the hospital, who will maintain the addresses and contact data of each patient updated.

### Randomization of groups

Block randomization will be performed using a sequence generator program (Research Randomizer, version 4.0, computer software available since June of 2013 at http://www.randomizer.org). The participants will be assigned sequential numbers (1–58). Each patient is going to be allotted an opaque envelop which will contain a piece of paper stipulating to which group it belongs. The envelopes will be sealed until the time of the treatment and their contents will be determined randomly. The patients will be randomly allocated to two groups:The participants will be assigned sequential numbers (1–58). Each patient is going to be allotted an opaque envelop which will contain a piece of paper stipulating to which group it belongs. The envelopes will be sealed until the time of the treatment and their contents will be determined randomly. The patients will be randomly allocated to two groups:

- Study group (N = 29): intralesional administration of corticoid and active photobiomodulation in the preoperative and postoperative periods of keloid surgery.
- Sham group (N = 29): intralesional administration of corticoid and sham photobiomodulation in the preoperative and postoperative periods of keloid surgery.

### Blinding

The evaluator, analyst, and participants will be unaware of the group to which the participants are allocated. The operator will also be unaware of whether the blue light has a biological or is a placebo effect.

## Evaluations

### Questionnaires

A questionnaire will be administered to the participants to collect their personal information. The determination of skin phototype will performed using the Fitzpatrick classification [64] prior to the onset of the interventions in the participant’s first outpatient appointment by trained health professionals and students.

The *Quality of Life of Patients with Keloid and Hypertrophic Scarring* [5] questionnaire, which has been translated and validated for Portuguese, as well as the *QualiFibro/Plastic Surgery—UNIFESP* questionnaire [65] will be used for the assessment of quality of life. The participants will answer the questionnaires prior to the intervention as well as one, three, six, and twelve months postoperatively. The participants will also answer Part II (classification of satisfaction) of the *Patient Scar Assessment Questionnaire* (PSAQ) [66], which has been translated and validated for Portuguese [67], for the assessment of the quality of the remaining scar one, three, six, and twelve months postoperatively. These questionnaires will be applied in the same way in the periods already explained by trained health professionals and students.

Blinded, calibrated, certified plastic surgeons will answer the *Vancouver Scar Scale* (VSS) [68], which was translated into Portuguese by Santos et al. [69], after the clinical evaluation prior to the intervention as well as one, three, six, and twelve months postoperatively.

### Measurement of keloid and remaining scar

Molds will be made of the scars in light condensation silicone (Zhermack, Badia Polesine, Italy). The material will be stored in acrylic dishes and sent for analysis (optical coherence tomography) for the determination of area and volume. These procedures will be performed in preoperatively as well as one, three, six, and twelve months postoperatively.

### Histopathological analysis

Quantitative analysis of the fibroblasts of the keloid specimens will be performed. For such, the histological slides with the samples will be stained with hematoxylin-eosin (H.E.). An analysis of collagen fibers will also be performed using additional slices stained with Picrosirius Red and RNA extraction will be performed for the analysis of TGF-β.

### Statistical analysis

The Kolmogorov-Smirnov test will be used to determine the normality of the data. Variables with parametric distribution will be submitted to one-way analysis of variance (ANOVA) followed by Tukey’s test for comparisons between groups. Variables with nonparametric distribution will be submitted to the Kruskal-Wallis test followed by Dunn’s post hoc text for comparisons between groups. The significance level will be set at α = 5%.

Original study forms will be entered and kept on file at the participating site. When a form is selected, the participating site staff will pull that form, copy it, and sent the copy to the data coordinating center. Participants’ files will be stored in numerical order and stored in a secure and accessible place and manner. They will be maintained in storage for a period of 05 years after completion of the study. The primary author will be responsible for the database on its own device and in the storage cloud. He has no conflicts of interest and no funding.

## Interventions

### Intralesional administration of corticoid

The participants in both groups will receive an intralesional injection of 20 mg/ml triamcinolone hexacetonide (Triancil^®^, Apsen Farmacêutica S.A.)–two injections in the preoperative period with a two-week interval between injections and a monthly injection for three months in the postoperative period. The injections will be intralesional and will not surpass the dermis. The drug will be diluted with the same quantity of 2% lidocaine. The scar will be divided into equal parts of 1 cm^2^ and the drug will be distributed equally, respecting the total dose per session of 20 mg for the face and 40 mg for other topographies.

### Photobiomodulation protocol

PBM will be administered in four sessions with a two-week interval between sessions preoperatively as well as immediately after surgery, weekly in the first month postoperatively, at two-week intervals in the second month, and a single session in the third month. The light source will be blue LED (Quantum, Ecco^®^) using the parameters listed in Table 1. In the sham group, the light will be the same color but without power and, consequently, without any biological effect.

**Table 1:** Dosimetric parameters of photobiomodulation protocol.

Dosimetric parameters	Type of source: LED
Central wavelength [nm]	470
Operating mode	Continuous
Mean radiant power [W]	0.4
Aperture diameter [cm]	1.7
Power density at aperture [W/cm^2^]	0.17
Beam spot on target [cm^2^]	2.268
Irradiance on target [W/cm^2^]	0.176
Useful exposure time [s]	Varying according to size of scar, 60 s per linear cm. Ex.: 1 c m = 60 seg; 10 cm = 600 s.
Exposure time [s]	60 per linear cm
Energy density at aperture [J/cm^2^]	10.58
Radiant energy [J]	Varying with size of scar, according to duration of useful exposure (Item 9). Ex.: 1 cm = 4 J; 10 cm = 240 J
Energy per point [J]	4J
Application technique	Contact
Anatomic location of application points	On remaining scar
Number and frequency of treatment sessions	Preoperative: every two weeks for 30 days. Postoperative: Immediately after surgery, weekly for 4 weeks, every two weeks for another 4 weeks, and one session in 3^rd^ month.

### Surgical procedure

The surgical excision of the keloid will be performed in a standard manner with resections in a spindle shape or ellipse with a 5-mm margin of healthy skin measured after the end of the scar. Regarding depth, the entire scar tissue should be excised until reaching healthy, fibrosis-free tissue. Hemostasis will be performed carefully with low energy to avoid excessive tissue damage.

The suturing of the surgical wound will be performed using thread with little tissue reaction. For subcutaneous tissue, polyglecaprone absorbable thread 25 (Caprofyl^®^, 4–0 diameter) and a circular needle will be used. If necessary, subdermal stitches will be performed with nylon thread (diameter 4–0 to 6–0). Smaller diameters (5–0 and 6–0) will be used at anatomic sites with less tension and thinner skin and the larger diameter (4–0) will be used at sites with greater tension and thicker skin. For the skin, intradermal sutures will be performed using nylon thread with a diameter of 4–0 at anatomic sites with greater tension and thicker skin and 5–0 at anatomic sites without tension and thinner skin. The scar will be cleansed with 0.9% saline solution and dried followed by the placement of micropore strips in an “x” over the entire scar [70]. These procedures will be standardized and performed in the same way in both groups.

## Discussion

The main objective of this study is to determine the effects of the combination of PBM used previous and after the keloid surgery removal associated with the intralesional administration of corticoid on the reduction in the recurrence rate of keloid and the quality of the remaining scar. The hypotheses is that this protocol will improve the distribution and organization of collagen in the scar tissue and diminish the expression of TGF-β, reducing the risk of recurrence of the keloid as well as improving the general appearance of the scar and the quality of life of the patient.

## Supporting information

S1 File Original project approved by ethics committee in research (Portuguese). (DOCX)

S2 File Project approved by ethics committee in research (English). (DOCX)

S3 File Original ethics committee in research of Mandaqui (Portuguese). (DOCX)

S4 File Ethics committee in research of Mandaqui (English). (DOCX)

S5 File Original ethics committee in research of Uninove (Portuguese). (DOCX)

S6 File Ethics committee in research of Uninove (English). (DOCX)

S7 File SPIRIT statement. (DOC)
