# Supplementary material for: Effect of the combination of photobiomodulation therapy and the intralesional administration of corticoid in the preoperative and postoperative periods of keloid surgery: A randomized, controlled, double-blind trial protocol study
Source: PLoS One. 2022 Feb 15;17(2):e0263453. doi: 10.1371/journal.pone.0263453 (PMC8846523; doi:10.1371/journal.pone.0263453)
Supplement: S1 File — (DOCX) [file pone.0263453.s001.docx]

UNIVERSIDADE NOVE DE JULHO

ESTUDO DO EFEITO DA ASSOCIAÇÃO DA TERAPIA DE FOTOBIOMODULAÇÃO E A APLICAÇÃO INTRALESIONAL DE CORTICOIDE NO PRÉ E PÓS-OPERATÓRIO DA EXÉRESE DE QUELOIDES: UM ESTUDO CONTROLADO, RANDOMIZADO E DUPLO-CEGO

JEFFERSON ANDRÉ PIRES

SÃO PAULO

2021

**JEFFERSON ANDRÉ PIRES**

PROJETO DE PESQUISA

ESTUDO DO EFEITO DA ASSOCIAÇÃO DA TERAPIA DE FOTOBIOMODULAÇÃO E A APLICAÇÃO INTRALESIONAL DE CORITCOIDE NO PRÉ E PÓS-OPERATÓRIO DA EXÉRESE DE QUELOIDES: UM ESTUDO CONTROLADO, RANDOMIZADO E DUPLO-CEGO

**Orientadora: Profª Dra. Raquel Agnelli Mesquita Ferrari**

**São Paulo**

**2021**

**RESUMO**

As cicatrizes do tipo queloide são caracterizadas pela proliferação excessiva de fibroblastos e pela quebra do balanço entre a produção e degradação do colágeno, com seu aumento na derme. A gênese dessa patologia ainda não está totalmente elucidada, além dos aspectos genéticos também se sabe a que ela está relacionada ao aumento da expressão de TGF-β. Ainda não existe um tratamento padrão ouro definido, e a recidiva está presente em todos preconizados. O tratamento mais estudado é a aplicação intralesional de corticoide sozinha ou associado no pré e pós-operatório da retirada do queloide. Devido a isso, novas alternativas de tratamento devem ser buscadas. A fotobiomodulação (FBM) com a luz azul tem demonstrado em estudos *in vitro* diminuição da velocidade de multiplicação e da quantidade de fibroblastos bem como do TGF- β. É uma terapia de baixo custo, não invasivo e sem efeitos colaterais, mostrando-se uma boa ferramenta para associar ao tratamento mais preconizado. Dessa maneira o objetivo deste estudo é avaliar o efeito da luz azul associado ao tratamento com corticoide no pré e pós-operatório da exérese de queloides. Será realizado um ensaio clínico randomizado, controlado e duplo cego divididos em dois grupos: 1) Sham (N=29): aplicação intralesional de corticoide (AIC) no pré e pós-operatório da exérese de queloides e 2) FBM associado à AIC (N=29) no pré e pós-operatório da exérese dos queloides. A FBM será realizada de forma pontual transcutânea sobre o queloide no pré-operatório e na cicatriz remanescente no pós operatório utilizando caneta de LED azul (470nm, 400mW, 6,6mJ por ponto, sendo 10 pontos lineares). Os pacientes responderão a dois questionários, um para avaliação da qualidade de vida (Qualifibro-UNIFESP), e um para avaliação da satisfação da cicatriz (PSAQ) e a equipe de cirurgiões plásticos preencherão o questionário de Vancouver para avaliação da cicatriz (VSS), todos serão respondidos com 01, 03, 06 e 12 meses. Os queloides serão moldados no início do tratamento com silicone e antes da ressecção para avaliar o tamanho da área pré e pós-tratamento e da mesma maneira a cicatriz remanescente em 01, 03, 06 e 12 meses do pós-operatório. O queloide retirado será enviado para análises histopatológicas incluindo a quantidade de fibroblastos e a organização e distribuição de colágeno (coloração de picrossirius), e TGF-β. Todos os dados serão submetidos a análise estatística.

**Palavras-chave:** Queloides, Fotobiomodulação, corticoterapia, tratamento cirúrgico.

SUMÁRIO

[1 INTRODUÇÃO 1](#_Toc59456879)

[2 JUSTIFICATIVAS 9](#_Toc59456880)

[3 OBJETIVOS 10](#_Toc59456881)

[4 HIPÓTESES 10](#_Toc59456882)

[5 MATERIAL E MÉTODOS 11](#_Toc59456883)

[5.1 DELINEAMENTO DA PESQUISA 11](#_Toc59456884)

[5.2 AMOSTRAGEM E RANDOMIZAÇÃO 11](#_Toc59456885)

[5.2.1 Critérios de inclusão 11](#_Toc59456886)

[5.2.2 Critério de exclusão 11](#_Toc59456887)

[5.2.3 Composição dos grupos 12](#_Toc59456888)

[5.2.4 Cálculo e tamanho da amostra 12](#_Toc59456889)

[5.2.5 Randomização 13](#_Toc59456890)

[5.3 AVALIAÇÃO PRÉ-TRATAMENTO 14](#_Toc59456891)

[5.3.1 Questionários 14](#_Toc59456892)

[5.3.2 Mensuração da cicatriz ressacad 14](#_Toc59456893)

[5.4 PROCEDIMENTOS PRÉ-OPERATÓRIOS 14](#_Toc59456894)

[5.4.1 Aplicação intralesional do corticoide 14](#_Toc59456895)

[5.4.2 Aplicação da fotobiomodulação 15](#_Toc59456896)

[5.5 PROCEDIMENTO CIRÚRGICO 15](#_Toc59456897)

[5.5.1 Aplicação da anestesia local 16](#_Toc59456898)

[5.5.2 Ato operatório 16](#_Toc59456899)

[5.5.2.1 Procedimento de exérese 16](#_Toc59456900)

[5.5.2.2 Procedimento de hemostasia 16](#_Toc59456901)

[5.5.2.3 Procedimento de síntese da pele 16](#_Toc59456902)

[5.5.2.4 Curativo 17](#_Toc59456903)

[5.6 PROCEDIMENTOS PÓS-OPERATÓRIOS 17](#_Toc59456904)

[5.6.1 Aplicação intralesional do corticoide 17](#_Toc59456905)

[5.6.2 Aplicação da fotobiomodulação 17](#_Toc59456906)

[5.6.3 Questionários 18](#_Toc59456907)

[5.7 ANÁLISE HISTOPATOLÓGICA 18](#_Toc59456908)

[5.7.1 Análise dos fibroblastos 19](#_Toc59456909)

[5.7.2 Análise do colágeno 19](#_Toc59456910)

[5.7.3 Análise do TGF-β 19](#_Toc59456911)

[5.7.3.1 Extração e controle de qualidade de RNA total 19](#_Toc59456912)

[5.7.3.2 Síntese do DNA complementar (CDNA) e PCR em tempo real quantitativo (qPCR) 20](#_Toc59456913)

[5.7.3.3 análise da expressão proteica por ELISA (enzyme-linked immunosorbent assay) 20](#_Toc59456914)

[5.7.3.4 Análise estatística 21](#_Toc59456915)

[6 OBTENÇÃO DAS FOTOGRAFIAS 21](#_Toc59456916)

[7 DESFECHOS 22](#_Toc59456917)

[8 CRONOGRAMA 22](#_Toc59456918)

[9 ORÇAMENTO 23](#_Toc59456919)

[10 REFERÊNCIAS BIBLIOGRÁFICAS 24](#_Toc59456920)

[11 APÊNDICES 33](#_Toc59456921)

[11.1 APÊNDICE 1- TERMO DE CONSENTIMENTO LIVRE E ESCLARECIDO 34](#_Toc59456922)

[11.2 APÊNDICE2- DOCUMENTO PARA PADRONIZAÇÃO DA CIRURGIA. 37](#_Toc59456923)

[11.3 APÊNDICE 3- TERMO DE CONFIDENCIALIDADE 38](#_Toc59456924)

[11.4 APÊNDICE 4- CARTA DE ANUÊNCIA 39](#_Toc59456925)

[12 ANEXOS 40](#_Toc59456926)

[12.1 ANEXO 1- CLASSIFICAÇÃO DO FOTOTIPO DE PELE (FITZPATRICK, 1988) 40](#_Toc59456927)

[12.2 ANEXO 2- QUESTIONÁRIO QUALIFIBRO-UNIFESP (FURTADO, 2008) 41](#_Toc59456928)

[12.3 ANEXO 3- QUESTIONÁRIO PSAQ (OTA, 2016) 42](#_Toc59456929)

[12.4 ANEXO 4- QUESTIONÁRIO ESCALA DE CICATRIZ DE VANCOUVER (SANTOS, 2014) 43](#_Toc59456930)

1 INTRODUÇÃO

O processo de cicatrização é um evento dinâmico, complexo e coordenado que envolvem diversas alças de feedback e circuitos regulatórios de eventos moleculares, celulares e bioquímicos com o fim da regeneração do tecido lesado (CAMPOS et al, 2007). Esse processo pode ser dividido em três fases principais: inflamatória (hemostasia e inflamação), proliferativa (tecido de granulação) e remodelação (maturação) (CLARK, 2005) essas fases se sobrepõem e são mediadas por diversos fatores, como interleucinas, fatores de crescimento, citocinas, vitaminas entre outros (NELIGAN et al, 2016; FERREIRA et al, 2007; POTTER, VEITCH E JOHNSTON, 2019). Tabela 1.

| **FASE** | **DURAÇÃO** | **PRINCIPAIS FATORES E CÉLULAS ENVOLVIDAS** | **PRINCIPAIS EVENTOS** |
| --- | --- | --- | --- |
| **INFLAMATÓRIA** | 48h-72h | Células: Plaquetas, neutrófilos e macrófagos.  Fatores:  TGF-β, tromboxana A2, prostraglandinas, PDGF, FGF, EGF, | Ativação da cascata de coagulação; vasodilatação, quimiotaxia celular, destruição de bactérias, início da fibroplasia e formação da MEC. |
| **PROLIFERATIVA** | 4 a 25 dias | Fibroblastos, VEGF, PDGF, miofibroblastos, queratinócitos, TGF-β, TNF-α. | Epitelização, neoangiogênese, formação de tecido de granulação e deposição de colágeno (tipo III por primeiro e I posteriormente, contração da ferida. |
| **REMODELAÇÃO** | Meses a anos | Colágenos tipo I e III, colagenases. | Organização do colágeno, substituição progressiva do tipo III para o tipo I. |

**Tabela 1-** Fases da cicatrização: Principais fatores e eventos envolvidos. Legenda: TGF-β= Fator transformador beta; PDGF= fator de crescimento derivado das plaquetas; FGF=fator de crescimento derivado dos fibroblastos; EGF=fator de crescimento epidérmico; VEGF= Fator de crescimento endotelial vascular; TNF-α= fator de necrose tumoral alfa; MEC= matriz extra-celular.

Durante as fases de cicatrização qualquer morbidade ou evento que desregule o processo de cicatrização, podem inibir ou prejudicar todo o processo, fazendo com que não haja fechamento da lesão, como em úlceras, ou pelo contrário a exacerbação desses eventos podendo induzir a cicatrizes hiperproliferativas como as hipertróficas e os queloides (NELIGAN et al, 2016; FERREIRA et al, 2007; POTTER, VEITCH E JOHNSTON, 2019). Tabela 2.

| **FATORES QUE INTERFEREM NA CICATRIZAÇÃO** | **PRINCIPAIS EXEMPLOS** |
| --- | --- |
| **LOCAIS** | Infecções; isquemia, presença de corpo estranho; manipulação inadequada, pressão excessiva na cicatriz, irradiação. |
| **SISTÊMICOS** | Depleção proteica, deficiência de vitaminas A, C e complexo B; deficiência de oligoelementos como o Zinco; Diabete Melito, obesidade; quimioterapias e corticoterapias. |
| **SÍNDROMES ASSOCIADAS** | Ehlers-Danlos; homocistenúria; osteogenisis imperfecta. |
| **PREDISPOSIÇÃO ETNICA** | Africanos e asiáticos (queloides). |

**Tabela 2-** Fatores que interferem na cicatrização e seus principais exemplos. (NELIGAN et al, 2016; FERREIRA et al, 2007).

No que se refere as cicatrizes patológicas excessivas, destacam-se as do tipo hipertróficas e queloides. A primeira é relacionada principalmente a má técnica operatória com síntese inadequada dos tecidos e a áreas de maior tensão para o fechamento da pele, como tórax anterior e região dorsal. Apresenta-se restrita na linha da cicatriz e normalmente regridem espontaneamente (NELIGAN et al, 2016; FERREIRA et al, 2007; MAHDAVIAN et al, 2012; WOLFRAM, 2009, LEE PENG E KEROLUS, 2019).

As cicatrizes excessivas do tipo queloide são caracterizadas pela proliferação excessiva de fibroblastos e pela quebra do balanço entre a produção e degradação do colágeno, com seu aumento na derme. Diferentemente das cicatrizes hipertróficas, os queloides não regridem espontaneamente, ultrapassam a linha da cicatriz com crescimento desorganizado, disforme e grosseiro, comparado à uma tumoração benigna, inclusive essa nômina deriva do grego “*kelth”* que significa tumor e *eidoz* que significa forma (FERREIRA *et al,* 2007; WOLFRAM et al, 2009, LIMANDJAJA, NIESSEN, SCHEPER E GIBBS, 2020, NANGOLE E AGAK, 2019).

Além do aspecto estético desagradável, muitas vezes essas cicatrizes são dolorosas, pruriginosas podendo causar incapacidade funcional (por exemplo cicatrizes nas articulações), trazendo como consequência prejuízos psicossociais e na qualidade de vida dos portadores. (BOCK et al, 2006; MOTOKI et al, 2018).

Os aspectos histopatológicos dos quelóides na microscopia de luz incluem um tecido de epitélio fino, uma derme espessada, aumento de fibras colágenas que são desorganizadas e entremeadas com abundante mucina e poucas fibras elásticas. Há um aumento de fibroblastos arredondados e as glândulas sebáceas e os folículos pilosos estão diminuídos ou ausentes. (FERREIRA et al, 2007; PLACIK E LEWIS, 1992; ACKERMAN et al, 1997; KIKUCHI, KADONO E TAKEHARA, 1995; BEER, 2008, LIMANDJAJA, NIESSEN, SCHEPER E GIBBS, 2020).

Na microscopia eletrônica observam-se fibras colágenas com diâmetros reduzidos comparados a uma cicatriz normal, os fibroblastos apresentam actinomiosina em seu citoplasma, sugerindo que muitas dessas células seja uma transição entre os fibroblastos e miofibroblastos. Na histologia também se observam uma predominância do colágeno do tipo I e menor quantidade de colágeno do tipo III, sendo que o tipo III encontra-se aumentado nos quelóides. (FERREIRA et al, 2007; REIS, 1994).

A gênese dessa morbidade ainda não está suficientemente esclarecida, devido principalmente à falta de estudos *in vivo* e por esta patologia não estar descrita em animais dificultando assim o estudo biológico adequado. (WOLFRAN et al, 2009; ARNO et al, 2014; FERREIRA et al, 2007; NELIGAN et al, 2016).

Estudos têm demonstrado que a gênese da hiperproliferação de fibroblastos está relacionada com aspectos inflamatórios (ZHENG-CAI et al, 2020) e com o aumento da expressão do fator de crescimento e transformação β1 (TGF-β1) nas células endoteliais do tecido de neovascularização, que já apresenta expressão aumentada de VEGF e um aumento na expressão do CTGF (fator de crescimento do tecido conectivo). (ARNO et al, 2014; LEE et al, 2017; HAHN et al, 2016; CHIN et al, 2001; FUJIWARA, MURAGAKI E OOSHIMA, 2005; MARNEROS E KRIEG, 2004; COLWELL, 2005; NANGOLE E AGAK, 2019).

O TGF-β tem o papel chave na hiperproliferação de fibroblastos e consequentemente de colágenos. Ele regula a proliferação dessas células bem como a síntese de colágeno. Em um processo de cicatrização normal os níveis de TGF-β reduzem no término do reparo tecidual, contudo nos queloides não diminui, mantendo sua produção alta e desregulada. (WOLFRAM et al, 2009; SARRAZY, 2011; QU, 2012).

Também tem sido demonstrado que nos queloides existe uma quantidade reduzida de enzimas que degradam os componentes da matriz extracelular como as metaloproteinases. Aumento de receptores de PDGF (fator de crescimento derivado de plaquetas) nos fibroblastos, ativação excessiva de sinais para fator de crescimento semelhante à insulina-1 (IGF-1), número reduzido de apoptose de fibroblastos e aumento da expressão de linfopoietina estromal do timo (SHIN JU, KIM SH, KIM H, et al) e ativação de fibrócitos via fator-1 derivado de células estromais são outros achados relacionados a cicatrizes hiperproliferativas. (HAISA, OKOCHI E GROTENDORST, 1994; SAVAH et al 1999)

Alguns estudos mostraram que os queloides podem ter sua gênese predisposta por genes autossômicos dominantes com penetrância incompleta e expressões variadas, contudo não se têm sucesso em isolar um gene ligado diretamente ao queloide. Também tem-se demonstrado em alguns estudos uma maior prevalência no sexo feminino (BROWN E BAYAT, 2009; MARNEROS et al 2001; CHEN et al, 2006; CLARK et al, 2009; SHIH E BAYAT, 2010; NAKASHIMA et al, 2010; NOISHIKI, HAYASAKA Y E OGAWA, 2019).

Existem diversos tratamentos preconizados para o queloide contudo a literatura é controversa visto que muitos dos estudos conduzidos usam metodologia questionável e com resultados imprecisos o que dificulta a elaboração de novos protocolos de tratamentos. Sendo assim, não há nenhum consenso sobre a melhor terapia a ser usada para reduzir o risco de recidiva e que esteja associada a menores efeitos colaterais indesejáveis os quais limitam o uso de muitas terapias. As tabelas 3a e 3b resumem os principais tratamentos utilizados descritos na literatura (ARNO et al, 2014; JAGADEESAN E BAYAT, 2007; HAHN et al, 2016; GOLD, 2014; CHEN et al, 2020; GUPTA E KALRA, 2002; DAVISON et al, 2009; VAN LEEUWEN, VAN LEEUWEN E NIESSEN, 2014; VAN LEEUWEN et al, 2015; HAR-SHAI et al, 2007; JIN et al , 2013; VRIJMAN et al, 2011; VAN DROOGE et al, 2015; EROL et al, 2008; BETARBET E BLALOCK, 2020; LEE et al, 2019).

Os tratamentos buscam principalmente reduzir os sintomas do paciente (dor, prurido e limitações funcionais), reduzir o volume da cicatriz e melhorar a qualidade estética.

| **PRINCIPAIS MONOTERAPIAS** | **EFEITOS COLATERAIS** | **RECIDIVA** |
| --- | --- | --- |
| **RESSECÇÃO CIRÚRGICA** | Complicações mínimas relacionadas ao procedimento cirúrgico. | 45-100% |
| **APLICAÇÃO INTRALESIONAL DE CORTICOIDE**  **(TRIANCINOLONA)** | ulcerações, alterações da coloração da pele, surgimento de telangectasias, relatos de síndrome de Cushing. | <50% |
| **5-FLUORACIL** | Dor, hiperpigmentação, ulcerações. Relato de alopecia transitória. | Similar ao triancil, poucos estudos com uso isolado, geralmente associado a outras terapias. |
| **CRIOTERAPIA** | Hipopigmentação permanente da pele, dor, limitação no uso para paciente com fototipos maiores. | 24% (em torno de 50% respondem positivamente ao tratamento isolado) |
| **LASERS**  **(ablativos)** | Eritema, alteração da pigmentação da pele, cicatrizes após formação de úlceras. | 74-100% usados isoladamente. |

**Tabela 3a-** Principais monoterapias utilizadas para tratamento de queloides (ARNO et al, 2014; JAGADEESAN E BAYAT, 2007; HAHN et al, 2016; SHIN et al, 2016; SHIN E KIM, 2016; GOLD, 2014; CHEN et al, 2020; GUPTA E KALRA, 2002; DAVISON et al, 2009; VAN LEEUWEN, VAN LEEUWEN E NIESSEN, 2014; VAN LEEUWEN et al, 2015; HAR-SHAI et al, 2007; JIN et al , 2013; VRIJMAN et al, 2011; VAN DROOGE et al, 2015; EROL et al, 2008; BETARBET E BLALOCK, 2020; LEE et al, 2019).

| **PRINCIPAIS TERAPIAS COMBINADAS** | **EFEITOS COLATERAIS** | **RECIDIVA** |
| --- | --- | --- |
| **EXÉRESE CIRURGICA + INJEÇÃO DE CORTICOIDES** | As mesmas relacionadas com a aplicação do corticoide somente, depressões na cicatriz, deiscências da ferida. | <30% |
| **EXÉRESE CIRURGICA + RADIOTERAPIA** | Descritos doenças malignas. | Em torno de 22%, varia muito entre os estudos. |

**Tabela 3b-** Principais terapias combinadas utilizadas para tratamento de queloides (ARNO et al, 2014; JAGADEESAN E BAYAT, 2007; HAHN et al, 2016; SHIN et al, 2016; SHIN E KIM, 2016; GOLD, 2014; CHEN et al, 2020; GUPTA E KALRA, 2002; DAVISON et al, 2009; VAN LEEUWEN, VAN LEEUWEN E NIESSEN, 2014; VAN LEEUWEN et al, 2015; HAR-SHAI et al, 2007; JIN et al , 2013; VRIJMAN et al, 2011; VAN DROOGE et al, 2015; EROL et al, 2008; BETARBET E BLALOCK, 2020; LEE et al, 2019).

Dentre os tratamentos citados destacam-se a resseção da lesão com margem, o uso de corticoide injetável e a radioterapia, sendo também utilizados associação das terapias, com a aplicação pré e pós-operatória. Estudos demonstram a remissão dos queloides, contudo muitos deles evidenciando um alto grau de recidiva da doença utilizando todos os tratamentos já preconizados (ARNO et al, 2014; WOLFRAM, 2009; HUU et al, 2019; SHIN et al, 2019; CHEN et al, 2019).

A ressecção cirúrgica com margem, isoladamente não têm resultados bons, demonstrando em estudos até 45% a 100 % de recorrência no pós-operatório (BERMAN, MADERAL E RAPHAEL, 2017). Essas altas recorrências têm sido estudadas e sua principal causa seria a ressecção com margens inadequadas, pois estudos demonstram alta quantidade de fibroblastos em atividade na periferia dos queloides, além de tensão no fechamento da lesão, falta de cuidados na manipulação dos tecidos e má técnica na realização da hemostasia. Mesmo com todos os cuidados a recidiva é alta necessitando terapias adjuvantes no pré e/ou pós-operatórios. (ARNO et al, 2014; WOLFRAM, 2009; SYED et al, 2012; TAN et al, 2010; CHEN et al, 2020; MOHAMMADI, 2019).

A radioterapia tem-se demonstrado efetiva na redução da recorrência do queloide usada pós excisão cirúrgica, comparado ao seu uso isoladamente (MANKOWSKI et al, 2017), contudo não existe nenhum consenso aceito sobre a dose ou modalidade a serem usados no tratamento pós-operatório. Além disso, os riscos potenciais a longo prazo e o sítio anatômico onde será realizado a radiação associado ao custo elevado limitam o seu uso (OGAWA et al, 2009). Relatos de doença maligna após a radiação em queloides já foram reportados na literatura. (ARNO et al, 2014; WOLFRAM, 2009; DE CICCO et al, 2014; OGAWA et al, 2009).

A aplicação intralesional de hexacetonida de triancinolona (Triancil®- Marca registrada de Apsen Farmacêutica S.A), tem sido considerado o tratamento de primeira linha para os queloides, a dose preconizada é de 2,5mg a 20mg em topografia da face e de 20mg a 40mg nas outras regiões do corpo. Seu mecanismo baseia-se na diminuição da síntese de colágeno e glicosaminoglicanas e na inibição da produção dos fibroblastos (SHIN JY et al, 2016). Devido ao seu efeito anti-inflamatório e de vasoconstrição observa-se também a diminuição do prurido e da dor nos pacientes. Estudos demonstram índices de recidiva variáveis até de 50% (ARNO et al, 2014; GOLD, 2014; LEDON et al, 2013; HUU et al, 2019; CHEN et al, 2019).

Não existe nenhum consenso sobre a quantidade e tempo de tratamento com este corticóide sendo que o uso prolongado pode levar a ulcerações, alterações da coloração da pele e surgimento de atelangectasias (ARNO et al, 2014) além de relatos de aparecimento de síndrome de cushing após seu uso (SHIN JY et al, 2016; GOLD, 2014; LEDON et al, 2013; HUU et al, 2019; CHEN et al, 2019).

O uso isolado do corticoide além dos efeitos colaterais já citados muitas vezes não induz a regressão total dos queloides e resultam em cicatrizes residuais não estéticas, com alteração de cor, alargamento, telangectasias e depressões. (GOLD, 2014; LEDON et al, 2013)

A terapia combinada da ressecção cirúrgica com uso do corticoide injetável tem demonstrado ter um bom custo-benefício e segurança com diminuição das taxas de recidiva, em torno de 29%, apesar existir uma grande variação de protocolos e resultados o que dificulta a avaliação precisa. (SCLAFANI et al, 1996; HAMRICK, BOSWELL E CARNEY, 2009; SHONS E PRESS, 1983; BERMAM E FLORES, 1997; KIIL, 1977; JUNG et al, 2009).

Devido à alta incidência de recidiva com o uso dos tratamentos atuais, o quelóide pode permanecer por muito tempo sendo em muitas vezes incapacitante, com diminuição de sua qualidade de vida (MOTOKI et al, 2018). Este fato leva a busca constante por novos produtos e tecnologias minimamente invasivas com o intuito de melhorar o tratamento, com menos efeitos colaterais e menor índice de recidiva.

Neste contexto a terapia de fotobiomodulação (FBM) tem ganhado cada vez mais destaque na literatura. A FBM também conhecida como terapia com luz em baixa intensidade utiliza de fontes de luz de baixa potência, normalmente abaixo de 500mW, com irradiação não ionizante tanto da faixa visível do espectro eletromagnético (400-760nm) como da infra-vermelha (760-1000nm), que agem nos tecidos produzindo efeito biológico positivo nas células (FERNANDES, FERRARI E FRANÇA, 2017; FREITAS E HAMBLIM, 2016).

As respostas fotobiológicas são devidas as mudanças químicas e físicas induzidas pelos fótons nos tecidos biológicos. Cada comprimento de onda irá interagir mais especificamente com um cromóforo (molécula fotorreceptora) específico e essa interação iniciará uma cadeia de reações fotoquímicas e fotofísicas. Um importante cromóforo para comprimentos de onda vermelho e infra-vermelho próxima é a enzima citocromo C oxidase, enzima encontrada na mitocôndria e que participa da cedeia transportadora de elétrons sinalizando caminhos que induzem a produção de óxido nítrico (NO), Adenosina trifosfato (ATP) e espécies reativas de oxigênio (ROS), podendo induzir a transcrição de vários fatores de crescimento como o derivado de plaquetas (PDGF), TGF-β, derivados de fibroblastos (FGF), interleucinas e fator de necrose tumoral alfa (TNF-α), bem como a diminuição ou inibição desses fatores dependendo dos parâmetros dosimétricos adotados (FERNANDES, FERRARI E FRANÇA, 2017; MAMALIS, LEV-TOV, NGUYEN E JAGDEO, 2014; BAROLET E BOUCHER, 2010; SILVA et al, 2010; TRICARICO et al, 2018; HU et al, 2017).

Existem estudos utilizando a FBM em cicatrizes pós-operatórias de diversos tipos de cirurgia demonstrando efeitos benéficos da FBM no processo de cicatrização, mostrando assim a efetividade e segurança dessa terapia. (BAROLET E BOUCHER, 2010; OJEA et al, 2016; FREITAS et al, 2013; ALSHARNOUBI et al, 2018; EPSTEIN et al, 2018; HERASCU et al, 2005; RAMOS et al, 2019; CARVALHO et al, 2010; FUJI et al, 2008; PARK et al, 2016).

Estudos experimentais *in vitro* utilizando fibroblastos de queloides e da derme humana têm demonstrado efeitos positivos incluindo ao aumento na taxa de apoptose e diminuição da velocidade de divisão celular destes fibroblastos, diminuição da síntese de colágeno e da expressão de TFG-β. Nesses estudos, destaca-se a luz azul com comprimento de onda variando de 410-480nm e observa-se efeito inibitório dos fibroblastos e em TGF-β com maiores densidades de energia (0,326 A 640J/cm²). (LEE et al, 2017; HAHN et al, 2019; MAMALIS et al, 2016; MIGNON et al, 2018; OPLÄNDER et al, 2011; HAWKINS E ABRAHAMSE, 2006; LEV-TOV, BRODY, SIEGEL E JAGDEO, 2013; FRIGO et al, 2010; BONATTI et al, 2011).

Estudo experimental recente utilizando a luz azul (410-430nm, densidade de potência 0,69 W/cm²), em diferentes fluências, para irradiar cultura de fibroblastos de queloides retirados de humanos, obteve resultados que corroboram os estudos anteriores, mostrando que nas fluências de maior energia obtiveram como resultados taxas metabólicas menores nos fibroblastos dos queloides. (MAGNI et al, 2020).

É possível que a luz azul interaja com os cromóforos mitocondriais da mesma forma que a luz vermelha e infra-vermelha, uma vez que os centros heme que estão espalhados nos citocromos têm um pico de absorção significativo que coincide com a banda de Soret das porfirinas (HAMBLIN MR, 2018). No espectro da luz azul, flavoproteínas como NADH-dehidrogenase e succinato- desidrogenase, e porfirinas podem funcionar como fotorreceptores (KARU TI, KOLYAKOV SF, 2005).

Estes estudos associados a resultados de efeitos benéficos da FBM em processo de cicatrização sugerem um caminho promissor para o estabelecimento desta modalidade terapêutica no tratamento de queloides.

2 JUSTIFICATIVAS

As cicatrizes patológicas, principalmente as do tipo queloide. São estigmatizantes, de crescimento descontrolado e irregular, pruriginosas e em muitos casos dolorosas, sendo assim, muitas vezes incapacita e limita as atividades diárias e o convívio social de quem é acometido por essa enfermidade.

Os tratamentos mais utilizados atualmente para os queloides estão relacionados a efeitos colaterais indesejáveis, alta recidiva (tentar padronizar o termo) após o tratamento e em nosso meio os altos custo que impossibilitam muitos dos pacientes a terem um tratamento completo. Mesmo com diversos estudos na literatura não há consenso sobre o melhor tratamento ou protocolo a ser adotado no caso de queloides principalmente devido a falhas de delineamento metodológicos, descrição incompleta dos resultados e análise realizadas bem como as próprias incertezas acerca do quelóide que incluem patologia imprevisível e causas ainda mal definidas.

A terapia de FBM com LED ou LLLT surge com uma possibilidade terapêutica devido aos seus efeitos em processo de cicatrização e reparo sendo alternativa de baixo custo, não invasiva, sem efeitos colaterais, de fácil utilização e reprodutibilidade. Mais especificamente a FBM já demonstrou modular de forma positiva o processo inflamatório especificamente TNF-β e colágeno tipo I que estão associados a gênese do queloide. Dessa maneira objetiva-se a associação da FBM no pré e pós-operatório da ressecção cirúrgica de forma a melhorar o processo de cicatrização e a qualidade da cicatriz reduzindo a possibilidade de recidiva.

3 OBJETIVOS

O objetivo principal desse ensaio clínico randomizado duplo cego é verificar os efeitos da combinação da FBM e da aplicação de corticoide intralesional sobre a taxa de recidiva de queloides após ressecção cirúrgica e sobre a qualidade da cicatriz neoformada.

Já os objetivos secundários são avaliar os efeitos da FBM aplicada previamente a ressecção sobre a quantidade e organização de fibroblastos e colágenos, sobre a expressão gênica de TGF-β e sobre a qualidade de vida dos participantes.

4 HIPÓTESES

O protocolo da FBM em combinação com a corticoterapia no pré e no pós-operatório da exérese de queloides melhora a distribuição e organização do colágeno no tecido cicatricial, diminui a expressão do TGF- β reduzindo o risco de recidiva e desta forma melhora o aspecto geral da cicatriz e melhora a qualidade de vida do participante.

5 MATERIAL E MÉTODOS

5.1 DELINEAMENTO DA PESQUISA

Será conduzido um ensaio clínico randomizado, duplo-cego (paciente, equipe e analista) e controlado, no período de dezembro de 2020 a março de 2023, em pacientes com queloide atendidos no ambulatório do Serviço de Cirurgia Plástica do Conjunto Hospitalar do Mandaqui e também recrutados a partir do Ambulatório de Especialidades da Universidade Nove de Julho (UNINOVE) na cidade de São Paulo-SP. Este ensaio clínico foi redigido em conformidade com a Declaração de Helsinki (revisada em Fortaleza, 2014) e será submetido ao Comitê de Ética e Pesquisa da Universidade Nove de Julho (Uninove) e do Conjunto Hospitalar do Mandaqui (CHM).

Após explicação verbal e por escrito do estudo, os participantes que aceitarem o convite para participar da pesquisa assinarão o termo de consentimento livre e esclarecido (TCLE) (Apêndice 1).

5.2 AMOSTRAGEM E RANDOMIZAÇÃO

Serão selecionados voluntários de ambos os sexos com idade entre 18 e 65 anos do Ambulatório de especialidades do Conjunto Hospitalar do Mandaqui e da Universidade Nove de Julho, na cidade de São Paulo que apresentem queloides e anseio do tratamento dele.

5.2.1 Critérios de inclusão

Serão incluídos na pesquisa pessoas entre 18 e 65 anos, com fototipo de pele Fitzpatrick I-VI, apresentando queloides sem nenhum tipo de tratamento prévio, ou em recidivas de exérese cirúrgica apenas ou recidiva após uso de outras terapias com pelo menos 03 meses sem estar utilizando qualquer tratamento, atendidos no ambulatório do Serviço de Cirurgia Plástica do Conjunto Hospitalar do Mandaqui, na cidade de São Paulo-SP.

5.2.2 Critério de exclusão

Serão excluídos do estudo pacientes fora da faixa etária estudada, bem como com queloides com qualquer tipo de tratamento em vigência, pacientes grávidas e lactantes, queloides que não tenham a possibilidade de ressecção com síntese primária da pele, pacientes com colagenoses, alergias as medicações utilizadas, contraindicação ao uso de corticosteroides e contraindicações a realização de cirurgia (Ex.: Coagulopatias, alergias, qualquer tipo de diabetes).

5.2.3 Composição dos grupos

Os pacientes serão distribuídos de maneira randomizada em dois grupos: **A) ESTUDO:** Grupo ACI + FBM + ECQ: Fotobiomodulação (FBM) e aplicação de corticoide no pré e pós-operatórios da exérese cirúrgica do queloide (ECQ).

**B) CONTROLE (SHAM):** Grupo ACI + ECQ: Aplicação de corticoide injetável (ACI) no pré e pós-operatórios da exérese cirúrgica do queloide (ECQ). A aplicação da FBM será simulada com aparelho idêntico ao do grupo estudo, com caneta com a mesma cor de luz, contudo sem potência e consequentemente sem efeito.

5.2.4 Cálculo e tamanho da amostra

O cálculo amostral foi realizado para mostrar uma diferença de médias entre dois grupos: grupo1: pacientes com uso de corticoides e grupo2: pacientes com uso de corticoides e fotobiomodulação em quielóides.

Usando um nível de significância igual a 0,05 e um poder de 80%, e se baseando no artigo de HEWEDY *et al* (2020) para obter as médias de melhora da cicatrização pós cirúrgicas, em 3 meses, pela Vancouver scar scale (grupo corticóide: média=1,95; DP=1,84). Devido à falta de dados na literatura quanto ao grupo 2 estimamos a mesma variabilidade encontrada no grupo 1. Sendo definido o tamanho amostral conforme mostra a tabela 4.

| Sem 10 % de perdas | |  | Com 10 % de perdas | |  |  |
| --- | --- | --- | --- | --- | --- | --- |
| Média G1 vs Média G2  (3 meses pós) | Diferença | n_Total_ | n_Grupo_ |  | n_Total_ | n_Grupo_ |
| 1,95 vs 0,25 | 1,7 | 38 | 19 |  | 58 | 29 |

**Tabela 4-** Tamanho da amostra com e sem 10% de perdas.

5.2.4.1 Metodologia do cálculo da amostra

As variáveis categóricas serão representadas pela frequência absoluta e relativa. As variáveis quantitativas serão representadas por média e desvio-padrão ou mediana e intervalo interquartílico (mediana [P25; P75]) de acordo com a distribuição verificada pelo teste de normalidade de Shapiro-Wilk. Quando um dos grupos obteve tamanho (n) menor que 12 sujeitos as variáveis quantitativas serão representadas diretamente por mediana e intervalo interquartílico e comparados por testes não paramétricos.

As variáveis no tempo pré serão comparadas da seguinte forma: as proporções das variáveis estudadas serão comparadas entre os grupos pelo teste de qui-quadrado. As comparações das médias das variáveis quantitativas entre aos grupos serão realizadas pelo teste t para amostras independentes ou pelo teste de Mann-Whitney.

Para comparar os grupos e os tempos das médias das variáveis quantitativas estudadas (por exemplo: escore Vancouver, área da cicatrização etc.) será realizado o modelo de Equações de Estimações Generalizadas (*GEE Model*) (LIANG E ZEGER, 1986; ZEGER E LIANG, 1986; GUIMARÃES E HIRAKATA, 2012). Essa análise será realizada já que a amostras são relacionadas, ou seja, o mesmo paciente responde em vários momentos distintos as mesmas medidas. Será testado o efeito principal de grupo, tempo e a interação dupla dos fatores (grupo*tempo). O modelo será composto por uma matriz de correlação trabalho *unstructured*, uma matriz de covariância de estimador robusto e uma distribuição conforme resultado do teste de normalidade (normal ou gamma) com uma função ligação adequada (identidade ou logarítmica). Quando significativo o teste post-hoc de Bonferroni será utilizado para identificar as categorias distintas.

As análises serão realizadas no software IBM SPSS Statistics v.25. O nível de significância adotado será de 0,05. As análises gráficas serão realizadas no Excel® 2013.

5.2.5 Randomização

Será realizada uma randomização em bloco utilizando um programa gerador de sequências não determinadas (Reserch Randomizer, versão 4.0 [software de computador], disponível desde junho de 2013 em <http://www.randomizer.org>).

5.3 AVALIAÇÃO PRÉ-TRATAMENTO

5.3.1 Questionários

Serão aplicados três questionários para os participantes contendo informações de identificação e um questionário para os especialistas após avaliação clínica. O primeiro deles será para classificação do fototipo de pele através da classificação de Fitzpatrick (FITZPATRICK, 1988) (Anexo 1).

Por segundo será aplicado o questionário *Quality of Life of Patients With Keloid And Hypertrophic Scarring* (BOCK *et al.*, 2006), que foi traduzido e validado para o português e denominado questionário QualiFibro/Cirurgia Plástica-UNIFESP (FURTADO,2008) (Anexo 2).

Por último o paciente irá responder a parte II (classificação de satisfação) do questionário *Patient Scar Assessment Questionnaire* (PSAQ) (DURANI, MCGROUTHER, FERGUSON, 2009), que foi traduzido e valido paro o português e denominado questionário de avaliação da cicatriz pelo paciente (OTA, 2016) (Anexo 3).

Também será respondido por especialistas cegados e calibrados o questionário Vancouver Scar Scale (VSS), publicado por Sullivan et al (1990) e traduzido por Santos et al (2014) (Anexo 4).

5.3.2 Mensuração da cicatriz ressacad**a**

Serão obtidos moldes das cicatrizes, com silicone leve de condensação (Zhermack, Badia Polesine, Italia). O material será acondicionado em placas acrílicas e encaminhadas para análise de tomografia de coerência óptica, com obtenção da área e volume. O procedimento será repetido após a terapia pré-operatória e com 03, 06 e 12 meses do pós-operatório.

5.4 PROCEDIMENTOS PRÉ-OPERATÓRIOS

5.4.1 Aplicação intralesional do corticoide

Os participantes dos grupos estudo e controle (Sham) serão submetidos a aplicação de do corticoide hexacetonida de triancinolona (Triancil®- Apsen Farmacêutica S.A), com duas aplicações com intervalos de duas semanas antes da cirurgia de exérese do queloide. As aplicações serão realizadas com seringas para aplicação de insulina de 1ml com agulha fixa, intralesional sem ultrapassar a derme.

A medicação utilizada tem apresentação de 20mg/ml, será diluída com a mesma quantidade de lidocaína 2%. A cicatriz será divindade em partes iguais de 1cm² e distribuída igualmente, respeitadas as doses totais por sessão de 20mg para face e de 40 mg para as demais topografias.

5.4.2 Aplicação da fotobiomodulação

A fotobiomodulação será realizada em quatro sessões no pré-operatório com intervalos de duas semanas utilizando a fonte de luz de LED azul (Sistema de laserterapia, modelo Quantum, marca Ecco®) conforme os parâmetros dosimétricos listados na tabela X.

| **PARÂMETROS DOSIMÉTRICOS** | **TIPO DE FONTE: LED** |
| --- | --- |
| Comprimento de onda central [nm] | 470 |
| Modo de operação | Continuo |
| Potência radiante média [mW] | 400 |
| Diâmetro de abertura [cm] | 1,7 |
| Densidade de potência na abertura [W/cm2] | 0,17 |
| Área do feixe no alvo [cm2] | 2,268 |
| Irradiância no alvo [W/cm2] | 0,4 |
| Duração da exposição útil [s] | Varia de acordo com o tamanho da cicatriz, máximo 600. |
| Duração da exposição [s] | 60 por ponto |
| Densidade de energia na abertura [J/cm2] | 105,8 considerando exposição máxima |
| Energia radiante [mJ] | 240.000 considerando exposição máxima |
| Energia por ponto [mJ] | 6,66 |
| Técnica de aplicação | Contato |
| Localização anatômica dos pontos de aplicação | Na cicatriz remanescente 01 ponto por cm² |
| Número e frequência das sessões de tratamento | Semanalmente por 4 semanas. |

**Tabela 5-** Parâmetro dosimétricos que serão utilizados no pré-operatório.

5.5 PROCEDIMENTO CIRÚRGICO

Todos os procedimentos cirúrgicos serão realizados em centro cirúrgico hospitalar com todos os cuidados preconizados para segurança do paciente. Será encaminhado uma carta à equipe de Cirurgia Plástica do Conjunto Hospitalar do Mandaqui com a padronização do procedimento para os participantes do estudo.

5.5.1 Aplicação da anestesia local

Será utilizado uma solução de anestésico local na concentração de 1:100.000, contendo 20 ml de lidocaína a 2% (20mg/ml) sem vasoconstritor, 20 ml de cloridrato de bupivacaína 0,5% (5ml/ml), 1ml de epinefrina (1mg/ml) e 60ml de soro fisiológico (0,9%). A solução poderá sofrer alterações se for necessário adequação para o peso do paciente, obedecendo o uso de 5mg/kg de lidocaína e 3mg/km de bupivacaína.

Após todos os cuidados com antissepsia e assepsia, a solução será aplicada com seringa estéril e descartável de 10ml com agulhas de 13x45mm para superfície e agulhas de 25x7mm para a profundidade. A quantidade de anestésico será dividida proporcionalmente em toda extensão da cicatriz, sendo injetado quantidades iguais em áreas de 1cm².

5.5.2 Ato operatório

Todos os procedimentos cirúrgicos serão realizados sob os preceitos básicos da técnica cirúrgica adequada, obedecendo de maneira rigorosa os tempos de diérese, hemostasia e síntese.

5.5.2.1 Procedimento de exérese

Para a ressecção do queloide serão utilizadas lâminas de bisturi de nº15, sendo ressecados em forma de fuso ou elipse com margens de 5mm, de pele saudável, medida após o término da cicatriz. Quanto a profundidade, deverá ser ressecado toda a cicatriz até obtenção de tecido saudável, livre de fibrose.

5.5.2.2 Procedimento de hemostasia

A hemostasia deverá ser realizada com eletrocautério monopolar ou bipolar em intensidades baixas (até 30). Deverá ser realizada de maneira judiciosa somente onde há sangramento e se necessário para evitar lesão tecidual desnecessária.

5.5.2.3 Procedimento de síntese da pele

Para a síntese da ferida operatória serão usados fios com menor reação tecidual. Para o tecido celular subcutâneo, quando necessário se usará o fio absorvível a base de poliglecaprone 25 (caprofyl®), com diâmetro 4-0 e agulha circular. Os pontos subdérmicos se necessário deverá utilizar fios de nylon com diâmetro de 4-0 a 6-0, onde os diâmetros mais finos (5-0 e 6-0) serão utilizados em sítios anatômicos com menos tensão e pele mais fina e o de maior diâmetro (4-0) em sítios com maior tensão e pele mais espessa.

Para a síntese da pele será utilizado sutura do tipo intradérmica com fio de nylon de diâmetros de 4-0 e 5-0, sendo o primeiro em áreas com maior tensão e derme mais espessa e o segundo em sítios anatômicos sem tensão e com derme mais fina.

5.5.2.4 Curativo

Após o procedimento de síntese, a cicatriz será limpa com solução de soro fisiológico 0,9% e seca. Após será realizado a microporagem (colocação de fitas microporadas em “x” em toda a extensão da cicatriz (HOCHMAN B, ISHIZUKA CK, FERREIA LM, OLIVEIRA LQR, LOCALI RF, 2004).

5.6 PROCEDIMENTOS PÓS-OPERATÓRIOS

5.6.1 Aplicação intralesional do corticoide

Os participantes de ambos os grupos serão submetidos a aplicação de do corticoide hexacetonida de triancinolona (Triancil®- Apsen Farmacêutica S.A), com aplicações mensais com intervalo de 30 dias até completar 03 aplicações. As aplicações serão realizadas com seringas para aplicação de insulina de 1ml com agulha fixa, intralesional sem ultrapassar a derme.

A medicação utilizada tem apresentação de 20mg/ml, será diluída com a mesma quantidade de lidocaína 2%. A cicatriz será divindade em partes iguais de 1cm² e distribuída igualmente, respeitadas as doses totais por sessão de 20mg para face e de 40 mg para as demais topografias.

5.6.2 Aplicação da fotobiomodulação

A fotobiomodulação será realizada semanalmente no primeiro mês de pós-operatório, a cada 15 dias no segundo mês de pós-operatório e uma aplicação no 3º mês de pós-operatório, utilizando a fonte de luz de LED azul azul (Sistema de laserterapia, modelo Quantum, marca Ecco®) conforme os parâmetros dosimétricos listados na tabela 6.

| **PARÂMETROS DOSIMÉTRICOS** | **TIPO DE FONTE: LED** |
| --- | --- |
| Comprimento de onda central [nm] | 470 |
| Modo de operação | Continuo |
| Potência radiante média [mW] | 400 |
| Diâmetro de abertura [cm] | 1,7 |
| Densidade de potência na abertura [W/cm2] | 0,17 |
| Área do feixe no alvo [cm2] | 2,268 |
| Irradiância no alvo [W/cm2] | 0,4 |
| Duração da exposição útil [s] | Varia de acordo com o tamanho da cicatriz, máximo 600. |
| Duração da exposição [s] | 60 por ponto |
| Densidade de energia na abertura [J/cm2] | 105,8 considerando exposição máxima |
| Energia radiante [mJ] | 240.000 considerando exposição máxima |
| Energia por ponto [mJ] | 6,66 |
| Técnica de aplicação | Contato |
| Localização anatômica dos pontos de aplicação | Na cicatriz remanescente 01 ponto por cm² |
| Número e frequência das sessões de tratamento | Imediatamente após o término da cirurgia, semanalmente por 4 semanas, a cada quinze dias por mais 4 semanas e uma aplicação no 3ª mês. |

**Tabela 6-** Parâmetro dosimétricos que serão utilizados no pós-operatório.

5.6.3 Questionários

O paciente responderá novamente o questionário QualiFibro/Cirurgia Plástica-UNIFESP (Anexo 2) e a parte II de satisfação da cicatriz do PSAQ (Anexo 3) em 01, 03, 06 e 12 meses do pós-operatório.

Os especialistas treinados e cegados responderão o VSS (Anexo 4) novamente em 01, 03, 06 e 12 meses do pós-operatório.

5.7 ANÁLISE HISTOPATOLÓGICA

Os queloides ressecados na cirurgia serão armazenados em solução de formol a 10% neutro e tamponado para em seguida serem processados e obtidos cortes histológicos que serão corados com H&E e picrosirius para análise de aspectos morfológicos teciduais.

5.7.1 Análise dos fibroblastos

Será realizada uma análise quantitativa dos fibroblastos dos queloides ressacados, os preparados histológicos serão submetidos à técnica de coloração pela Hematoxilina-Eosina (H.E.).

As imagens histológicas serão fotografadas com câmera digital acoplada ao microscópio, sob foco fixo e clareza de campo, obtendo-se 20 campos por lâmina com aumento de 400x. As fotografias serão escolhidas aleatoriamente e analisadas através de software de computador.

5.7.2 Análise do colágeno

Cortes adicionais serão corados com PicrosiriusRed (Sigma-Aldrich, St. Louis, MO, EUA) e examinados por microscopia de luz polarizada Pol-Interferencial Photomicroscope (Modelo 61282, Carl Zeiss, Alemanha). As imagens também serão analisadas pelo programa Image J (NIH). Com auxílio deste programa, a área relativa ocupada pelas fibras colágenas será calculada em relação à área total do corte (Junqueira et al., 1982; Ribeiro et al., 2015; Andreo et al., 2018).

5.7.3 Análise do TGF-β

5.7.3.1 Extração e controle de qualidade de RNA total

Para a extração de RNA total das amostras será utilizado entre 80-100 mg de tecido macerado em nitrogênio líquido. Em seguida, o tecido será homogeneizado em 1 mL do reagente TRIzol (Invitrogen, São Paulo, Brasil), para iniciar o isolamento do RNA total, seguindo as orientações do fabricante. A concentração do RNA total e pureza será determinada em NanoDrop 2000 (Thermo Scientific) utilizando os comprimentos de onda 260 e 280 nm e sua relação e as amostras serão armazenadas a -80°C. Para análise da integridade do RNA total, 1 μg deste será analisado por meio de eletroforese em gel de agarose 1% e corado com brometo de etídeo.

5.7.3.2 Síntese do DNA complementar (CDNA) e PCR em tempo real quantitativo (qPCR)

O RNA total será submetido à reação de transcrição reversa utilizando-se o *High Capacity cDNA Reverse Transcriptions Kit* (Applied Biosystems) para a obtenção do cDNA e este será utilizado para a reação de PCR em tempo real utilizando o “SYBR Green Kit” (Applied Biosystems). Para este procedimento as amostras serão analisadas em triplicata. As reações serão realizadas utilizando-se o termociclador 7500 Real-Time PCR System (Applied Biosystems Carlsbad, CA, USA) e oligonucleotídeos iniciadores (“*primers forward e reverse*”) específicos para o TGF-β e para o constitutivo GAPDH serão usados para a realização deste procedimento. A quantificação será realizada utilizando o método 2 − ∆∆CT (Livak & Schmittgen, 2001) e o grupo controle será usado como referência.

Tabela x. Primer utilizado

| **Primer** | **Forward (5’ – 3’)** | **Reverse (3’ – 5’)** |
| --- | --- | --- |
| TGF-β | TCCAACCCAGGTCCTTCCTAAAGT | CCCCTGGAAAGGGCTCAACAC |

5.7.3.3 análise da expressão proteica por ELISA (enzyme-linked immunosorbent assay)

Os extratos musculares também serão utilizados para análise de expressão proteica sendo obtidos pela maceração do tecido muscular em nitrogênio líquido e homogeneizados com tampão de extração RIPA e fluoreto de fenilmetilsulfonil (PMSF) 0.1 mM. O extrato tecidual será centrifugado a 10.000 rpm por 10 minutos a 4 °C e o sobrenadante será armazenado a -80°C até o momento da análise. A quantificação de proteína total será realizada em NanoDrop 2000 (Thermo Scientific), utilizando os comprimentos de onda de 260 e 280 nm. A expressão proteica do TGF-β nos extratos musculares será avaliada por meio do ensaio imunoenzimático ELISA utilizando kits comerciais TGF-β (R&D Systems, Minneapolis, EUA) seguindo as instruções do fabricante.

5.7.3.4 Análise estatística

A distribuição de normalidade dos dados será avaliada pelo teste de Kolmogorov-Smirnov. Os dados com distribuição paramétrica serão submetidos ao teste One-way ANOVA seguido pelo teste de Tukey para comparação entre os grupos. Os dados com distribuição não paramétrica serão submetidos ao teste Kruskal-Wallis seguido pelo teste de Dunn’s para a comparação entre grupos. O nível de significância adotado será de α=5%.

6 OBTENÇÃO DAS FOTOGRAFIAS

No consultório na avaliação inicial, mantendo todos preceitos éticos e de privacidade do paciente serão realizadas fotografias digitais da cicatriz do tipo queloide nas seguintes incidências: anterior; posterior, superior e laterais esquerda e direita com intuito de dar uma visão global da cicatriz, será colocada régua milimétrica no ato da fotografia. Será utilizado uma mesma câmera digital para todas as fotografias, utilizando iluminação com LED branco, com mesmo padrão de cor de fundo, distante 20 cm da cicatriz em modo macro manual, e com a mesma distância foca, sendo seguido o mesmo padrão na avaliação de 3, 6, e 12 meses. Cada cicatriz será fotografada em 5 incidências totalizando 20 fotografias por paciente no período estudado.

As imagens serão armazenadas em computador e disco virtual, mantendo todos os preceitos de segurança e com acesso apenas do pesquisador.

7 DESFECHOS

Os desfechos esperados com o protocolo estudado de acordo com as variáveis seriam:

1. Diminuição da área do queloide ressecado em comparação ao pré-tratamento na análise da modelagem pela tomografia de coerência óptica;
2. Melhora do aspecto da cicatriz e a não recidiva através da avaliação clínica especializadas através do questionário de avaliação de cicatriz de Vancouver (VSS);
3. Melhora da qualidade de vida do paciente verificado através do questionário Qualifibro-UNIFESP;
4. Satisfação do paciente quando a cicatriz residual, verificada através da parte de avaliação de satisfação do questionário PSAQ;
5. Diminuição e organização dos fibroblastos e colágeno e TGF-β através da análise histopatológica dos queloides ressacados.

8 CRONOGRAMA

| **EVENTO** | **DATA INICIAL** | **DATA FINAL** |
| --- | --- | --- |
| Apresentação do protocolo em eventos | 21/12/2020 | 31/12/2021 |
| Experimento | 01/12/2021 | 31/07/2021 |
| Análise dos resultados | 01/12/2021 | 31/07/2022 |
| Redação de artigos científicos | 01/07/2022 | 31/03/2023 |
| Recrutamento dos participantes | 01/09/2021 | 30/11/2021 |
| Revisão da literatura | 21/12/2020 | 31/07/2022 |
| Organização do banco de dados | 01/01/2022 | 31/12/2022 |

9 ORÇAMENTO

| **DESCRIÇÃO** | **VALOR UNITÁRIO** | **QUANTIDADE** | **TOTAL** |
| --- | --- | --- | --- |
| Caixa de luvas de procedimento descartáveis 100UI | 25,00 | 02 | 50,00 |
| Caixa de luvas cirúrgicas estéreis descartáveis 50UI | 80,00 | 02 | 160,00 |
| Bisturis cirúrgicos descartáveis nº 15 | 4,60 | 60 | 276,00 |
| Agulhas descartáveis 18G  12 x 40mm | 0,20 | 100 | 20,00 |
| Agulhas descartáveis 26G  4,5 x 13mm | 0,20 | 100 | 20,00 |
| Seringas descartáveis de 3ml Luer Lock | 0,50 | 100 | 50,00 |
| Frascos para biopsia | 1,00 | 200 | 200,00 |
| Formaldeído 10% 1L | 45,00 | 2 | 90,00 |
| Caixa de Máscara descartável com 50UI | 10,00 | 04 | 40,00 |
| Caixa de Touca descartável com 100UI | 10,00 | 01 | 10,00 |
| Clorexidine alcoolica 0,5% em 100mL | 15,00 | 10 | 150,00 |
| Lidocaína 2% + epinefrina | 8,00 | 25 | 200,00 |
| Micropore bege hipoalergênico | 25,00 | 5 | 125,00 |
| Impressos |  |  | 250,00 |
| Gaze estéril | 0,50 | 200 | 100,00 |
| Análise estatística |  | 1 | 2500,00 |
| Soro fisiológico 0,9% 10ml | 0,20 | 100 | 20,00 |
| Custos com esterilização | 5,00 | 30 | 150,00 |
| Caixa de lâminas foscas para microscópico 26x76mm 50UI | 20,00 | 03 | 60,00 |
| Fio de sutura de poligrecrapone-25 ou similar | 12,00 | 60 | 720,00 |
| Fio de sutura de Nylon | 2,00 | 60 | 120 |
| Triancil 20mg/ml | 14,00 | 116 | 1624,00 |
| TOTAL |  |  | 6935,00 |

10 REFERÊNCIAS BIBLIOGRÁFICAS

ACKERMAN, B et al. Fibrosing dermatites In: Histologic diagnosis of inflammatory skin diseases: na algorithimic method based on patern analysis. 2ed Baltimore, williams e Wilkins. P.727-33, 1997.

ANDREO L, MESQUITA-FERRARI RA, RIBEIRO ÃBG, BENITTE A. Effects of Myogenic Precursor Cells (C2C12) Transplantation and Low-Level Laser Therapy on Muscle Repair. Lasers Surg Med. 2018; 50(7):781–91.

ALSHARNOUBI et al. Evaluation of scars in children after treatment with low-level laser. Lasers Med Sci. 2018. 33**,** 1991–1995.

ARNO AI, GAUGLITZ GG, BARRET JP, JESCHKE MG. Up-to-date approach to manage keloids and hypertrophic scars: a useful guide. Burns. 2014;40(7):1255-66.

BAROLET D, BOUCHER A. Prophylactic low-level light therapy for the treatment of hypertrophic scars and keloids: a case series. Lasers Surg Med 2010; 42:597-601.

BEER TW, LAM MH, HEENAN PJ. Tumors of fibrous tissue involving the skin. In: Lever's Histopathogy of the Skin, 10th ed, Elder DE (Ed), Wolters-Kluwer, Lippincott, Williams, & Wilkins, Philadelphia 2008. p.969.

BERMAN B, FLORES F. Recurrence rates of excised keloids treated with postoperative triamcinolone acetonide injections or interferon alfa-2b injections. J Am Acad Dermatol. 1997 Nov;37(5 Pt 1):755-7.

BERMAN B, MADERAL A, RAPHAEL B. Keloids and hypertrophic Scars: pathophysiology, classification, and treatment. Dermatol Surg. 2017;43(Suppl 1):S3–S18.

BETARBET U, BLALOCK TW. Keloids: A Review of Etiology, Prevention, and Treatment. *J Clin Aesthet Dermatol*. 2020;13(2):33‐43.

BOCK O, SCHMID-OTT G, MALEWSKI P, MROWIETZ U. Quality of life of patients with keloid and hypertrophic scarring. Arch Dermatol Res 2006;297:433–438.

BROWN JJ, BAYAT A. Genetic susceptibility to raised dermal scarring. Br J Dermatol 2009; 161:8.

BONATTI S, HOCHMAN B, TUCCI-VIEGAS VM, et al. In vitro effect of 470 nm LED (Light Emitting Diode) in keloid fibroblasts. Acta Cirurgica Brasileira. 2011 Feb;26(1):25-30.

CAMPOS, ACL, BORGES-BRANCO, A, GROTH, AK. Cicatrização de feridas. ABCD, arq. bras. cir. dig., São Paulo, v. 20, n. 1, p. 51-58, Mar.  2007.

CARVALHO, ALCÂNTARA, KAMAMOTO, CRESSONI E CASAROTTO. Effects of low-level laser therapy on pain and scar formation after inguinal herniation surgery: a randomized controlled single-blind study. Photomed Laser Surg. 2010 Jun;28(3):417-22.

CHEN AD, CHEN RF, LI YT, HUANG YT, LIN SD, LAI CS, KUO YR. Triamcinolone Acetonide Suppresses Keloid Formation Through Enhancing Apoptosis in a Nude Mouse Model. Ann Plast Surg. 2019 83(4S Suppl 1):S50-S54.

CHEN B, DING J, JIN J, SONG N, LIU Y. Continuous tension reduction to prevent keloid recurrence after surgical excision: preliminary experience in Asian patients [published online ahead of print, 2020 May 10]. *Dermatol Ther*. 2020; e13553. doi:10.1111/dth.13553.

CHEN Y, GAO JH, LIU XJ, et al. Characteristics of occurrence for Han Chinese familial keloids. Burns 2006; 32:1052.

CHIN GS, LIU W, PELED Z, LEE TY, STEINBRECH DS, HSU M, et al. Differential expression of transforming growth factor- beta receptors I and II and activation of Smad 3 in keloid fibroblasts. Plast Reconstr Surg 2001; 108:423-429.

CLARK RAF: Wound repair. In: Kumar, Robbins, Cotran: Pathologic Basis of Disease, 7th ed., Ed. Saunders, p.112, 2005.

CLARK JA, TURNER ML, HOWARD L, et al. Description of familial keloids in five pedigrees: evidence for autosomal dominant inheritance and phenotypic heterogeneity. BMC Dermatol 2009; 9:8.

COLWELL AS, PHAN TT, KONG W, et al. Hypertrophic scar fibroblasts have increased connective tissue growth factor expression after transforming growth factor-beta stimulation. Plast Reconstr Surg 2005; 116:1387.

DAVISON SP, DAYAN JH, CLEMENS MW, et al. Efficacy of intralesional 5-fluorouracil and triamcinolone in the treatment of keloids. Aesthet Surg J 2009; 29:40.

DE CICCO L, VISCHIONI B, VAVASSORI A, GHERADI F, JERECZEK BA, LAZZARI R, et al. Postoperative management of Keloids: low-dose-rate and high-dose-rate brachyterapy. Brachytherapy. 2014; 13(5): 508.

DE FREITAS LF, HAMBLIN MR. Proposed Mechanisms of Photobiomodulation or Low-Level Light Therapy. *IEEE J Sel Top Quantum Electron*. 2016;22(3):7000417. doi:10.1109/JSTQE.2016.2561201

DURANI P, MCGROUTHER DA, FERGUSON MW. The Patient Scar Assessment Questionnaire: a reliable and valid patient-reported outcomes measure for linear scars. Plast Reconstr Surg. 2009 May;123(5):1481-9.

EPSTEIN et al. Photobiomodulation Therapy Alleviates Tissue Fibroses Associated with Chronic Graft-Versus-Host Disease: Two Case Reports and Putative Anti-Fibrotic Roles of TGF-β. Photomed Laser Surg. 2018 Feb;36(2):92-99.

EROL OO, GURLEK A, AGAOGLU G, et al. Treatment of hypertrophic scars and keloids using intense pulsed light (IPL). Aesthetic Plast Surg 2008; 32:902.

FERNANDES KPS, FERRARI RAM, FRANÇA CM. Biofotônica: Conceitos e Aplicações. São Paulo: Universidade Nove de Julho; 2017.

FERREIRA LM. Guias de medicina ambulatorial e hospitalar da UNIFESP/EPM: Cirurgia Plástia. Barueri: Manole; 2007.

FITZPATRICK TB. The validity and practicality of sun-reactive skin types I through VI. Arch Dermatol. 1988 Jun;124(6):869-71.

FREITAS, MELO, ALEXANDRINO E NOITES. Efficacy of low-level laser therapy on scar tissue, Journal of Cosmetic and Laser Therapy. 2013 15:3, 171-176.

FRIGO L, FÁVERO GM, LIMA HJ, et al. Low-level laser irradiation (InGaAlP-660 nm) increases fibroblast cell proliferation and reduces cell death in a dose-dependent manner. Photomed Laser Surg. 2010 Aug;28 Suppl 1:S151-6.

FUJII et al., low reactive level laser therapy (lllt) for the treatment of hypertrophic scars and keloids. A re-introduction. Laser Therapy. 2008, 17(1): 35-43.

FUJIWARA M, MURAGAKI Y, OOSHIMA A. Keloid-derived fibroblasts show increased secretion of factors involved in collagen turnover and depend on matrix metalloproteinase for migration. Br J Dermatol 2005; 153:295.

FURTADO FMP. Tradução para o idioma português, adaptação cultural e confiabilidade do Questionnaire of Quality of Life for Patients with Keloid and Hypertrophic Scarring [dissertação]. São Paulo: Escola Paulista de Medicina, Universidade Federal de São Paulo, 2008.

GOLD MH, MCGUIRE M, MUSTOE TA et al. Updated international clinical recommendations on scar management: part 2—algorithms for scar prevention and treatment. Dermatol Surg. 2014;40(8):825–831.

GUIMARÃES, LSP; HIRAKATA, VN. Use of the generalized estimating equation model in longitudinal data analysis. Revista HCPA. Porto Alegre. Vol. 32, n. 4 (2012), p. 503-511.

GUPTA S, KALRA A. Efficacy and safety of intralesional 5-fluorouracil in the treatment of keloids. *Dermatology*. 2002;204(2):130‐132.

HAHN JM et al. Partial epithelial-mesenchymal transition in keloid scars: regulation of keloid keratinocyte gene expression by transforming growth factor-β1. Burns Trauma 4(1):30. 2016

HAMBLIN MR. Mechanisms and Mitochondrial Redox Signaling in Photobiomodulation. Photochem Photobiol. 2018;94(2):199-212.

HAMRICK M, BOSWELL W, CARNEY D. Successful treatment of earlobe keloids in the pediatric population. J Pediatr Surg 2009; 44:286.

HAN B, FAN J, LIU L, et al. Adipose-derived mesenchymal stem cells treatments for fibroblasts of fibrotic scar via downregulating TGF-β1 and Notch-1 expression enhanced by photobiomodulation therapy. *Lasers Med Sci*. 2019;34(1):1‐10.

HAISA M, OKOCHI H, GROTENDORST GR. Elevated levels of PDGF alpha receptors in keloid fibroblasts contribute to an enhanced response to PDGF. J Invest Dermatol 1994; 103:560.

[HAR-SHAI Y, DUJOVNY E, ROHDE E, ZOUBOULIS CC. Effect of skin surface temperature on skin pigmentation during contact and intralesional cryosurgery of keloids. J Eur Acad Dermatol Venereol 2007; 21:191.](https://www.uptodate.com/contents/keloids-and-hypertrophic-scars/abstract/69)

HAWKINS D, ABRAHAMSE H. Effect of multiple exposures of low-level laser therapy on the cellular responses of wounded human skin fibroblasts. Photomed Laser Surg. 2006 Dec;24(6):705-14.

HERASCU, VELCIU, CALIN, SAVASTRU E TALIANU. Low-level laser therapy (LLLT) efficacy in post-operative wounds. Photomed Laser Surg. 2005 Feb;23(1):70-3.

HEWEDY ES, SABAA BEI, MOHAMED WS, HEGAB DS. Combined intralesional triamcinolone acetonide and platelet rich plasma versus intralesional triamcinolone acetonide alone in treatment of keloids [published online ahead of print, 2020 Mar 4]. *J Dermatolog Treat*. 2020;1-7.

HOCHMAN B, ISHIZUKA CK, FERREIA LM, OLIVEIRA LQR, LOCALI RF. Revisão. Estima – Brazilian Journal of Enterostomal Therapy, *[S. l.]*, v. 2, n. 3, 2004. Disponível em: https://www.revistaestima.com.br/estima/article/view/154. Acesso em: 15 nov. 2020.

HU Y, ZHANG C, LI S, JIAO Y, QI T, WEI G, HAN G. Effects of Photodynamic Therapy Using Yellow LED-light with Concomitant Hypocrellin B on Apoptotic Signaling in Keloid Fibroblasts. Int J Biol Sci. 2017;13(3):319-326.

HUU ND, HUU SN, THI XL, VAN TN, MINH PPT, MINH TT, et. al. Successful Treatment of Intralesional Triamcilonon Acetonide Injection in Keloid Patients. Open Access Maced J Med Sci. 2019; 28;7(2): 275-278.

JAGADEESAN J, BAYAT A. Transforming growth factor beta (TGFbeta) and keloid disease. Int J Surg 2007; 5:278-285.

JIN R, HUANG X, LI H, et al. Laser therapy for prevention and treatment of pathologic excessive scars. Plast Reconstr Surg 2013; 132:1747.

JUNG JY, ROH MR, KWON YS, CHUNG KY. Surgery and perioperative intralesional corticosteroid injection for treating earlobe keloids: a korean experience. Ann Dermatol 2009; 21:221.

JUNQUEIRA LCU, MONTES GS, SANCHEZ EM. The influence of tissue section thickness on the study of collagen by the Picrosirius-polarization method. Histochemistry. 1982; 74(1):153–6.

KARU TI, KOLYAKOV SF. Exact action spectra for cellular responses relevant to phototherapy. Photomed Laser Surg. 2005;23(4):355-61.

KIIL J. Keloids treated with topical injections of triamcinolone acetonide (kenalog). Immediate and long-term results. Scand J Plast Reconstr Surg 1977; 11:169.

KIKUCHI K, KADONO T. TAKEHARA K. effects of various growth factors and histamine on cultured keloid fibroblastos. Dermatology, 190: 4-8, 1995.

LIANG K-Y, ZEGER SL. Longitudinal data analysis using generalized linear models. Biometrika. 1986;73(1):13-22.

LEE HS, JUNG SE, KIM SK, KIM YS, SOHN S, KIM YC. Low-Level Light Therapy with 410 nm Light Emitting Diode Suppresses Collagen Synthesis in Human Keloid Fibroblasts: An In Vitro Study. Ann Dermatol. 2017;29(2):149-155.

LEE PENG G, KEROLUS JL. Management of Surgical Scars. Facial Plast Surg Clin North Am. 2019 Nov;27(4):513-517

LEE YI, KIM J, YANG CE, HONG JW, LEE WJ, LEE JH. Combined Therapeutic Strategies for Keloid Treatment. Dermatol Surg. 2019 Jun;45(6):802-810.

LEDON JA, SAVAS J, FRANCA K et al. Intralesional treatment for keloids and hypertrophic scars: a review. Dermatol Surg. 2013;39(12):1745–1757.

LEV-TOV H, BRODY N, SIEGEL D, JAGDEO J. Inhibition of fibroblast proliferation in vitro using low-level infrared light-emitting diodes. Dermatol Surg. 2013 Mar;39(3 Pt 1):422-5.

LIMANDJAJA GC, NIESSEN FB, SCHEPER RJ, GIBBS S. The Keloid Disorder: Heterogeneity, Histopathology, Mechanisms and Models. Front Cell Dev Biol. 2020 May 26;8:360.

LIVAK KJ, SCHMITTGEN TD. Analysis of relative gene expression data using real-time quantitative PCR and the 2-ΔΔCT method. Methods. 2001; 25(4):402–8.

MAGNI G, BANCHELLI M, CHERCHI F, et al. Experimental Study on Blue Light Interaction with Human Keloid-Derived Fibroblasts. Biomedicines. 2020 Dec 6;8(12):573.

MAHDAVIAN DELAVARY B, VAN DER VEER WM, FERREIRA JA, NIESSEN FB. Formation of hypertrophic scars: evolution and susceptibility. J Plast Surg Hand Surg 2012; 46:95.

MANKOWSKI P, KANEVSKY J, TOMLINSON J, et al. Optimizing Radiotherapy for Keloids: A Meta-Analysis Systematic Review Comparing Recurrence Rates Between Different Radiation Modalities. Ann Plast Surg 2017; 78:403.

MAMALIS, A., KOO, E., GARCHA, M., MURPHY, W. J., ISSEROFF, R. R., & JAGDEO, J. (2016). High fluence light emitting diode-generated red light modulates characteristics associated with skin fibrosis. Journal of Biophotonics, 9(11-12), 1167–1179.

MAMALIS AD, LEV-TOV H, NGUYEN DH, JAGDEO JR. Laser and light-based treatment of Keloids--a review. J Eur Acad Dermatol Venereol 2014; 28:689-699.

MARNEROS AG, KRIEG T. Keloids--clinical diagnosis, pathogenesis, and treatment options. J Dtsch Dermatol Ges 2004; 2:905.

MARNEROS AG, NORRIS JE, OLSEN BR, REICHENBERGER E. Clinical genetics of familial keloids. Arch Dermatol 2001; 137:1429.

MIGNON C, UZUNBAJAKAVA NE, CASTELLANO-PELLICENA I, BOTCHKAREVA NV, TOBIN DJ. Differential response of human dermal fibroblast subpopulations to visible and near-infrared light: Potential of photobiomodulation for addressing cutaneous conditions. Lasers Surg Med. 2018 Oct;50(8):859-882.

MOHAMMADI AA, KARDEH S, MOTAZEDIAN GR, SOHEIL S. Management of Ear Keloids Using Surgical Excision Combined with Postoperative Steroid Injections. *World J Plast Surg*. 2019;8(3):338‐344.

MOTOKI THC, ISOLDI FC, BRITO MJA, FILHO AG, FERREIRA LM. Keloid negatively affects body image. *Burns*. 2019;45(3):610‐614.

NAKASHIMA M, CHUNG S, TAKAHASHI A, et al. A genome-wide association study identifies four susceptibility loci for keloid in the Japanese population. Nat Genet 2010; 42:768.

NANGOLE FW, AGAK GW. Keloid pathophysiology: fibroblast or inflammatory disorders? JPRAS Open. 2019 Dec;22:44-54.

NELIGAM PC, GURTNER GC. Cirurgia Plástica: Princípios. Tradução: Facina T et al. 3ªed, Vol 1. Rio de Janeiro: Elsevier; 2015.

NOISHIKI C, HAYASAKA Y, OGAWA R. Sex Differences in Keloidogenesis: An Analysis of 1659 Keloid Patients in Japan. Dermatol Ther (Heidelb). 2019 Dec;9(4):747-754.

OGAWA R, YOSHITATSU S, YOSHIDA K, MIYASHITA T. Is radiation therapy for keloids acceptable? The risk of radiation-induced carcinogenesis. Plast Reconstr Surg 2009; 124:1196.

OJEA AR, MADI O, NETO RM et al. Beneficial Effects of Applying Low-Level Laser Therapy to Surgical Wounds After Bariatric Surgery. Photomed Laser Surg. 2016 Nov;34(11):580-584.

OPLÄNDER C, HIDDING S, WERNERS FB, BORN M, PALLUA N, SUSCHEK CV. Effects of blue light irradiation on human dermal fibroblasts. J Photochem Photobiol B. 2011 May 3;103(2):118-25.

OTA AS. Tradução para a língua portuguesa, adaptação cultural para o Brasil e validação do patient scar assessment questionnaire. 2016. 155f. Dissertação (mestrado)- Escola Paulista de Medicina, Universidade Federal de São Paulo (UNIFESP). São Paulo, 2016.

PARK et al. Prevention of Thyroidectomy Scars in Asian Adults With Low-Level Light Therapy. Dermatol Surg. 2016 Apr;42(4):526-34.

PLACIK, OJ, LEWIS, VL. Immunologic associations of keloids. Surg. Gynecol.obstet. 175: 186-93, 1992.

POTTER DA, VEITCH D, JOHNSTON GA. Scarring and wound healing. Br J Hosp Med (Lond). 2019 Nov 2;80(11):C166-C171

[QU L, LIU A, ZHOU L, et al. Clinical and molecular effects on mature burn scars after treatment with a fractional CO(2) laser. Lasers Surg Med 2012; 44:517.](https://www.uptodate.com/contents/keloids-and-hypertrophic-scars/abstract/19)

RAMOS et al. Photobiomodulation Improved the First Stages of Wound Healing Process After Abdominoplasty: An Experimental, Double-Blinded, Non-randomized Clinical Trial. Aesthetic Plast Surg. 2019 Feb;43(1):147-154.

REIS, ALN. Principais características das cicatrizes queloideanas. Na bras dermatol 69 (6) 495-7, 1994.

SANTOS MC, TIBOLA J, MARQUES CMG. Traduação, revalidação e confiabilidade de Cicatrização de Vancouver para língua portuguesa – Brasil. Rev Bras Queimaduras. 2014; 13:26-30.

SARRAZY V, BILLET F, MICALLEF L, et al. Mechanisms of pathological scarring: role of myofibroblasts and current developments. Wound Repair Regen 2011; 19 Suppl 1:s10.

SAYAH DN, SOO C, SHAW WW, et al. Downregulation of apoptosis-related genes in keloid tissues. J Surg Res 1999; 87:209.

SHIH B, BAYAT A. Genetics of keloid scarring. Arch Dermatol Res 2010; 302:319.

SHIN J, CHO JT, PARK SI, JUNG SN. Combination therapy using non-ablative fractional laser and intralesional triamcinolone injection for hypertrophic scars and keloids treatment. Int Wound J. 2019; 1-7.

SHIN JU, KIM SH, KIM H, et al. TSLP Is a Potential Initiator of Collagen Synthesis and an Activator of CXCR4/SDF-1 Axis in Keloid Pathogenesis. J Invest Dermatol 2016; 136:507.

SHIN JY, LEE JW, ROH SG, LEE NH, YANG KM. A Comparison of the Effectiveness of Triamcinolone and Radiation Therapy for Ear Keloids after Surgical Excision: A Systematic Review and Meta-Analysis. Plast Reconstr Surg. 2016;137(6):1718-25.

SCLAFANI AP, GORDON L, CHADHA M, ROMO T 3º. Prevention of earlobe keloid recurrence with postoperative corticosteroid injections versus radiation therapy: a randomized, prospective study and review of the literature. Dermatol Surg 1996; 22:569.

SHONS AR, PRESS BH. The treatment of earlobe keloids by surgical excision and postoperative triamcinolone injection. Ann Plast Surg 1983; 10:480.

SILVA JP, DA SILVA MA, ALMEIDA AP, LOMBARDI JUNIOR I, MATOS AP. Laser therapy in the tissue repair process: a literature review. Photomed Laser Surg 2010;28:17-21.

SULLIVAN T, SMITH J, KERMODE J, MCLVER E, COURTEMANCHE DJ. Rating the burn scar. J Burn Care Rehabil. 1990 May-Jun;11(3):256-60.

SYED F, AHMADI E, IQBAL SA et al. Fibroblasts from the growing margin of keloid scars produce higher levels of collagen I and III compared with intralesional and extralesional sites: clinical implications for lesional site-directed therapy. Br J Dermatol. 2011;164(1):83–96.

TAN KT, SHAH N, PRITCHARD SA et al. The influence of surgical excision margins on keloid prognosis. Ann Plast Surg. 2010;64(1):55–58.

TRICARICO PM, ZUPIN L, OTTAVIANI G, PACOR S, JEAN-LOUIS F, BONIOTTO M, et al. Photobiomodulation therapy promotes in vitro wound healing in nicastrin KO HaCaT cells. J Biophotonics. 2018;11(12): 1-10.

VAN DROOGE AM, VRIJMAN C, VAN DER VEEN W, WOLKERSTORFER A. A randomized controlled pilot study on ablative fractional CO2 laser for consecutive patients presenting with various scar types. Dermatol Surg 2015; 41:371.

VAN LEEUWEN MC, BULSTRA AE, VAN LEEUWEN PA, NIESSEN FB. A new argon gas-based device for the treatment of keloid scars with the use of intralesional cryotherapy. J Plast Reconstr Aesthet Surg 2014; 67:1703.

VAN LEEUWEN MC, VAN DER WAL MB, BULSTRA AE, et al. Intralesional cryotherapy for treatment of keloid scars: a prospective study. Plast Reconstr Surg 2015; 135:580.

VRIJMAN C, VAN DROOGE AM, LIMPENS J, et al. Laser and intense pulsed light therapy for the treatment of hypertrophic scars: a systematic review. Br J Dermatol 2011; 165:934.

WOLFRAM D, TZANKOV A, PÜLZL P, PIZA-KATZER H. Hypertrophic scars and keloids--a review of their pathophysiology, risk factors, and therapeutic management. Dermatol Surg. 2009 Feb;35(2):171-81.

ZEGER SL, LIANG KY. Longitudinal data analysis for discrete and continuous outcomes. Biometrics. 1986;42(1):121-30.

ZHENG-CAI W, WAN-YI Z, YANGYANG C, et al. The Roles of Inflammation in Keloid and Hypertrophic Scars. Frontiers in Immunology. 2020; 11: 3185.

11 APÊNDICES


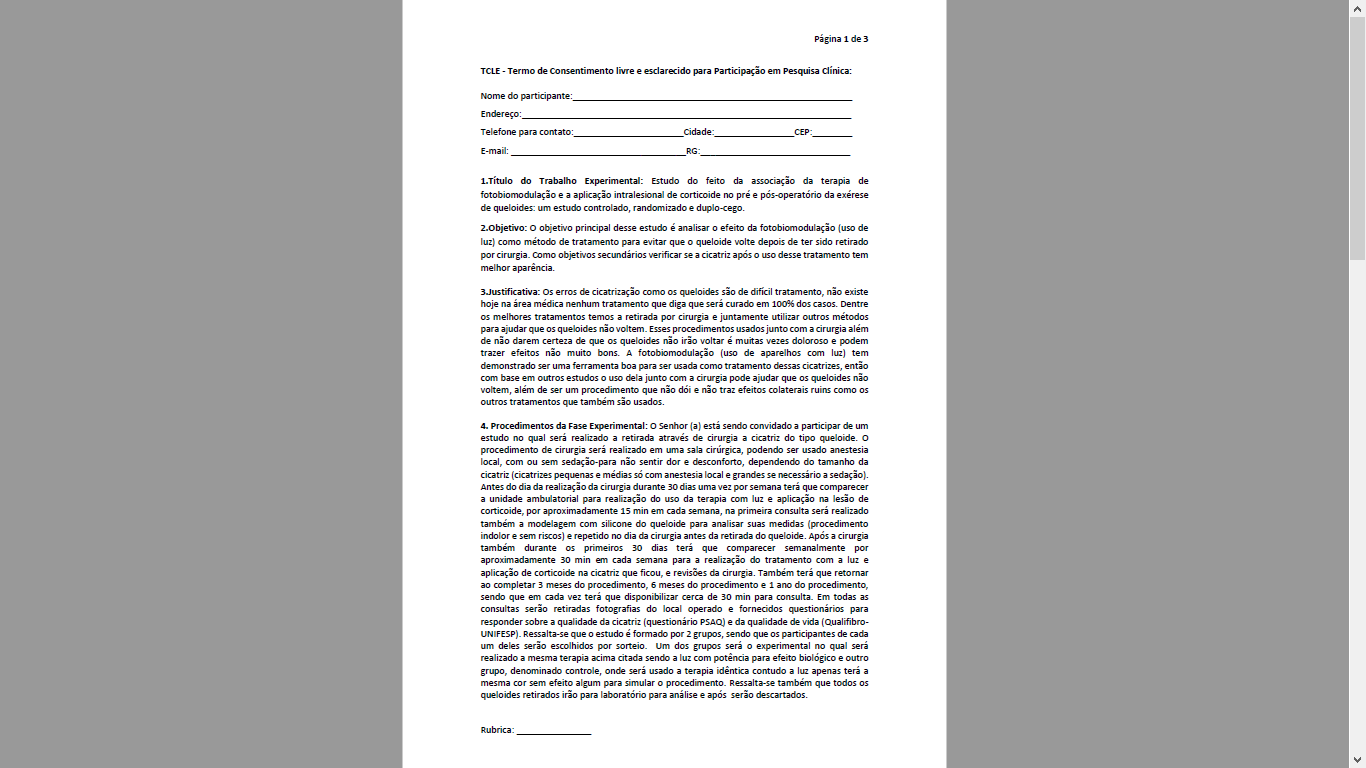
11.1 APÊNDICE 1- TERMO DE CONSENTIMENTO LIVRE E ESCLARECIDO


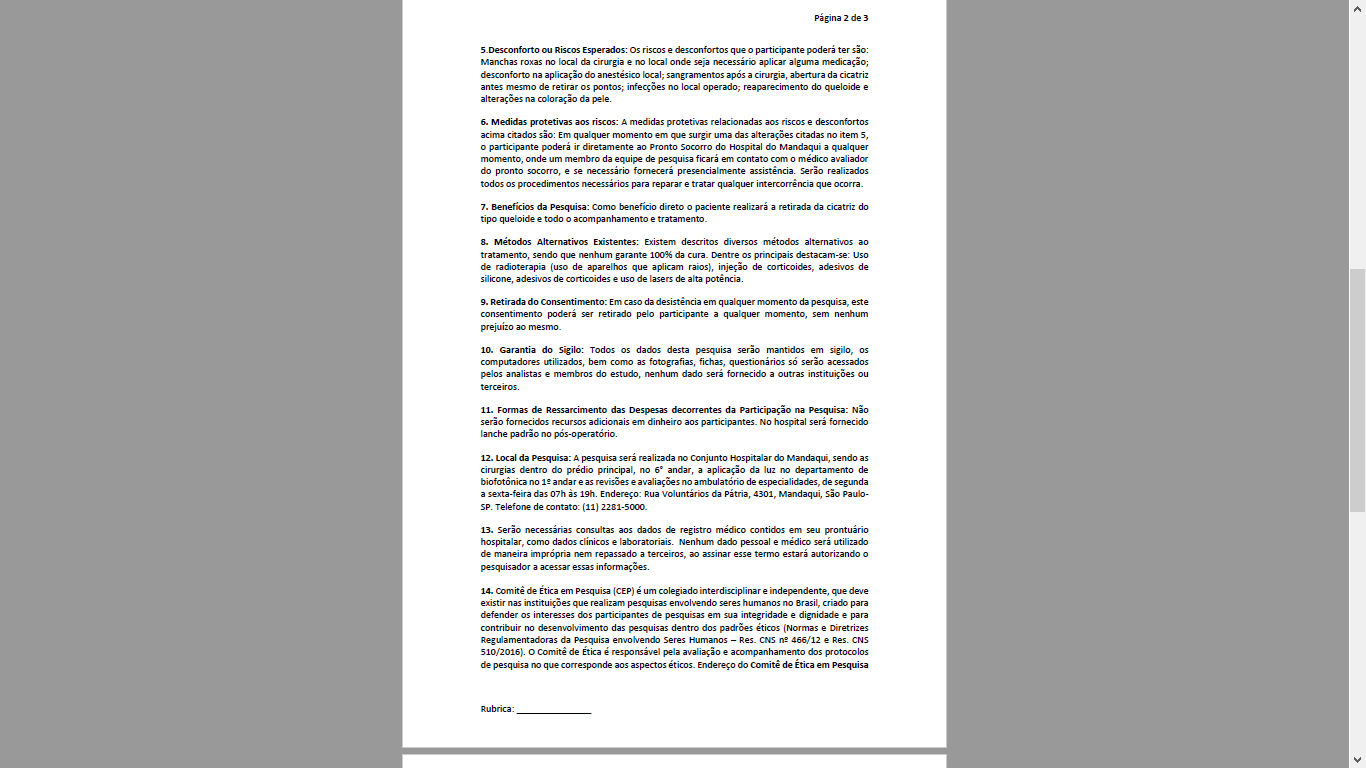


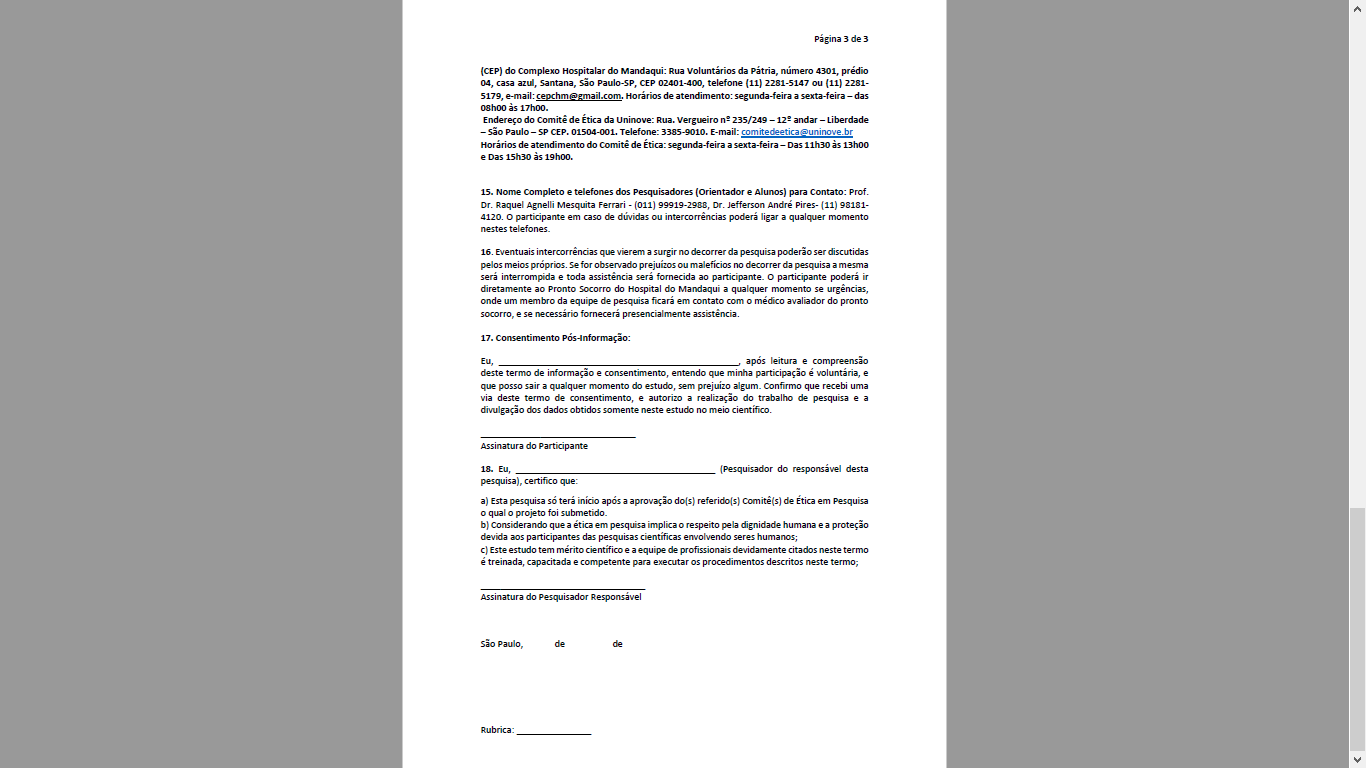


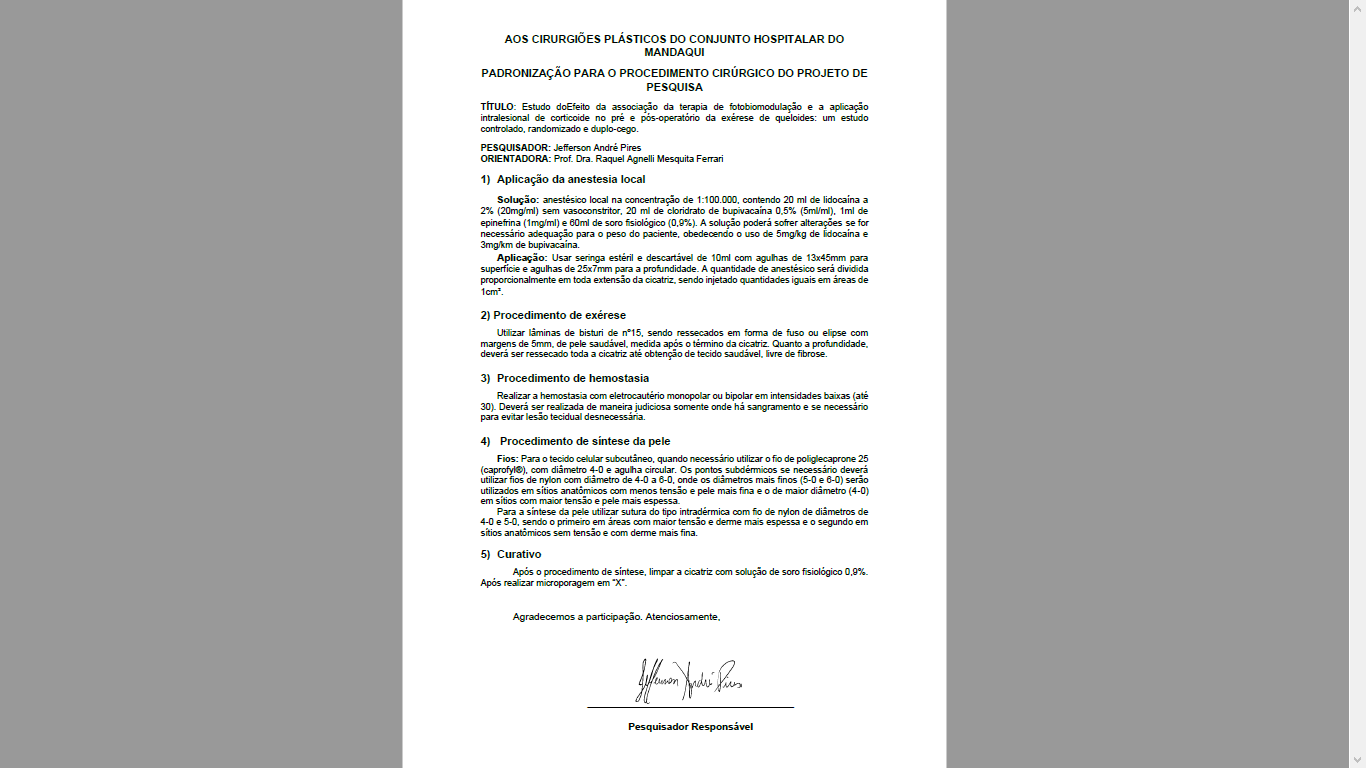
11.2 APÊNDICE2- DOCUMENTO PARA PADRONIZAÇÃO DA CIRURGIA.


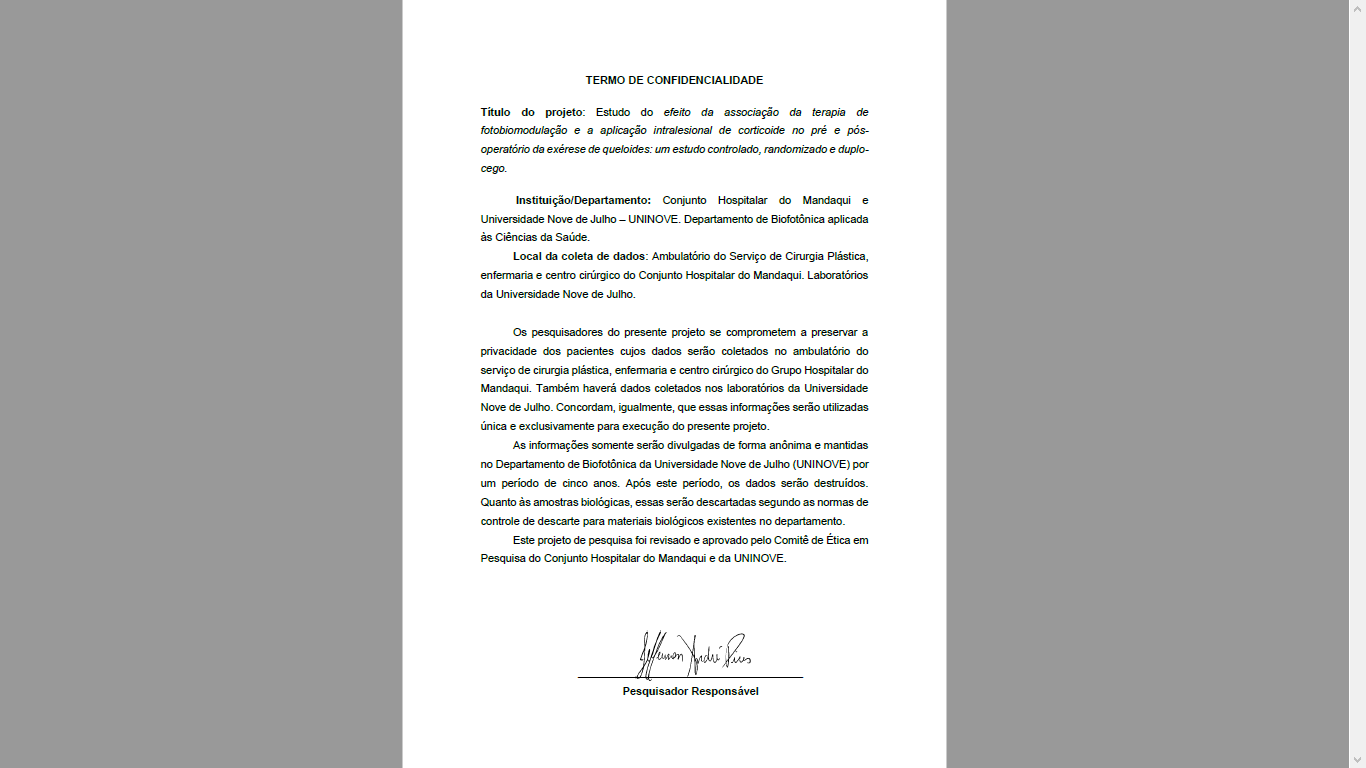
11.3 APÊNDICE 3- TERMO DE CONFIDENCIALIDADE


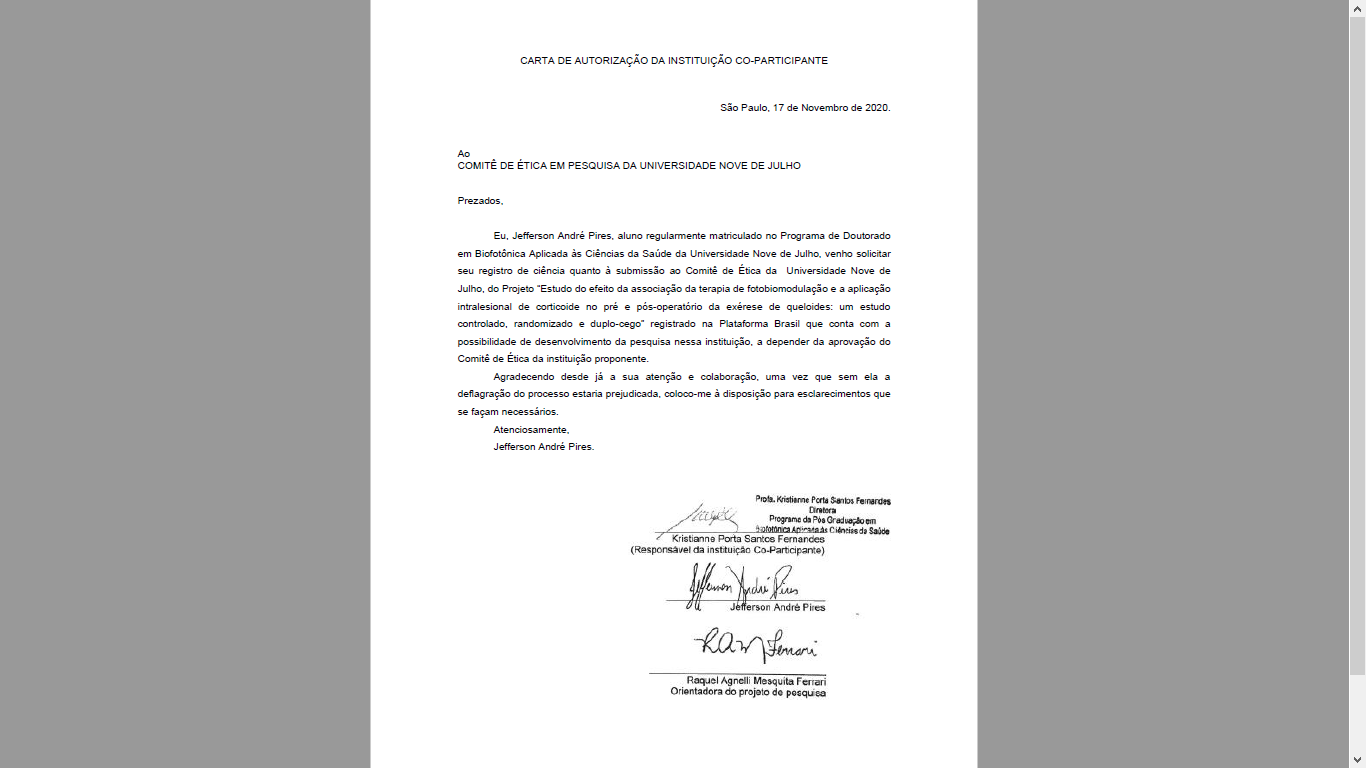
11.4 APÊNDICE 4- CARTA DE ANUÊNCIA

12 ANEXOS

12.1 ANEXO 1- CLASSIFICAÇÃO DO FOTOTIPO DE PELE (FITZPATRICK, 1988)


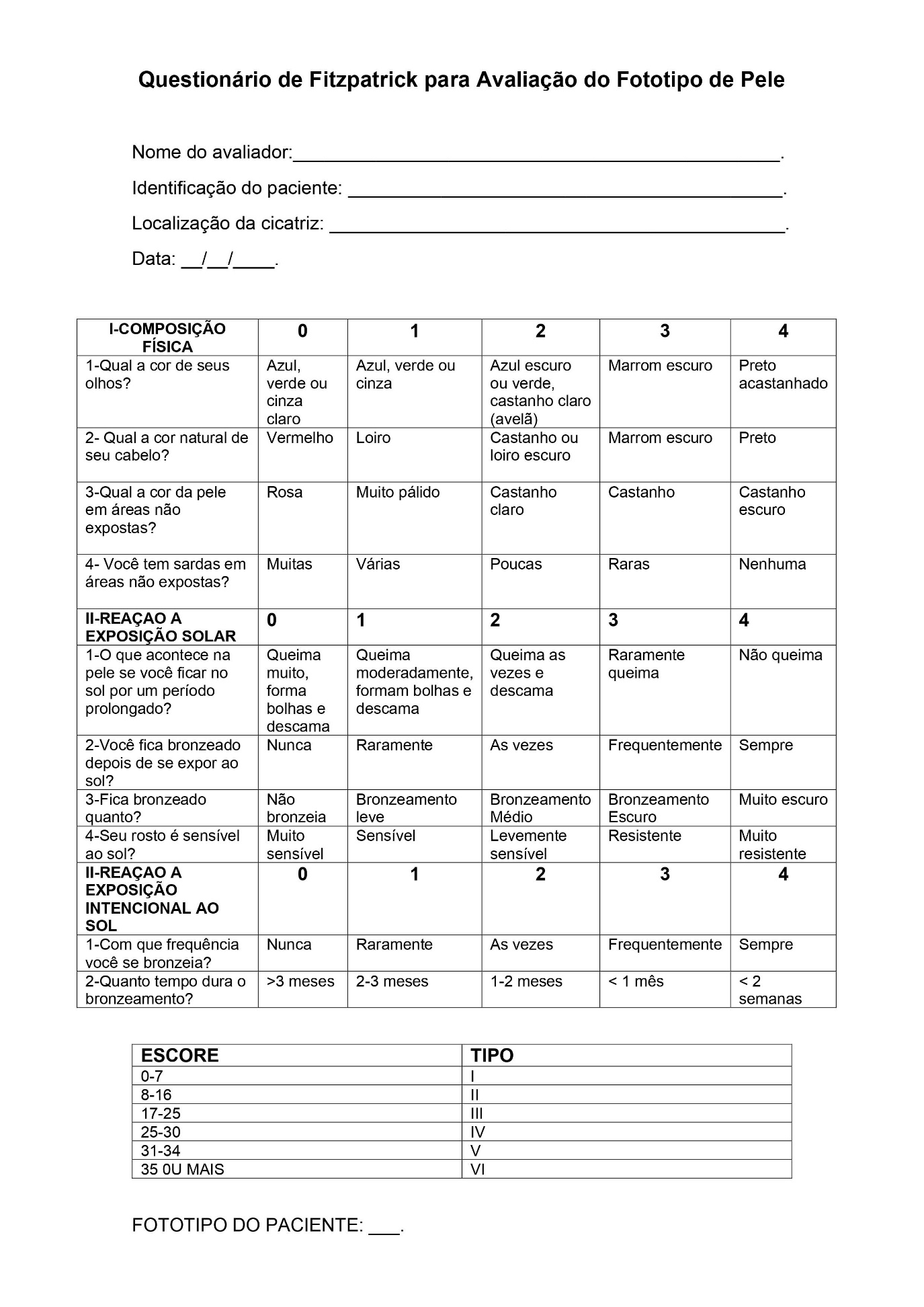


**
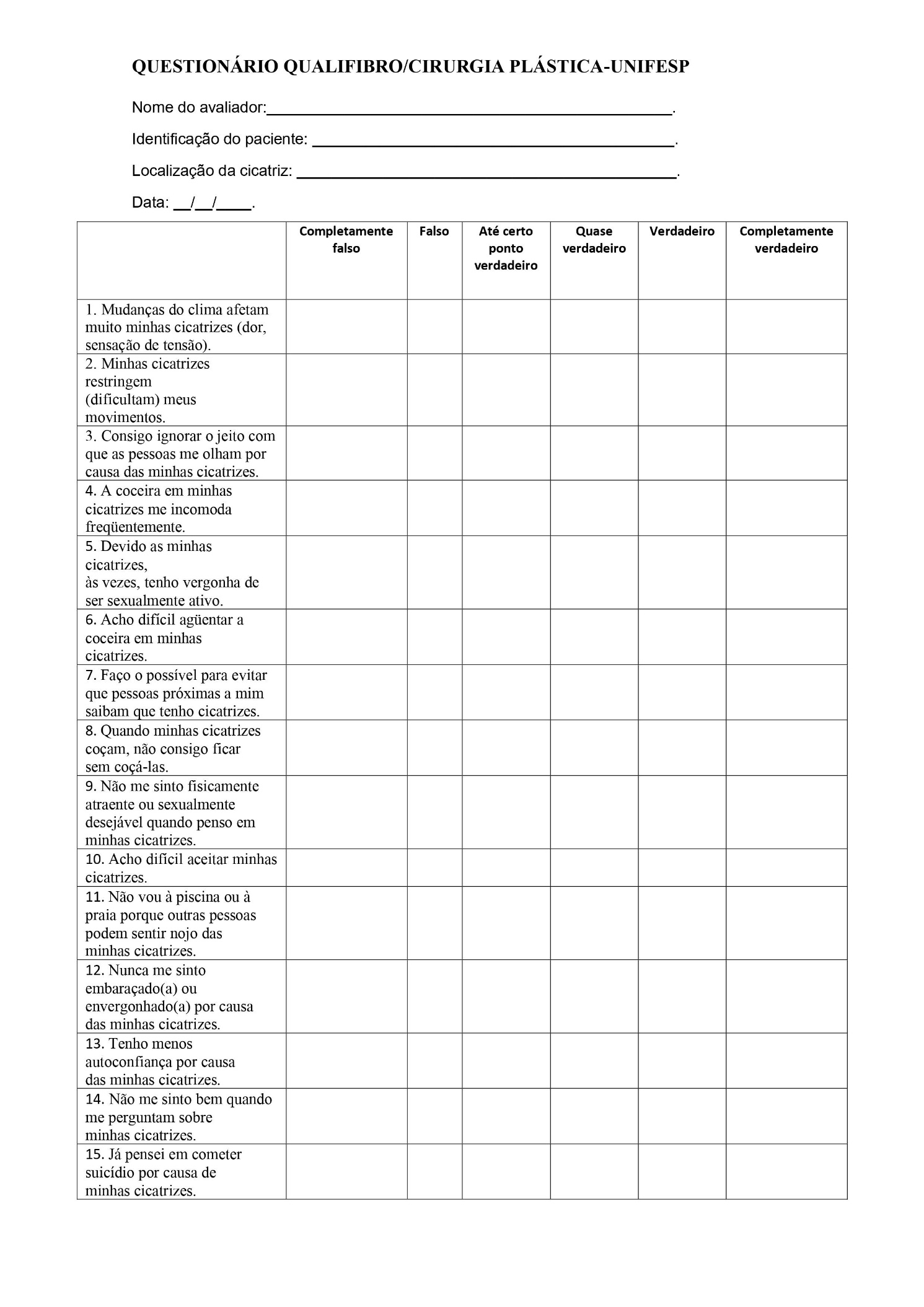
**12.2 ANEXO 2- QUESTIONÁRIO QUALIFIBRO-UNIFESP (FURTADO, 2008)

12.3 ANEXO 3- QUESTIONÁRIO PSAQ (OTA, 2016)

**
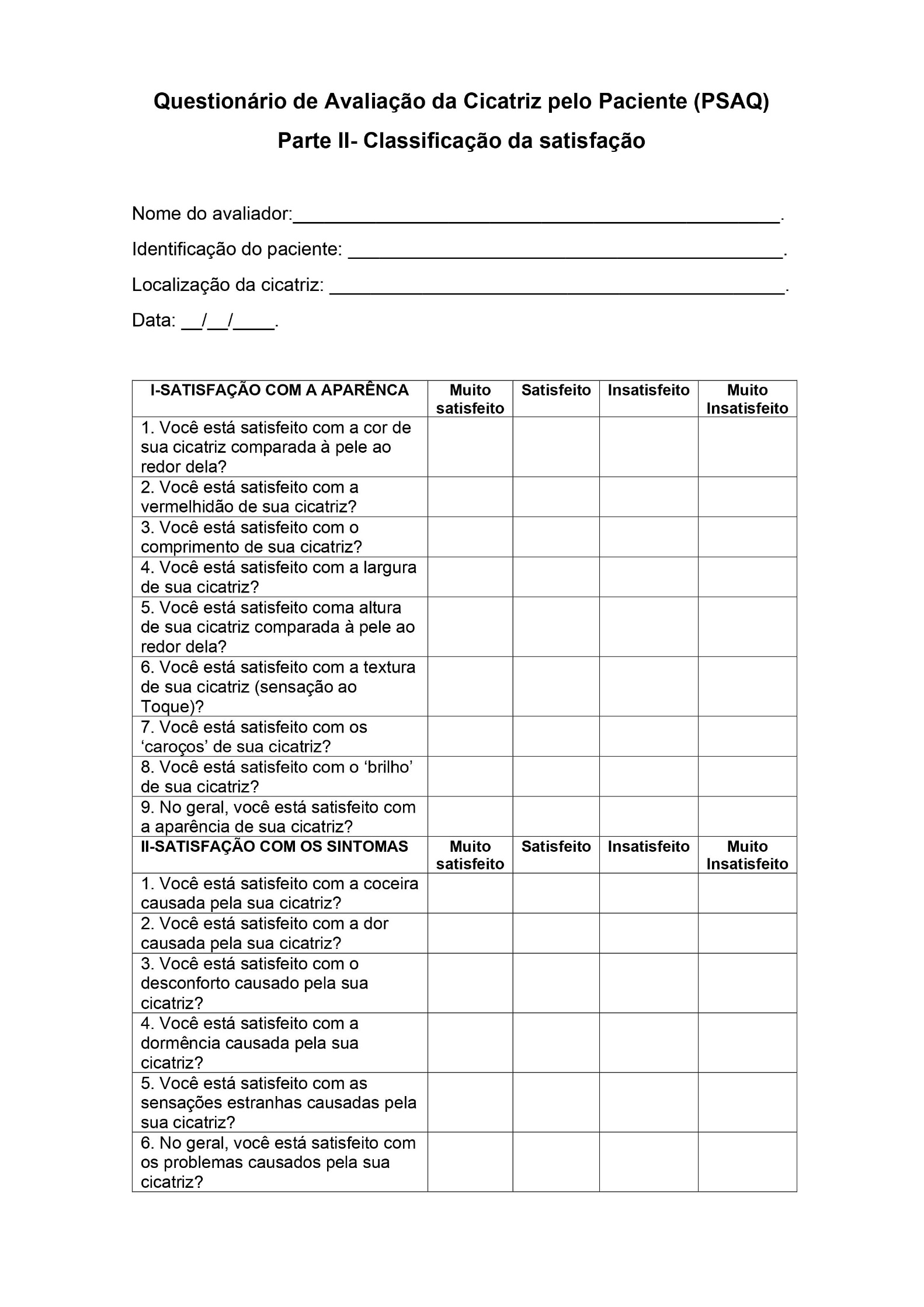
**

**
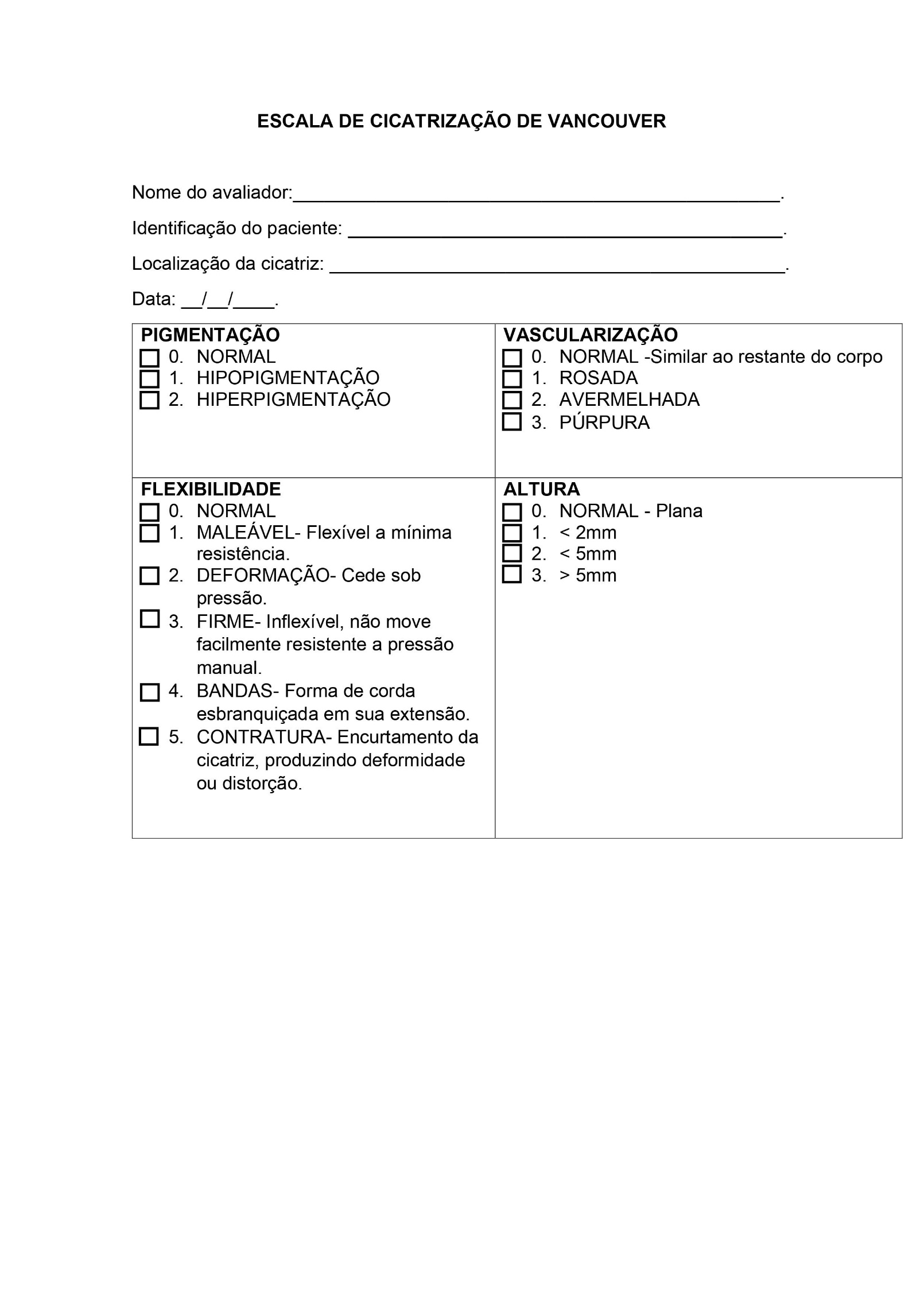
**12.4 ANEXO 4- QUESTIONÁRIO ESCALA DE CICATRIZ DE VANCOUVER (SANTOS, 2014)
