# Supplementary material for: Effect of the combination of photobiomodulation therapy and the intralesional administration of corticoid in the preoperative and postoperative periods of keloid surgery: A randomized, controlled, double-blind trial protocol study
Source: PLoS One. 2022 Feb 15;17(2):e0263453. doi: 10.1371/journal.pone.0263453 (PMC8846523; doi:10.1371/journal.pone.0263453)
Supplement: S2 File — (DOCX) [file pone.0263453.s002.docx]

UNIVERSITY NINTH OF JULY

STUDY OF THE EFFECT OF THE ASSOCIATION OF PHOTOBIOMODULATION THERAPY AND THE INTRALESIONAL APPLICATION OF CORTICOID IN THE PRE AND POST-OPERATIVE KELOID EXERSION: A CONTROLLED, RANDOMIZED AND DOUBLE-BLIND STUDY

JEFFERSON ANDRÉ PIRES

SAO PAULO

2020

**JEFFERSON ANDRÉ PIRES**

RESEARCH PROJECT

STUDY OF THE EFFECT OF THE ASSOCIATION OF PHOTOBIOMODULATION THERAPY AND THE INTRALESIONAL APPLICATION OF CORITCOID IN THE PRE AND POST-OPERATIVE KELOID EXERSION: A CONTROLLED, RANDOMIZED AND DOUBLE-BLIND STUDY

**Advisor: Prof. Dr. Raquel Agnelli Mesquita Ferrari**

**Sao Paulo**

**2020**

**ABSTRACT**

Keloid-type scars are characterized by the excessive proliferation of fibroblasts and the break in the balance between collagen production and degradation, with its increase in the dermis. The genesis of this pathology is not fully elucidated, in addition to the genetic aspects, it is also known that it is related to the increased expression of TGF-β. There is still no defined gold standard treatment, and relapse is present in all recommended. The most studied treatment is the intralesional application of corticosteroids alone or in association in the pre- and postoperative period of keloid removal. Because of this, new treatment alternatives must be sought. Photobiomodulation (FBM) with blue light has been shown in in vitro studies to decrease the multiplication rate and the amount of fibroblasts as well as TGF-β. It is a low-cost, non-invasive and without side effects therapy, proving to be a good tool to associate with the most recommended treatment. Thus, the aim of this study is to evaluate the effect of blue light associated with treatment with corticosteroids in the pre- and postoperative period of keloid excision. A randomized, controlled and double-blind clinical trial will be carried out, divided into two groups: 1) Sham (N=29): intralesional application of corticosteroids (AIC) in the pre- and postoperative period of keloid excision and 2) FBM associated with AIC ( N=29) in the pre- and postoperative period of keloid exeresis. FBM will be performed in a punctual transcutaneous way on the keloid in the preoperative period and on the scar remaining in the postoperative period using a blue LED pen (470nm, 400mW, 6.6mJ per point, with 10 linear points). Patients will answer two questionnaires, one to assess quality of life (Qualifibro-UNIFESP), and one to assess scar satisfaction (PSAQ) and the team of plastic surgeons will complete the Vancouver questionnaire for scar assessment (VSS), all will be answered with 01, 03 , 06 and 12 months. Keloids will be molded at the beginning of the silicone treatment and before resection to assess the size of the pre- and post-treatment area and similarly the remaining scar at 01, 03, 06 and 12 months after surgery. The removed keloid will be sent for histopathological analysis including the amount of fibroblasts and the organization and distribution of collagen (picrosirius staining), and TGF-β. All data will be subjected to statistical analysis. and one for evaluation of scar satisfaction (PSAQ) and the team of plastic surgeons will complete the Vancouver questionnaire for scar evaluation (VSS), all will be answered at 01, 03, 06 and 12 months. Keloids will be molded at the beginning of the silicone treatment and before resection to assess the size of the pre- and post-treatment area and similarly the remaining scar at 01, 03, 06 and 12 months after surgery. The removed keloid will be sent for histopathological analysis including the amount of fibroblasts and the organization and distribution of collagen (picrosirius staining), and TGF-β. All data will be subjected to statistical analysis. and one for evaluation of scar satisfaction (PSAQ) and the team of plastic surgeons will complete the Vancouver questionnaire for scar evaluation (VSS), all will be answered at 01, 03, 06 and 12 months. Keloids will be molded at the beginning of the silicone treatment and before resection to assess the size of the pre- and post-treatment area and similarly the remaining scar at 01, 03, 06 and 12 months after surgery. The removed keloid will be sent for histopathological analysis including the amount of fibroblasts and the organization and distribution of collagen (picrosirius staining), and TGF-β. All data will be subjected to statistical analysis. Keloids will be molded at the beginning of the silicone treatment and before resection to assess the size of the pre- and post-treatment area and similarly the remaining scar at 01, 03, 06 and 12 months after surgery. The removed keloid will be sent for histopathological analysis including the amount of fibroblasts and the organization and distribution of collagen (picrosirius staining), and TGF-β. All data will be subjected to statistical analysis. Keloids will be molded at the beginning of the silicone treatment and before resection to assess the size of the pre- and post-treatment area and similarly the remaining scar at 01, 03, 06 and 12 months after surgery. The removed keloid will be sent for histopathological analysis including the amount of fibroblasts and the organization and distribution of collagen (picrosirius staining), and TGF-β. All data will be subjected to statistical analysis.

**Key words:**Keloids, Photobiomodulation, corticotherapy, surgical treatment.

SUMMARY

[1. INTRODUCTION 1](#_Toc59456879)

[2 JUSTIFICATIONS 9](#_Toc59456880)

[3 OBJECTIVES 10](#_Toc59456881)

[4 HYPOTHESES 10](#_Toc59456882)

[5 MATERIAL AND METHODS 10](#_Toc59456883)

[5.1 RESEARCH OUTLINE 10](#_Toc59456884)

[5.2 SAMPLING AND RANDOMIZING 11](#_Toc59456885)

[5.2.1 Inclusion criteria 11](#_Toc59456886)

[5.2.2 Exclusion Criteria 11](#_Toc59456887)

[5.2.3 Composition of groups 12](#_Toc59456888)

[5.2.4 Calculation and sample size 12](#_Toc59456889)

[5.2.5 Randomization 13](#_Toc59456890)

[5.3 PRE-TREATMENT EVALUATION 14](#_Toc59456891)

[5.3.1 Questionnaires 14](#_Toc59456892)

[5.3.2 Measurement of the hangover scar 14](#_Toc59456893)

[5.4 PRE-OPERATIVE PROCEDURES 14](#_Toc59456894)

[5.4.1 Intralesional application of corticosteroids 14](#_Toc59456895)

[5.4.2 Application of photobiomodulation 15](#_Toc59456896)

[5.5 SURGICAL PROCEDURE 15](#_Toc59456897)

[5.5.1 Application of local anesthesia 16](#_Toc59456898)

[5.5.2 Operative act 16](#_Toc59456899)

[5.5.2.1 Exeresis procedure 16](#_Toc59456900)

[5.5.2.2 Hemostasis procedure 16](#_Toc59456901)

[5.5.2.3 Skin Synthesis Procedure 16](#_Toc59456902)

[5.5.2.4 Dressing 17](#_Toc59456903)

[5.6 POST-OPERATIVE PROCEDURES 17](#_Toc59456904)

[5.6.1 Intralesional application of corticosteroids 17](#_Toc59456905)

[5.6.2 Application of photobiomodulation 17](#_Toc59456906)

[5.6.3 Questionnaires 18](#_Toc59456907)

[5.7 HISTOPATHOLOGICAL ANALYSIS 18](#_Toc59456908)

[5.7.1 Fibroblast analysis 19](#_Toc59456909)

[5.7.2 Collagen analysis 19](#_Toc59456910)

[5.7.3 TGF-β Analysis 19](#_Toc59456911)

[5.7.3.1 Extraction and quality control of total RNA 19](#_Toc59456912)

[5.7.3.2 Synthesis of complementary DNA (CNA) and quantitative real-time PCR (qPCR) 19](#_Toc59456913)

[5.7.3.3 analysis of protein expression by ELISA (enzyme-linked immunosorbent assay) 20](#_Toc59456914)

[5.7.3.4 Statistical analysis 21](#_Toc59456915)

[6 OBTAINING PHOTOGRAPHS 21](#_Toc59456916)

[7 OUTCOMES 22](#_Toc59456917)

[8 SCHEDULE 22](#_Toc59456918)

[9 BUDGET 23](#_Toc59456919)

[10 BIBLIOGRAPHIC REFERENCES 24](#_Toc59456920)

[11 APPENDICES 33](#_Toc59456921)

[11.1 APPENDIX 1- TERM OF FREE AND INFORMED CONSENT 33](#_Toc59456922)

[11.2 APPENDIX2- DOCUMENT FOR STANDARDIZATION OF SURGERY. 36](#_Toc59456923)

[11.3 APPENDIX 3- TERM OF CONFIDENTIALITY 37](#_Toc59456924)

[11.4 APPENDIX 4- LETTER OF CONSENT 38](#_Toc59456925)

[12 ATTACHMENTS 39](#_Toc59456926)

[12.1 ANNEX 1- SKIN PHOTOTYPE CLASSIFICATION (FITZPATRICK, 1988) 39](#_Toc59456927)

[12.2 ANNEX 2- QUALIFIBRO-UNIFESP QUESTIONNAIRE (FURTADO, 2008) 40](#_Toc59456928)

[12.3 ANNEX 3- PSAQ QUESTIONNAIRE (OTA, 2016) 41](#_Toc59456929)

[12.4 ANNEX 4- VANCOUVER SCAR SCALE QUESTIONNAIRE (SANTOS, 2014) 42](#_Toc59456930)

1. INTRODUCTION

The healing process is a dynamic, complex and coordinated event involving several feedback loops and regulatory circuits of molecular, cellular and biochemical events with the aim of regeneration of the injured tissue (CAMPOS et al, 2007). This process can be divided into three main phases: inflammatory (hemostasis and inflammation), proliferative (granulation tissue) and remodeling (maturation) (CLARK, 2005) these phases overlap and are mediated by several factors, such as interleukins, growth factors , cytokines, vitamins, among others (NELIGAN et al, 2016; FERREIRA et al, 2007;POTTER, VEITCH AND JOHNSTON, 2019). Table 1.

| **PHASE** | **DURATION** | **MAIN FACTORS AND CELLS INVOLVED** | **MAIN EVENTS** |
| --- | --- | --- | --- |
| **INFLAMMATORY** | 48h-72h | Cells: Platelets, neutrophils and macrophages.  Factors:  TGF-β, thromboxane A2, prostraglandins, PDGF, FGF, EGF, | Activation of the coagulation cascade; vasodilation, cell chemotaxis, bacterial destruction, onset of fibroplasia and ECM formation. |
| **PROLIFERATIVE** | 4 to 25 days | Fibroblasts, VEGF, PDGF, myofibroblasts, keratinocytes, TGF-β, TNF-α. | Epithelialization, neoangiogenesis, formation of granulation tissue and collagen deposition (type III first and I later, wound contraction. |
| **RENOVATION** | months to years | Type I and III collagens, collagenases. | Collagen organization, progressive replacement from type III to type I. |

**Table 1-**Healing phases: Main factors and events involved. Caption: TGF-β= Transforming factor beta; PDGF= platelet-derived growth factor; FGF=fibroblast-derived growth factor; EGF=epidermal growth factor; VEGF = vascular endothelial growth factor; TNF-α= tumor necrosis factor alpha; ECM= extra-cellular matrix.

During the healing phases, any morbidity or event that disrupts the healing process can inhibit or harm the entire process, causing no closure of the lesion, such as in ulcers, or on the contrary, the exacerbation of these events can induce hyperproliferative scars such as hypertrophics and keloids (NELIGAN et al, 2016; FERREIRA et al, 2007;POTTER, VEITCH AND JOHNSTON, 2019). Table 2.

| **FACTORS THAT INTERFERE WITH HEALING** | **MAIN EXAMPLES** |
| --- | --- |
| **PLACES** | Infections; ischemia, presence of a foreign body; improper handling, excessive pressure on the scar, irradiation. |
| **SYSTEMIC** | Protein depletion, vitamin A, C and B complex deficiency; deficiency of trace elements such as zinc; Diabetes Mellitus, obesity; chemotherapies and corticosteroids. |
| **ASSOCIATED SYNDROMES** | Ehlers-Danlos; homocystenuria; osteogenisis imperfecta. |
| **ETHNIC PREDICTION** | Africans and Asians (keloids). |

Table 2- Factors that interfere with healing and their main examples. (NELIGAN et al, 2016; FERREIRA et al, 2007).

With regard to excessive pathological scars, hypertrophic and keloid scars stand out. The first is mainly related to poor surgical technique with inadequate tissue synthesis and areas of greater tension for closing the skin, such as the anterior chest and dorsal region. It is restricted to the scar line and usually regress spontaneously (NELIGAN et al, 2016; FERREIRA et al, 2007; MAHDAVIAN et al, 2012;WOLFRAM, 2009, LEE PENG AND KEROLUS, 2019).

Excessive keloid-type scars are characterized by the excessive proliferation of fibroblasts and the disruption of the balance between collagen production and degradation, with its increase in the dermis. Unlike hypertrophic scars, keloids do not regress spontaneously, they go beyond the scar line with disorganized, misshapen and coarse growth, compared to a benign tumor, including this nomina derives from the Greek "kelth" which means tumor and eidoz which means shape (FERREIRA et al, 2007; WOLFRAM et al, 2009,LIMANDJAJA, NIESSEN, SCHEPER AND GIBBS, 2020, NANGOLE AND AGAK, 2019).

In addition to the unpleasant aesthetic aspect, these scars are often painful, itchy and can cause functional incapacity (for example, scars on the joints), resulting in psychosocial damage and in the patients' quality of life. (BOCK et al, 2006; MOTOKI et al, 2018).

Histopathological features of keloids on light microscopy include thin epithelial tissue, thickened dermis, increased collagen fibers that are disorganized and interspersed with abundant mucin and few elastic fibers. There is an increase in rounded fibroblasts and the sebaceous glands and hair follicles are diminished or absent. (FERREIRA et al, 2007; PLACIK AND LEWIS, 1992; ACKERMAN et al, 1997; KIKUCHI, KADONO AND TAKEHARA, 1995; BEER, 2008,LIMANDJAJA, NIESSEN, SCHEPER AND GIBBS, 2020).

Electron microscopy shows collagen fibers with reduced diameters compared to a normal scar, fibroblasts have actinomyosin in their cytoplasm, suggesting that many of these cells are a transition between fibroblasts and myofibroblasts. Histology also shows a predominance of type I collagen and a smaller amount of type III collagen, with type III being increased in keloids. (FERREIRA et al, 2007; REIS, 1994).

The genesis of this morbidity is not yet sufficiently understood, mainly due to the lack of in vivo studies and because this pathology has not been described in animals, thus hindering an adequate biological study. (WOLFRAN et al, 2009; ARNO et al, 2014;FERREIRA et al, 2007; NELIGAN et al, 2016).

Studies have shown that the genesis of fibroblast hyperproliferation is related to increased expression of growth and transforming factor β1 (TGF-β1) in endothelial cells of neovascularization tissue, which already presents increased VEGF expression and an increase in VEGF expression CTGF (Connective Tissue Growth Factor). (ARNO et al, 2014; LEE et al, 2017; HAHN et al, 2016; CHIN et al, 2001; FUJIWARA, MURAGAKI AND OOSHIMA, 2005; MARNEROS AND KRIEG, 2004; COLWELL, 2005; NANGOLE AND AGAK, 2019).

TGF-β plays a key role in the hyperproliferation of fibroblasts and consequently collagens. It regulates the proliferation of these cells as well as collagen synthesis. In a normal healing process, TGF-β levels decrease at the end of tissue repair, however in keloids it does not decrease, keeping its production high and unregulated. (WOLFRAM et al, 2009; SARRAZY, 2011; QU, 2012).

It has also been shown that in keloids there is a reduced amount of enzymes that degrade extracellular matrix components such as metalloproteinases. Increased PDGF (platelet-derived growth factor) receptors in fibroblasts, excessive activation of signals for insulin-like growth factor-1 (IGF-1), reduced number of fibroblast apoptosis and increased thymic stromal lymphopoietin expression (SHIN JU, KIM SH, KIM H, et al) and fibrocyte activation via stromal cell-derived factor-1 are other findings related to hyperproliferative scars. (HAISA, OKOCHI AND GROTENDORST, 1994; SAVAH et al 1999)

Some studies have shown that keloids may have their genesis predisposed by autosomal dominant genes with incomplete penetrance and varied expressions, however, there is no success in isolating a gene directly linked to the keloid. It has also been shown in some studies a higher prevalence in females (BROWN AND BAYAT, 2009; MARNEROS et al 2001; CHEN et al, 2006; CLARK et al, 2009; SHIH AND BAYAT, 2010; NAKASHIMA et al, 2010;NOISHIKI, HAYASAKA YE OGAWA, 2019).

There are several recommended treatments for keloids, however the literature is controversial since many of the studies conducted use questionable methodology and with inaccurate results, which makes it difficult to develop new treatment protocols. Therefore, there is no consensus on the best therapy to be used to reduce the risk of relapse and which is associated with fewer unwanted side effects which limit the use of many therapies. Tables 3a and 3b summarize the main treatments used described in the literature (ARNO et al, 2014; JAGADEESAN AND BAYAT, 2007; HAHN et al, 2016; GOLD, 2014; CHEN et al, 2020; GUPTA AND KALRA, 2002; DAVISON et al. al, 2009; VAN LEEUWEN, VAN LEEUWEN AND NIESSEN, 2014; VAN LEEUWEN et al, 2015; HAR-SHAI et al, 2007; JIN et al, 2013; VRIJMAN et al, 2011; VAN DROOGE et al, 2015; EROL et al. al, 2008; BETARBET AND BLALOCK, 2020; LEE et al, 2019).

Treatments are mainly aimed at reducing the patient's symptoms (pain, itching and functional limitations), reducing scar volume and improving aesthetic quality.

| **MAIN MONOTHERAPIES** | **SIDE EFFECTS** | **recidivism** |
| --- | --- | --- |
| **SURGICAL RESECTION** | Minimal complications related to the surgical procedure. | 45-100% |
| **INTRALESIONAL CORTICOID APPLICATION**  **(TRIANCINOLONE)** | ulcerations, changes in skin color, appearance of telangiectasias, reports of Cushing's syndrome. | <50% |
| **5-FLUORACIL** | Pain, hyperpigmentation, ulcerations. Report of transient alopecia. | Similar to triancil, few studies with isolated use, usually associated with other therapies. |
| **CRYOTHERAPY** | Permanent hypopigmentation of the skin, pain, limited use for patients with larger phototypes. | 24% (around 50% respond positively to isolated treatment) |
| **LASERS**  **(ablative)** | Erythema, altered skin pigmentation, scarring after ulcer formation. | 74-100% used alone. |

**Table 3a-** Main monotherapies used to treat keloids (ARNO et al, 2014; JAGADEESAN AND BAYAT, 2007; HAHN et al, 2016; SHIN et al, 2016; SHIN AND KIM, 2016; GOLD, 2014; CHEN et al, 2020; GUPTA AND KALRA, 2002; DAVISON et al, 2009; VAN LEEUWEN, VAN LEEUWEN AND NIESSEN, 2014; VAN LEEUWEN et al, 2015; HAR-SHAI et al, 2007; JIN et al, 2013; VRIJMAN et al, 2011; VAN DROOGE et al, 2015; EROL et al, 2008; BETARBET AND BLALOCK, 2020; LEE et al, 2019).

| **MAIN COMBINED THERAPIES** | **SIDE EFFECTS** | **recidivism** |
| --- | --- | --- |
| **SURGICAL EXERESIS + CORTICOID INJECTION** | The same related to the application of corticoids only, depressions in the scar, wound dehiscence. | <30% |
| **SURGICAL EXERESIS + RADIOTHERAPY** | Described malignant diseases. | Around 22%, it varies greatly between studies. |

**Table 3b-** Main combined therapies used to treat keloids (ARNO et al, 2014; JAGADEESAN AND BAYAT, 2007; HAHN et al, 2016; SHIN et al, 2016; SHIN AND KIM, 2016; GOLD, 2014; CHEN et al, 2020; GUPTA AND KALRA, 2002; DAVISON et al, 2009; VAN LEEUWEN, VAN LEEUWEN AND NIESSEN, 2014; VAN LEEUWEN et al, 2015; HAR-SHAI et al, 2007; JIN et al, 2013; VRIJMAN et al, 2011; VAN DROOGE et al, 2015; EROL et al, 2008; BETARBET AND BLALOCK, 2020; LEE et al, 2019).

Among the treatments mentioned, we highlight the resection of the lesion with a margin, the use of injectable corticosteroids and radiotherapy, in addition to the association of therapies, with pre- and postoperative application. Studies demonstrate the remission of keloids, however many of them show a high degree of disease recurrence using all the treatments already recommended (ARNO et al, 2014; WOLFRAM, 2009; HUU et al, 2019; SHIN et al, 2019; CHEN et al , 2019).

Surgical resection with a margin alone does not have good results, demonstrating in studies up to 45% to 100% of recurrence in the postoperative period (BERMAN, MADERAL AND RAPHAEL, 2017).These high recurrences have been studied and their main cause would be resection with inadequate margins, as studies show a high amount of fibroblasts in activity in the periphery of the keloids, in addition to tension in the closure of the lesion, lack of care in handling the tissues and poor technique in the performing hemostasis. Even with all care, recurrence is high, requiring adjuvant therapies in the pre- and/or postoperative periods. (ARNO et al, 2014; WOLFRAM, 2009; SYED et al, 2012; TAN et al, 2010;CHEN et al, 2020; MOHAMMADI, 2019).

Radiotherapy has been shown to be effective in reducing keloid recurrence used after surgical excision, compared to its use alone (MANKOWSKI et al, 2017), however there is no accepted consensus on the dose or modality to be used in postoperative treatment . Furthermore, the potential long-term risks and the anatomical site where the radiation will be carried out, associated with the high cost, limit its use (OGAWA et al, 2009). Reports of malignant disease after radiation on keloids have already been reported in the literature. (ARNO et al, 2014; WOLFRAM, 2009; DE CICCO et al, 2014; OGAWA et al, 2009).

The intralesional application of triamcinolone hexacetonide (Triancil®- Registered trademark of Apsen Farmacêutica SA), it has been considered the first-line treatment for keloids, the recommended dose is 2.5mg to 20mg in the topography of the face and 20mg to 40mg in other regions of the body. sThe mechanism is based on the decrease in collagen and glycosaminoglycan synthesis and on the inhibition of fibroblast production (SHIN JY et al, 2016). Due to its anti-inflammatory and vasoconstrictive effect, it is also observed a decrease in itching and pain in patients. Studies show variable recurrence rates up to 50% (ARNO et al, 2014; GOLD, 2014; LEDON et al, 2013; HUU et al, 2019; CHEN et al, 2019).

There is no consensus on the amount and time of treatment with this corticoid, and prolonged use can lead to ulcerations, changes in skin color and the appearance of atelangectasis (ARNO et al, 2014) in addition to reports of the appearance of Cushing's syndrome after its use (SHIN JY et al, 2016; GOLD, 2014; LEDON et al, 2013; HUU et al, 2019; CHEN et al, 2019).

The isolated use of corticosteroids, in addition to the side effects already mentioned, often does not induce the total regression of keloids and result in non-esthetic residual scars, with color change, enlargement, telangiectasias and depression. (GOLD, 2014; LEDON et al, 2013)

Combined therapy of surgical resection with the use of injectable corticosteroids has been shown to be cost-effective and safe, with a decrease in relapse rates, around 29%, despite a wide range of protocols and results, which makes accurate assessment difficult. (SCLAFANI et al, 1996; HAMRICK, BOSWELL AND CARNEY, 2009; SHONS AND PRESS, 1983; BERMAM AND FLORES, 1997; KIIL, 1977; JUNG et al, 2009).

Due to the high incidence of recurrence with the use of current treatments, the keloid can remain for a long time being often incapacitating, with reduced quality of life (MOTOKI et al, 2018). This fact leads to the constant search for new minimally invasive products and technologies in order to improve the treatment, with fewer side effects and a lower rate of recurrence.

In this context, photobiomodulation therapy (FBM) has gained increasing attention in the literature. FBM also known as low intensity light therapy uses low power light sources, usually below 500mW, with non-ionizing irradiation in both the visible range of the electromagnetic spectrum (400-760nm) and infrared (760-1000nm ), which act on tissues producing a positive biological effect on cells (FERNANDES, FERRARI & FRANÇA, 2017; FREITAS & HAMBLIM, 2016).

Photobiological responses are due to chemical and physical changes induced by photons in biological tissues. Each wavelength will interact more specifically with a specific chromophore (photoreceptor molecule) and this interaction will initiate a chain of photochemical and photophysical reactions. An important chromophore for red and near infrared wavelengths is the enzyme cytochrome C oxidase, an enzyme found in the mitochondria and which participates in the electron transport chain, signaling pathways that induce the production of nitric oxide (NO), Adenosine triphosphate (ATP) and reactive oxygen species (ROS), which can induce the transcription of various growth factors such as platelet-derived (PDGF), TGF-β, fibroblast-derived (FGF), interleukins and tumor necrosis factor alpha (TNF-α) ,

There are studies using FBM in postoperative scars from different types of surgery demonstrating the beneficial effects of FBM in the healing process, thus showing the effectiveness and safety of this therapy. (BAROLET AND BOUCHER, 2010; OJEA et al, 2016; FREITAS et al, 2013; ALSHARNOUBI et al, 2018; EPSTEIN et al, 2018; HERASCU et al, 2005; RAMOS et al, 2019; CARVALHO et al, 2010; FUJI et al, 2008; PARK et al, 2016).

In vitro experimental studies using fibroblasts from keloids and human dermis have shown positive effects including an increase in the rate of apoptosis and a decrease in the rate of cell division of these fibroblasts, decreased collagen synthesis and GFR-β expression. In these studies, blue light with a wavelength ranging from 410-480nm is highlighted and there is an inhibitory effect on fibroblasts and on TGF-β with higher energy densities (0.326 TO 640J/cm²). (LEE et al, 2017; HAHN et al, 2019; MAMALIS et al, 2016; MIGNON et al, 2018; OPLÄNDER et al, 2011;HAWKINS AND ABRAHAMSE, 2006; LEV-TOV, BRODY, SIEGEL AND JAGDEO, 2013; FRIGO et al, 2010; BONATTI et al, 2011).

It is possible that blue light interacts with mitochondrial chromophores in the same way as red and infrared light, since the heme centers that are scattered across the cytochromes have a significant absorption peak that coincides with the Soret band of porphyrins ( HAMBLIN MR, 2018). In the blue light spectrum, flavoproteins such as NADH-dehydrogenase and succinate-dehydrogenase, and porphyrins can function as photoreceptors (KARU TI,KOLYAKOV SF, 2005).

These studies, associated with the results of the beneficial effects of FBM in the healing process, suggest a promising way for the establishment of this therapeutic modality in the treatment of keloids.

2 JUSTIFICATIONS

Pathological scars, especially those of the keloid type. They are stigmatizing, grow uncontrolled and irregular, itchy and, in many cases, painful, thus, often incapacitating and limiting the daily activities and social life of those affected by this disease.

The currently most used treatments for keloids are related to undesirable side effects, high recurrence (trying to standardize the term) after treatment and, in our country, the high costs that make it impossible for many patients to have a complete treatment. Even with several studies in the literature, there is no consensus on the best treatment or protocol to be adopted in the case of keloids, mainly due to methodological design flaws, incomplete description of the results and analysis performed, as well as uncertainties about the keloid, which include unpredictable pathology and causes still poorly defined.

The FBM therapy with LED or LLLT comes with a therapeutic possibility due to its effects on the healing and repair process, being a low-cost, non-invasive, without side effects, easy to use and reproducible alternative. More specifically, FBM has already been shown to positively modulate the inflammatory process, specifically TNF-β and type I collagen, which are associated with keloid genesis. Thus, the objective is to associate FBM in the pre- and postoperative period of surgical resection in order to improve the healing process and scar quality, reducing the possibility of recurrence.

3 OBJECTIVES

The main objective of this double-blind randomized clinical trial is to verify the effects of the combination of FBM and the application of intralesional corticosteroids on the rate of keloid recurrence after surgical resection and on the quality of the newly formed scar.

The secondary objectives are to evaluate the effects of FBM applied prior to resection on the quantity and organization of fibroblasts and collagens, on the gene expression of TGF-β and on the quality of life of the participants.

4 HYPOTHESES

The FBM protocol in combination with corticosteroid therapy before and after keloid exeresis improves the distribution and organization of collagen in the scar tissue, decreases the expression of TGF-β, reducing the risk of recurrence and thus improves the general appearance of the scar and improves the participant's quality of life.

5 MATERIAL AND METHODS

5.1 RESEARCH OUTLINE

A randomized, double-blind (patient, team and analyst) controlled clinical trial will be conducted, from December 2020 to March 2023, in patients with keloids treated at the Plastic Surgery Service of the Conjunto Hospitalar do Mandaqui and also recruited from the Specialties Clinic of the Nove de Julho University (UNINOVE) in the city of São Paulo-SP. This clinical trial was written in accordance with the Declaration of Helsinki (revised in Fortaleza, 2014) and will be submitted to the Ethics and Research Committee of Universidade Nove de Julho (Uninove) and Conjunto Hospitalar do Mandaqui (CHM).

After verbal and written explanation of the study, participants who accept the invitation to participate in the research will sign the Free and Informed Consent Term (FICF) (Appendix 1).

5.2 SAMPLING AND RANDOMIZING

Volunteers of both sexes aged between 18 and 65 will be selected from the Specialty Outpatient Clinic of the Conjunto Hospitalar do Mandaqui and the Nove de Julho University, in the city of São Paulo, who have keloids and are eager for treatment.

5.2.1 Inclusion criteria

People between 18 and 65 years old, with Fitzpatrick I-VI skin phototype, presenting keloids without any previous treatment, or in relapses of surgical excision only or relapse after using other therapies with at least 03 months without being included, will be included in the research. using any treatment, seen at the Plastic Surgery Service of the Conjunto Hospitalar do Mandaqui, in the city of São Paulo-SP.

5.2.2 Exclusion Criteria

Patients outside the studied age group will be excluded from the study, as well as those with keloids with any type of treatment in force, pregnant patients and keloids who do not have the possibility of resection with primary skin synthesis.

5.2.3 Composition of groups

Patients will be randomized into two groups: A) STUDY: Group ACI + FBM + ECQ: Photobiomodulation (FBM) and application of corticosteroids in the pre- and postoperative period of surgical keloid excision (ECQ).

**B) CONTROL (SHAM):** ACI + ECQ Group: Application of injectable corticosteroids (ACI) in the pre- and postoperative period of surgical keloid excision (ECQ). The application of FBM will be simulated with a device identical to the one in the study group, with a pen with the same light color, however without power and consequently without effect.

5.2.4 Calculation and sample size

Sample calculation was performed to show a difference in means between two groups: group1: patients using corticosteroids and group2: patients using corticosteroids and photobiomodulation in chieloids.

Using a significance level equal to 0.05 and a power of 80%, and based on the article by HEWEDY et al (2020) to obtain the averages of improvement in post-surgical healing, in 3 months, by the Vancouver scar scale (group corticoid: mean=1.95; SD=1.84). Due to the lack of data in the literature regarding group 2, we estimated the same variability found in group 1. The sample size being defined as shown in table 4.

| No 10% loss | |  | With 10% loss | |  |  |
| --- | --- | --- | --- | --- | --- | --- |
| Average G1 vs Average G2  (3 months post) | Difference | nTotal | nGroup |  | nTotal | nGroup |
| 1.95 vs 0.25 | 1.7 | 38 | 19 |  | 58 | 29 |

**Table 4-** Sample size with and without 10% loss.

5.2.4.1 Sample calculation methodology

Categorical variables will be represented by absolute and relative frequency. Quantitative variables will be represented by mean and standard deviation or median and interquartile range (median [P25; P75]) according to the distribution verified by the Shapiro-Wilk normality test. When one of the groups obtained size (n) smaller than 12 subjects, the quantitative variables will be directly represented by median and interquartile range and compared by non-parametric tests.

The variables in the pre-time will be compared as follows: the proportions of the studied variables will be compared between the groups using the chi-square test. Comparisons of means of quantitative variables between groups will be performed using the t test for independent samples or the Mann-Whitney test.

To compare the groups and the times of the means of the studied quantitative variables (for example: Vancouver score, healing area, etc.) the Generalized Estimation Equations (GEE Model) model will be performed (LIANG AND ZEGER, 1986; ZEGER E LIANG, 1986; GUIMARÃES AND HIRAKATA, 2012). This analysis will be performed since the samples are related, that is, the same patient responds to the same measurements at different times. The main effect of group, time and the double interaction of factors (group*time) will be tested. The model will consist of an unstructured working correlation matrix, a robust estimator covariance matrix and a distribution according to the normality test result (normal or gamma) with an adequate link function (identity or logarithmic).

Analyzes will be performed using IBM SPSS Statistics v.25 software. The level of significance adopted will be 0.05. Graphical analyzes will be performed in Excel® 2013.

5.2.5 Randomization

A block randomization will be performed using an undetermined sequence generator program (Reserch Randomizer, version 4.0 [computer software], available since June 2013 in<http://www.randomizer.org>).

5.3 PRE-TREATMENT EVALUATION

5.3.1 Questionnaires

Three questionnaires will be applied to participants containing identification information and a questionnaire to experts after clinical evaluation. The first one will be for the classification of the skin phototype through the Fitzpatrick classification (FITZPATRICK, 1988) (Appendix 1).

Second, the Quality of Life of Patients With Keloid And Hypertrophic Scarring questionnaire (BOCK et al., 2006) will be applied, which was translated and validated to Portuguese and named QualiFibro/Plastic Surgery-UNIFESP questionnaire (FURTADO, 2008) (Appendix 2 ).

Finally, the patient will answer part II (satisfaction rating) of the Patient Scar Assessment Questionnaire (PSAQ) (DURANI, MCGROUTHER, FERGUSON, 2009), which was translated and validated into Portuguese and called the patient's scar assessment questionnaire. (OTA, 2016) (Annex 3).

The Vancouver Scar Scale (VSS) questionnaire, published by Sullivan et al (1990) and translated by Santos et al (2014) (Annex 4).

5.3.2 Measurement of the hangover scar**The**

Scar molds will be obtained with light condensation silicone (Zhermack, Badia Polesine, Italy). The material will be placed in acrylic plates and sent for analysis by optical coherence tomography, obtaining the area and volume. The procedure will be repeated after preoperative therapy and at 03, 06 and 12 months after surgery.

5.4 PRE-OPERATIVE PROCEDURES

5.4.1 Intralesional application of corticosteroids

Participants in the study and control groups (Sham) will undergo the application of the corticosteroid triamcinolone hexacetonide (Triancil®- Apsen Farmacêutica SA), with two applications at two-week intervals before the keloid exeresis surgery. The applications will be performed with syringes for applying 1ml insulin with a fixed needle, intralesional without going beyond the dermis.

The medication used has a presentation of 20mg/ml, it will be diluted with the same amount of 2% lidocaine. The scar will be divinity in equal parts of 1cm² and distributed equally, respecting the total doses per session of 20mg for the face and 40mg for the other topographies.

5.4.2 Application of photobiomodulation

Photobiomodulation will be performed in four preoperative sessions at two-week intervals using the blue LED light source (Laser therapy system, Quantum model, Ecco® brand) according to the dosimetric parameters listed in table X.

| **DOSIMETRIC PARAMETERS** | **SOURCE TYPE: LED** |
| --- | --- |
| Central wavelength [nm] | 470 |
| Operation mode | Continuous |
| Average radiant power [mW] | 400 |
| Opening Diameter [cm] | 1.7 |
| Power Density at Aperture [W/cm2] | 0.17 |
| Beam area on target [cm2] | 2,268 |
| Target irradiance [W/cm2] | 0.4 |
| Duration of useful exposure [s] | Varies according to scar size, maximum 600. |
| Duration of exhibition [s] | 60 per point |
| Aperture energy density [J/cm2] | 105.8 considering maximum exposure |
| Radiant energy [mJ] | 240,000 considering maximum exposure |
| Energy per point [mJ] | 6.66 |
| application technique | Contact |
| Anatomical location of application points | On the remaining scar, 01 stitch per cm² |
| Number and frequency of treatment sessions | Weekly for 4 weeks. |

**Table 5-** Dosimetric parameters that will be used in the preoperative period.

5.5 SURGICAL PROCEDURE

All surgical procedures will be performed in a hospital operating room with all the care recommended for patient safety. A letter will be sent to the Plastic Surgery team of the Conjunto Hospitalar do Mandaqui with the standardization of the procedure for the study participants.

5.5.1 Application of local anesthesia

A local anesthetic solution at a concentration of 1:100,000 will be used, containing 20 ml of 2% lidocaine (20mg/ml) without vasoconstrictor, 20 ml of 0.5% bupivacaine hydrochloride (5ml/ml), 1ml of epinephrine ( 1mg/ml) and 60ml of saline solution (0.9%). The solution may undergo changes if it is necessary to adapt to the patient's weight, obeying the use of 5mg/kg of lidocaine and 3mg/km of bupivacaine.

After all care with antisepsis and asepsis, the solution will be applied with a 10ml sterile and disposable syringe with 13x45mm needles for surface and 25x7mm needles for depth. The amount of anesthetic will be proportionally divided along the entire length of the scar, with equal amounts being injected in areas of 1cm².

5.5.2 Operative act

All surgical procedures will be performed under the basic precepts of proper surgical technique, strictly observing the times of dieresis, hemostasis and synthesis.

5.5.2.1 Exeresis procedure

For keloid resection, nº 15 scalpel blades will be used, being resected in the form of a spindle or ellipse with 5mm margins, of healthy skin, measured after the end of the scar. As for depth, the entire scar should be resected until healthy tissue, free from fibrosis, is obtained.

5.5.2.2 Hemostasis procedure

Hemostasis should be performed with monopolar or bipolar electrocautery at low intensities (up to 30). It should be performed judiciously only where there is bleeding and if necessary to avoid unnecessary tissue damage.

5.5.2.3 Skin Synthesis Procedure

For the synthesis of the surgical wound, threads with less tissue reaction will be used. For subcutaneous cellular tissue, when necessary, absorbable suture based on polyglecaprone 25 (caprofyl®), with a 4-0 diameter and circular needle, will be used. Subdermal stitches, if necessary, should use nylon threads with a diameter of 4-0 to 6-0, where the thinner diameters (5-0 and 6-0) will be used in anatomical sites with less tension and thinner skin and larger diameter (4-0) in sites with greater tension and thicker skin.

For skin synthesis, intradermal suture will be used with 4-0 and 5-0 nylon thread, the first in areas with greater tension and thicker dermis and the second in anatomical sites without tension and with more dermis. slim.

5.5.2.4 Dressing

After the synthesis procedure, the scar will be cleaned with a 0.9% saline solution and dried. Afterwards, the microporing will be performed (placement of microporated tapes in “x” along the entire length of the scar (HOCHMAN B, ISHIZUKA CK, FERREIA LM, OLIVEIRA LQR, LOCALI RF, 2004).

5.6 POST-OPERATIVE PROCEDURES

5.6.1 Intralesional application of corticosteroids

Participants in both groups will undergo the application of the corticosteroid triamcinolone hexacetonide (Triancil®- Apsen Farmacêutica SA), with monthly applications with an interval of 30 days until 3 applications are completed. The applications will be performed with syringes for applying 1ml insulin with a fixed needle, intralesional without going beyond the dermis.

The medication used has a presentation of 20mg/ml, it will be diluted with the same amount of 2% lidocaine. The scar will be divinity in equal parts of 1cm² and distributed equally, respecting the total doses per session of 20mg for the face and 40mg for the other topographies.

5.6.2 Application of photobiomodulation

Photobiomodulation will be performed weekly in the first postoperative month, every 15 days in the second postoperative month and one application in the 3rd postoperative month, using the blue blue LED light source (Laser therapy system, model Quantum, Ecco® brand) according to the dosimetric parameters listed in table 6.

| **DOSIMETRIC PARAMETERS** | **SOURCE TYPE: LED** |
| --- | --- |
| Central wavelength [nm] | 470 |
| Operation mode | Continuous |
| Average radiant power [mW] | 400 |
| Opening Diameter [cm] | 1.7 |
| Power Density at Aperture [W/cm2] | 0.17 |
| Beam area on target [cm2] | 2,268 |
| Target irradiance [W/cm2] | 0.4 |
| Duration of useful exposure [s] | Varies according to scar size, maximum 600. |
| Duration of exhibition [s] | 60 per point |
| Aperture energy density [J/cm2] | 105.8 considering maximum exposure |
| Radiant energy [mJ] | 240,000 considering maximum exposure |
| Energy per point [mJ] | 6.66 |
| application technique | Contact |
| Anatomical location of application points | On the remaining scar, 01 stitch per cm² |
| Number and frequency of treatment sessions | Immediately after surgery completion, weekly for 4 weeks, every fifteen days for another 4 weeks and one application in the 3rd month. |

**Table 6-** Dosimetric parameters that will be used in the postoperative period.

5.6.3 Questionnaires

The patient will answer the QualiFibro/Plastic Surgery-UNIFESP questionnaire again (Appendix 2) and the PSAQ scar satisfaction part II (Appendix 3) at 01, 03, 06 and 12 months after surgery.

The trained and blinded specialists will answer the VSS (Appendix 4) again at 01, 03, 06 and 12 months after surgery.

5.7 HISTOPATHOLOGICAL ANALYSIS

Keloids resected during surgery will be stored in neutral buffered 10% formalin solution and then processed and obtained histological sections that will be stained with H&E and picrosirius for analysis of tissue morphological aspects.

5.7.1 Fibroblast analysis

A quantitative analysis of the fibroblasts of the resected keloids will be performed, the histological preparations will be submitted to the staining technique using Hematoxylin-Eosin (HE).

Histological images will be photographed with a digital camera attached to the microscope, under fixed focus and field clarity, obtaining 20 fields per slide at 400x magnification. Photographs will be randomly chosen and analyzed using computer software.

5.7.2 Collagen analysis

Additional sections will be stained with PicrosiriusRed (Sigma-Aldrich, St. Louis, MO, USA) and examined by polarized light microscopy Pol-Interferential Photomicroscope (Model 61282, Carl Zeiss, Germany). The images will also be analyzed by the Image J (NIH) program. With the help of this program, the relative area occupied by collagen fibers will be calculated in relation to the total area of ​​the cut(Junqueira et al., 1982; Ribeiro et al., 2015; Andreo et al., 2018).

5.7.3 TGF-β Analysis

5.7.3.1 Extraction and quality control of total RNA

For the extraction of total RNA from the samples, between 80-100 mg of tissue macerated in liquid nitrogen will be used. Then, the tissue will be homogenized in 1 mL of TRIzol reagent (Invitrogen, São Paulo, Brazil), to start the isolation of total RNA, following the manufacturer's instructions. The total RNA concentration and purity will be determined in NanoDrop 2000 (Thermo Scientific) using the 260 and 280 nm wavelengths and their ratio and the samples will be stored at -80°C. To analyze the integrity of the total RNA, 1 μg of this will be analyzed using 1% agarose gel electrophoresis and stained with ethidium bromide.

5.7.3.2 Synthesis of complementary DNA (CNA) and quantitative real-time PCR (qPCR)

The total RNA will be subjected to the reverse transcription reaction using the *High Capacity cDNA Reverse Transcriptions Kit* (Applied Biosystems) to obtain the cDNA and it will be used for the real-time PCR reaction using the “SYBR Green Kit” (Applied Biosystems). For this procedure the samples will be analyzed in triplicate. Reactions will be performed using the Thermocycler 7500 Real-Time PCR System (Applied Biosystems Carlsbad, CA, USA)it's theprimers forward and reverse specific for theTGF-β and for the constitutive GAPDH will be used to perform this procedure. The quantification will be performed using the 2 − ∆∆CT method(Livak & Schmittgen, 2001) and the control group will be used as a reference.

Table x. Primer used

| **Primer** | **Forward (5' – 3')** | **Reverse (3' – 5')** |
| --- | --- | --- |
| TGF-β | TCCAACCCAGGTCCTTCCTAAAGT | CCCCTGGAAAGGGCTCAACAC |

5.7.3.3 analysis of protein expression by ELISA (enzyme-linked immunosorbent assay)

The muscle extracts will also be used for protein expression analysis, being obtained by maceration of muscle tissue in liquid nitrogen and homogenized with RIPA extraction buffer and 0.1 mM phenylmethylsulfonyl fluoride (PMSF). The tissue extract will be centrifuged at 10,000 rpm for 10 minutes at 4 °C and the supernatant will be stored at -80 °C until the moment of analysis. The quantification of total protein will be performed in NanoDrop 2000 (Thermo Scientific), using wavelengths of 260 and 280 nm. The protein expression of TGF-β in muscle extracts will be evaluated by means of the immunoenzymatic ELISA assay using commercial TGF-β kits (R&D Systems,Minneapolis, USA) following the manufacturer's instructions.

5.7.3.4 Statistical analysis

The normality distribution of the data will be evaluated by the Kolmogorov-Smirnov test. Data with parametric distribution will be submitted to One-way ANOVA test followed by Tukey test for comparison between groups. Data with non-parametric distribution will be submitted to the Kruskal-Wallis test followed by the Dunn's test for comparison between groups.The significance level adopted will be α=5%.

6 OBTAINING PHOTOGRAPHS

In the office during the initial evaluation, keeping all ethical precepts and patient privacy, digital photographs of the keloid scar will be taken in the following incidences: anterior; posterior, superior and left and right sides in order to give a global view of the scar, a millimeter ruler will be placed during the act of photography. The same digital camera will be used for all photographs, using white LED lighting, with the same background color pattern, 20 cm away from the scar in manual macro mode, and with the same focus distance, the same pattern being followed in the evaluation of 3, 6, and 12 months. Each scar will be photographed in 5 incidences totaling 20 photographs per patient during the study period.

The images will be stored in a computer and virtual disk, keeping all the security precepts and with access only to the researcher.

7 OUTCOMES

The expected outcomes with the studied protocol according to the variables would be:

1. Decrease in the area of ​​the resected keloid compared to pre-treatment in the modeling analysis by optical coherence tomography;
2. Improvement of scar appearance and non-recurrence through specialized clinical assessment using the Vancouver Scar Assessment Questionnaire (VSS);
3. Improvement in the quality of life of the patient verified through the Qualifibro-UNIFESP questionnaire;
4. Patient satisfaction when the scar is residual, verified through the satisfaction assessment part of the PSAQ questionnaire;
5. Decrease and organization of fibroblasts and collagen and TGF-β through histopathological analysis of resected keloids.

8 SCHEDULE

| **EVENT** | **INITIAL DATE** | **FINAL DATE** |
| --- | --- | --- |
| Presentation of the protocol at events | 12/21/2020 | 12/31/2021 |
| Experiment | 08/02/2021 | 12/31/2021 |
| Analysis of results | 07/01/2021 | 01/31/2022 |
| Writing of scientific articles | 07/01/2022 | 03/31/2023 |
| Recruitment of participants | 01/04/2021 | 07/30/2021 |
| Literature revision | 12/21/2020 | 07/31/2022 |
| Database organization | 06/01/2021 | 12/31/2021 |

9 BUDGET

| **DESCRIPTION** | **UNITARY VALUE** | **THE AMOUNT** | **TOTAL** |
| --- | --- | --- | --- |
| 100UI Disposable Procedure Glove Box | 25.00 | 02 | 50.00 |
| 50UI Sterile Disposable Surgical Glove Box | 80.00 | 02 | 160.00 |
| Disposable surgical scalpels No. 15 | 4.60 | 60 | 276.00 |
| 18G disposable needles  12 x 40mm | 0.20 | 100 | 20.00 |
| 26G disposable needles  4.5 x 13mm | 0.20 | 100 | 20.00 |
| 3ml Luer Lock Disposable Syringes | 0.50 | 100 | 50.00 |
| Biopsy Vials | 1.00 | 200 | 200.00 |
| Formaldehyde 10% 1L | 45.00 | two | 90.00 |
| Disposable Mask Box with 50UI | 10.00 | 04 | 40.00 |
| Disposable Cap Box with 100UI | 10.00 | 01 | 10.00 |
| Alcoholic chlorhexidine 0.5% in 100ml | 15.00 | 10 | 150.00 |
| 2% lidocaine + epinephrine | 8.00 | 25 | 200.00 |
| Hypoallergenic beige micropore | 25.00 | 5 | 125.00 |
| Printed |  |  | 250.00 |
| sterile gauze | 0.50 | 200 | 100.00 |
| Statistical analysis |  | 1 | 2500.00 |
| 0.9% saline 10ml | 0.20 | 100 | 20.00 |
| Sterilization costs | 5.00 | 30 | 150.00 |
| Matte slide box for microscopic 26x76mm 50UI | 20.00 | 03 | 60.00 |
| Polygrecrapone-25 suture thread or similar | 12.00 | 60 | 720.00 |
| Nylon suture thread | 2.00 | 60 | 120 |
| Triancil 20mg/ml | 14.00 | 116 | 1624.00 |
| TOTAL |  |  | 6935.00 |

10 BIBLIOGRAPHIC REFERENCES

ACKERMAN, B et al. Fibrosing dermatitis In: Histologic diagnosis of inflammatory skin diseases: an algorithmic method based on patern analysis. 2ed Baltimore, Williams and Wilkins. P.727-33, 1997.

ANDREO L, MOSQUITA-FERRARI RA, RIBEIRO ÁBG, BENITTE A. Effects of Myogenic Precursor Cells (C2C12) Transplantation and Low-Level Laser Therapy on Muscle Repair. Lasers Surg Med. 2018; 50(7):781–91.

ALSHARNOUBI et al. Evaluation of scars in children after treatment with low-level laser. Lasers Med Sci. 2018. 33, 1991–1995.

ARNO AI, GAUGLITZ GG, BARRET JP, JESCHKE MG. Up-to-date approach to managing keloids and hypertrophic scars: a useful guide. Burns. 2014;40(7):1255-66.

BAROLET D, BOUCHER A. Prophylactic low-level light therapy for the treatment of hypertrophic scars and keloids: a case series. Lasers Surg Med 2010; 42:597-601.

BEER TW, LAM MH, HEENAN PJ. Tumors of fibrous tissue involving the skin. In: Lever's Histopathogy of the Skin, 10th ed, Elder DE (Ed), Wolters-Kluwer, Lippincott, Williams, & Wilkins, Philadelphia 2008. p.969.

BERMAN B, FLORES F. Recurrence rates of excised keloids treated with postoperative triamcinolone acetonide injections or interferon alfa-2b injections. J Am Acad Dermatol. 1997 Nov;37(5 Pt 1):755-7.

BERMAN B, MADERAL A, RAPHAEL B. Keloids and hypertrophic Scars: pathophysiology, classification, and treatment. Dermatol Surg. 2017;43(Suppl 1):S3–S18.

BETARBET U, BLALOCK TW. Keloids: A Review of Etiology, Prevention, and Treatment. J Clin Aesthet Dermatol. 2020;13(2):33-43.

BOCK O, SCHMID-OTT G, MALEWSKI P, MROWIETZ U. Quality of life of patients with keloid and hypertrophic scarring. Arch Dermatol Res 2006;297:433–438.

BROWN JJ, BAYAT A. Genetic susceptibility to raised dermal scarring. Br J Dermatol 2009; 161:8.

BONATTI S, HOCHMAN B, TUCCI-VIEGAS VM, et al. In vitro effect of 470 nm LED (Light Emitting Diode) in keloid fibroblasts. Brazilian Surgical Minutes. 2011 Feb;26(1):25-30.

FIELDS, ACL, BORGES-WHITE, A, GROTH, AK. Wound healing. ABCD, file bras. cir. dig., São Paulo, v. 20, no. 1, p. 51-58, Mar. 2007.

OAK, ALCANTARA, KAMAMOTO, CRESSONI AND CASAROTTO. Effects of low-level laser therapy on pain and scar formation after inguinal herniation surgery: a randomized controlled single-blind study. Photomed Laser Surg. 2010 Jun;28(3):417-22.

CHEN AD, CHEN RF, LI YT, HUANG YT, LIN SD, LAI CS, KUO YR. Triamcinolone Acetonide Suppresses Keloid Formation Through Enhancing Apoptosis in a Nude Mouse Model. Ann Plast Surg. 2019 83(4S Suppl 1):S50-S54.

CHEN B, DING J, JIN J, SONG N, LIU Y. Continuous tension reduction to prevent keloid recurrence after surgical excision: preliminary experience in Asian patients [published online ahead of print, 2020 May 10]. Dermatol Ther. 2020; e13553. doi:10.1111/dth.13553.

CHEN Y, GAO JH, LIU XJ, et al. Characteristics of occurrence for Han Chinese familial keloids. Burns 2006; 32:1052.

CHIN GS, LIU W, PELED Z, LEE TY, STEINBRECH DS, HSU M, et al. Differential expression of transforming growth factor-beta receptors I and II and activation of Smad 3 in keloid fibroblasts. Plast Reconstruction Surg 2001; 108:423-429.

CLARK RAF: Wound repair. In: Kumar, Robbins, Cotran: Pathologic Basis of Disease, 7th ed., Ed. Saunders, p.112, 2005.

CLARK JA, TURNER ML, HOWARD L, et al. Description of familial keloids in five pedigrees: evidence for autosomal dominant inheritance and phenotypic heterogeneity. BMC Dermatol 2009; 9:8.

COLWELL AS, PHAN TT, KONG W, et al. Hypertrophic scar fibroblasts have increased connective tissue growth factor expression after transforming growth factor-beta stimulation. Plast Reconstruction Surg 2005; 116:1387.

DAVISON SP, DAYAN JH, CLEMENS MW, et al. Efficacy of intralesional 5-fluorouracil and triamcinolone in the treatment of keloids. Aesthet Surg J 2009; 29:40.

DE CICCO L, VISCHIONI B, VAVASSORI A, GHERADI F, JERECZEK BA, LAZZARI R, et al. Postoperative management of Keloids: low-dose-rate and high-dose-rate brachyterapy. Brachytherapy. 2014; 13(5): 508.

DE FREITAS LF, HAMBLIN MR. Proposed Mechanisms of Photobiomodulation or Low-Level Light Therapy. IEEE J Sel Top Quantum Electron. 2016;22(3):7000417. doi:10.1109/JSTQE.2016.2561201

DURANI P, MCGROUTHER DA, FERGUSON MW. The Patient Scar Assessment Questionnaire: a reliable and valid patient-reported outcomes measure for linear scars. Plast Reconstruction Surg. 2009 May;123(5):1481-9.

EPSTEIN et al. Photobiomodulation Therapy Alleviates Tissue Fibrosis Associated with Chronic Graft-Versus-Host Disease: Two Case Reports and Putative Anti-Fibrotic Roles of TGF-β. Photomed Laser Surg. 2018 Feb;36(2):92-99.

EROL OO, GURLEK A, AGAOGLU G, et al. Treatment of hypertrophic scars and keloids using intense pulsed light (IPL). Aesthetic Plast Surg 2008; 32:902.

FERNANDES KPS, FERRARI RAM, FRANCE CM. Biophotonics: Concepts and Applications. São Paulo: Nove de Julho University; 2017.

FERREIRA LM. UNIFESP/EPM Outpatient and Hospital Medicine Guides: Plastic Surgery. Barueri: Manole; 2007.

FITZPATRICK TB. The validity and practicality of sun-reactive skin types I through VI. Arch Dermatol. 1988 Jun;124(6):869-71.

FREITAS, MELO, ALEXANDRINO AND NIGHTS. Efficacy of low-level laser therapy on scar tissue, Journal of Cosmetic and Laser Therapy. 2013 15:3, 171-176.

FRIGO L, FÁVERO GM, LIMA HJ, et al. Low-level laser irradiation (InGaAlP-660 nm) increases fibroblast cell proliferation and reduces cell death in a dose-dependent manner. Photomed Laser Surg. 2010 Aug;28 Suppl 1:S151-6.

FUJII et al., low reactive level laser therapy (lllt) for the treatment of hypertrophic scars and keloids. A re-introduction. Laser Therapy. 2008, 17(1): 35-43.

FUJIWARA M, MURAGAKI Y, OOSHIMA A. Keloid-derived fibroblasts show increased secretion of factors involved in collagen turnover and depend on matrix metalloproteinase for migration. Br J Dermatol 2005; 153:295.

THEFT FMP. Translation into Portuguese, cultural adaptation and reliability of the Questionnaire of Quality of Life for Patients with Keloid and Hypertrophic Scarring [dissertation]. São Paulo: Paulista School of Medicine, Federal University of São Paulo, 2008.

GOLD MH, MCGUIRE M, MUSTOE TA et al. Updated international clinical recommendations on scar management: part 2—algorithms for scar prevention and treatment. Dermatol Surg. 2014;40(8):825–831.

GUIMARÃES, LSP; HIRAKATA, VN. Use of the generalized estimating equation model in longitudinal data analysis. HCPA Magazine. Porto Alegre. Vol. 32, no. 4 (2012), p. 503-511.

GUPTA S, KALRA A. Efficacy and safety of intralesional 5-fluorouracil in the treatment of keloids. Dermatology. 2002;204(2):130-132.

HAHN JM et al. Partial epithelial-mesenchymal transition in keloid scars: regulation of keloid keratinocyte gene expression by transforming growth factor-β1. Burns Trauma 4(1):30. 2016

HAMBLIN MR. Mechanisms and Mitochondrial Redox Signaling in Photobiomodulation. Photochem Photobiol. 2018;94(2):199-212.

HAMRICK M, BOSWELL W, CARNEY D. Successful treatment of earlobe keloids in the pediatric population. J Pediatr Surg 2009; 44:286.

HAN B, FAN J, LIU L, et al. Adipose-derived mesenchymal stem cells treatments for fibroblasts of fibrotic scar via downregulating TGF-β1 and Notch-1 expression enhanced by photobiomodulation therapy. Lasers Med Sci. 2019;34(1):1-10.

HAISA M, OKOCHI H, GROTENDORST GR. Elevated levels of PDGF alpha receptors in keloid fibroblasts contribute to an enhanced response to PDGF. J Invest Dermatol 1994; 103:560.

[HAR-SHAI Y, DUJOVNY E, ROHDE E, ZOUBOULIS CC. Effect of skin surface temperature on skin pigmentation during contact and intralesional cryosurgery of keloids. J Eur Acad Dermatol Venereol 2007; 21:191.](https://www.uptodate.com/contents/keloids-and-hypertrophic-scars/abstract/69)

HAWKINS D, ABRAHAMSE H. Effect of multiple exposures of low-level laser therapy on cellular responses of wounded human skin fibroblasts. Photomed Laser Surg. 2006 Dec;24(6):705-14.

HERASCU, VELCIU, CALIN, SAVASTRU AND TALIANU. Low-level laser therapy (LLLT) efficacy in post-operative wounds. Photomed Laser Surg. 2005 Feb;23(1):70-3.

HEWEDY ES, SABAA BEI, MOHAMED WS, HEGAB DS. Combined intralesional triamcinolone acetonide and platelet rich plasma versus intralesional triamcinolone acetonide alone in treatment of keloids [published online ahead of print, 2020 Mar 4]. J Dermatolog Treat. 2020;1-7.

HOCHMAN B, ISHIZUKA CK, FERREIA LM, OLIVEIRA LQR, LOCALI RF. Revision. Estima – Brazilian Journal of Enterostomal Therapy, [S. l.], v. 2, n. 3, 2004. Available at: https://www.revistaestima.com.br/estima/article/view/154. Accessed on: 15 Nov. 2020.

HU Y, ZHANG C, LIS, JIAO Y, QI T, WEI G, HAN G. Effects of Photodynamic Therapy Using Yellow LED-light with Concomitant Hypocrellin B on Apoptotic Signaling in Keloid Fibroblasts. Int J Biol Sci. 2017;13(3):319-326.

HUU ND, HUU SN, THI XL, VAN TN, MINH PPT, MINH TT, et. al. Successful Treatment of Intralesional Triamcilonon Acetonide Injection in Keloid Patients. Open Access Maced J Med Sci. 2019; 28;7(2):275-278.

JAGADEESAN J, BAYAT A. Transforming growth factor beta (TGFbeta) and keloid disease. Int J Surg 2007; 5:278-285.

JIN R, HUANG X, LI H, et al. Laser therapy for prevention and treatment of pathologic excessive scars. Plast Rebuild Surg 2013; 132:1747.

JUNG JY, ROH MR, KWON YS, CHUNG KY. Surgery and perioperative intralesional corticosteroid injection for treating earlobe keloids: a korean experience. Ann Dermatol 2009; 21:221.

JUNQUEIRA LCU, MONTES GS, SANCHEZ IN. The influence of tissue section thickness on the study of collagen by the Picrosirius-polarization method. Histochemistry. 1982; 74(1):153–6.

KARU TI, KOLYAKOV SF. Exact action spectrum for cellular responses relevant to phototherapy. Photomed Laser Surg. 2005;23(4):355-61.

KIIL J. Keloids treated with topical injections of triamcinolone acetonide (kenalog). Immediate and long-term results. Scand J Plast Reconstruct Surg 1977; 11:169.

KIKUCHI K, KADONO T. TAKEHARA K. Effects of various growth factors and histamine on cultured keloid fibroblasts. Dermatology, 190: 4-8, 1995.

LIANG KY, ZEGER SL. Longitudinal data analysis using generalized linear models. Biometrics. 1986;73(1):13-22.

LEE HS, JUNG SE, KIM SK, KIM YS, SOHN S, KIM YC. Low-Level Light Therapy with 410 nm Light Emitting Diode Suppresses Collagen Synthesis in Human Keloid Fibroblasts: An In Vitro Study. Ann Dermatol. 2017;29(2):149-155.

LEE PENG G, KEROLUS JL. Management of Surgical Scars. Facial Plast Surg Clin North Am. 2019 Nov;27(4):513-517

LEE YI, KIM J, YANG CE, HONG JW, LEE WJ, LEE JH. Combined Therapeutic Strategies for Keloid Treatment. Dermatol Surg. 2019 Jun;45(6):802-810.

LEDON JA, SAVAS J, FRANCA K et al. Intralesional treatment for keloids and hypertrophic scars: a review. Dermatol Surg. 2013;39(12):1745–1757.

LEV-TOV H, BRODY N, SIEGEL D, JAGDEO J. Inhibition of fibroblast proliferation in vitro using low-level infrared light-emitting diodes. Dermatol Surg. 2013 Mar;39(3 Pt 1):422-5.

LIMANDJAJA GC, NIESSEN FB, SCHEPER RJ, GIBBS S. The Keloid Disorder: Heterogeneity, Histopathology, Mechanisms and Models. Front Cell Dev Biol. 2020 May 26;8:360.

LIVAK KJ, SCHMITTGEN TD. Analysis of relative gene expression data using real-time quantitative PCR and the 2-ΔΔCT method. Methods. 2001; 25(4):402–8.

MAHDAVIAN DELAVARY B, VAN DER VEER WM, FERREIRA JA, NIESSEN FB. Formation of hypertrophic scars: evolution and susceptibility. J Plast Surg Hand Surg 2012; 46:95.

MANKOWSKI P, KANEVSKY J, TOMLINSON J, et al. Optimizing Radiotherapy for Keloids: A Meta-Analysis Systematic Review Comparing Recurrence Rates Between Different Radiation Modalities. Ann Plast Surg 2017; 78:403.

MAMALIS, A., KOO, E., GARCHA, M., MURPHY, WJ, ISSEROFF, RR, & JAGDEO, J. (2016). High fluence light emitting diode-generated red light modulates characteristics associated with skin fibrosis. Journal of Biophotonics, 9(11-12), 1167-1179.

MAMALIS AD, LEV-TOV H, NGUYEN DH, JAGDEO JR. Laser and light-based treatment of Keloids--a review. J Eur Acad Dermatol Venereol 2014; 28:689-699.

MARNEROS AG, KRIEG T. Keloids--clinical diagnosis, pathogenesis, and treatment options. J Dtsch Dermatol Ges 2004; 2:905.

MARNEROS AG, NORRIS JE, OLSEN BR, REICHENBERGER E. Clinical genetics of familial keloids. Arch Dermatol 2001; 137:1429.

MIGNON C, UZUNBAJAKAVA NE, CASTELLANO-PELLICENA I, BOTCHKAREVA NV, TOBIN DJ. Differential response of human dermal fibroblast subpopulations to visible and near-infrared light: Potential of photobiomodulation for addressing cutaneous conditions. Lasers Surg Med. 2018 Oct;50(8):859-882.

MOHAMMADI AA, KARDEH S, MOTAZEDIAN GR, SOHEIL S. Management of Ear Keloids Using Surgical Excision Combined with Postoperative Steroid Injections. World J Plast Surg. 2019;8(3):338-344.

MOTOKI THC, ISOLDI FC, BRITO MJA, FILHO AG, FERREIRA LM. Keloid negatively affects body image. Burns. 2019;45(3):610-614.

NAKASHIMA M, CHUNG S, TAKAHASHI A, et al. A genome-wide association study violated four susceptibility loci for keloid in the Japanese population. Nat Genet 2010; 42:768.

NANGOLE FW, AGAK GW. Keloid pathophysiology: fibroblast or inflammatory disorders? JPRAS Open. 2019 Dec;22:44-54.

NELIGAM PC, GURTNER GC. Plastic Surgery: Principles. Translation: Facina T et al. 3rd ed, Vol 1. Rio de Janeiro: Elsevier; 2015.

NOISHIKI C, HAYASAKA Y, OGAWA R. Sex Differences in Keloidogenesis: An Analysis of 1659 Keloid Patients in Japan. Dermatol Ther (Heidelb). 2019 Dec;9(4):747-754.

OGAWA R, YOSHITATSU S, YOSHIDA K, MIYASHITA T. Is radiation therapy for keloids acceptable? The risk of radiation-induced carcinogenesis. Plast Rebuild Surg 2009; 124:1196.

OJEA AR, MADI O, NETO RM et al. Beneficial Effects of Applying Low-Level Laser Therapy to Surgical Wounds After Bariatric Surgery. Photomed Laser Surg. 2016 Nov;34(11):580-584.

OPLÄNDER C, HIDDING S, WERNERS FB, BORN M, PALLUA N, SUSCHEK CV. Effects of blue light irradiation on human dermal fibroblasts. J Photochem Photobiol B. 2011 May 3;103(2):118-25.

OTA AS. Translation into Portuguese, cultural adaptation to Brazil and validation of the Patient Scar Assessment Questionnaire. 2016. 155f. Dissertation (Masters) - Paulista School of Medicine, Federal University of São Paulo (UNIFESP). São Paulo, 2016.

PARK et al. Prevention of Thyroidectomy Scars in Asian Adults With Low-Level Light Therapy. Dermatol Surg. 2016 Apr;42(4):526-34.

PLACIK, OJ, LEWIS, VL. Immunologic associations of keloids. Surg. Gynecol.obstet. 175:186-93, 1992.

POTTER DA, VEITCH D, JOHNSTON GA. Scarring and wound healing. Br J Hosp Med (Lond). 2019 Nov 2;80(11):C166-C171

[QU L, LIU A, ZHOU L, et al. Clinical and molecular effects on mature burn scars after treatment with a fractional CO(2) laser. Lasers Surg Med 2012; 44:517.](https://www.uptodate.com/contents/keloids-and-hypertrophic-scars/abstract/19)

RAMOS et al. Photobiomodulation Improved the First Stages of Wound Healing Process After Abdominoplasty: An Experimental, Double-Blinded, Non-randomized Clinical Trial. Aesthetic Plast Surg. 2019 Feb;43(1):147-154.

KINGS, ALN. Main characteristics of keloid scars. In bras dermatol 69 (6) 495-7, 1994.

SANTOS MC, TIBOLA J, MARQUES CMG. Translation, revalidation and reliability of Vancouver Healing for Portuguese – Brazil. Rev Bras Burns. 2014; 13:26-30.

SARRAZY V, BILLET F, MICALLEF L, et al. Mechanisms of pathological scarring: role of myofibroblasts and current developments. Wound Repair Regen 2011; 19 Suppl 1:s10.

SAYAH DN, SOO C, SHAW WW, et al. Downregulation of apoptosis-related genes in keloid tissues. J Surg Res 1999; 87:209.

SHIH B, BAYAT A. Genetics of keloid scarring. Arch Dermatol Res 2010; 302:319.

SHIN J, CHO JT, PARK SI, JUNG SN. Combination therapy using non-ablative fractional laser and intralesional triamcinolone injection for hypertrophic scars and keloids treatment. Int Wound J. 2019; 1-7.

SHIN JU, KIM SH, KIM H, et al. TSLP Is a Potential Initiator of Collagen Synthesis and an Activator of CXCR4/SDF-1 Axis in Keloid Pathogenesis. J Invest Dermatol 2016; 136:507.

SHIN JY, LEE JW, ROH SG, LEE NH, YANG KM. A Comparison of the Effectiveness of Triamcinolone and Radiation Therapy for Ear Keloids after Surgical Excision: A Systematic Review and Meta-Analysis. Plast Reconstruction Surg. 2016;137(6):1718-25.

SCLAFANI AP, GORDON L, CHADHA M, ROMO T 3rd. Prevention of earlobe keloid recurrence with postoperative corticosteroid injections versus radiation therapy: a randomized, prospective study and review of the literature. Dermatol Surg 1996; 22:569.

SHONS AIR, PRESS BH. The treatment of earlobe keloids by surgical excision and postoperative triamcinolone injection. Ann Plast Surg 1983; 10:480.

SILVA JP, DA SILVA MA, ALMEIDA AP, LOMBARDI JUNIOR I, MATOS AP. Laser therapy in the tissue repair process: a literature review. Photomed Laser Surg 2010;28:17-21.

SULLIVAN T, SMITH J, KERMODE J, MCLVER E, COURTEMANCHE DJ. Rating the burn scar. J Burn Care Rehabilitation 1990 May-Jun;11(3):256-60.

SYED F, AHMADI E, IQBAL SA et al. Fibroblasts from the growing margin of keloid scars produce higher levels of collagen I and III compared with intralesional and extralesional sites: clinical implications for lesional site-directed therapy. Br J Dermatol. 2011;164(1):83–96.

TAN KT, SHAH N, PRITCHARD SA et al. The influence of surgical excision margins on keloid prognosis. Ann Plast Surg. 2010;64(1):55–58.

TRICARICO PM, ZUPIN L, OTTAVIANI G, PACOR S, JEAN-LOUIS F, BONIOTTO M, et al. Photobiomodulation therapy promotes in vitro wound healing in nicastrin KO HaCaT cells. J Biophotonics. 2018;11(12): 1-10.

VAN DROOGE AM, VRIJMAN C, VAN DER VEEN W, WOLKERSTORFER A. A randomized controlled pilot study on ablative fractional CO2 laser was consecutive patients presenting with various scar types. Dermatol Surg 2015; 41:371.

VAN LEEUWEN MC, BULSTRA AE, VAN LEEUWEN PA, NIESSEN FB. A new argon gas-based device for the treatment of keloid scars with the use of intralesional cryotherapy. J Plast Reconstr Aesthet Surg 2014; 67:1703.

VAN LEEUWEN MC, VAN DER WAL MB, BULSTRA AE, et al. Intralesional cryotherapy for treatment of keloid scars: a prospective study. Plast Rebuild Surg 2015; 135:580.

VRIJMAN C, VAN DROOGE AM, LIMPENS J, et al. Laser and intense pulsed light therapy for the treatment of hypertrophic scars: a systematic review. Br J Dermatol 2011; 165:934.

WOLFRAM D, TZANKOV A, PÜLZL P, PIZA-KATZER H. Hypertrophic scars and keloids--a review of their pathophysiology, risk factors, and therapeutic management. Dermatol Surg. 2009 Feb;35(2):171-81.

ZEGER SL, LIANG KY. Longitudinal data analysis for discrete and continuous outcomes. Biometrics. 1986;42(1):121-30.

[**11 APPENDICES**](https://www.translatoruser.net/bvsandbox.aspx?&from=pt&to=en&csId=7cbd1804-770d-45ff-8393-cfcf92b9f09c&usId=4cad987a-2541-46e6-8bda-ed588642f2c0&ac=true&bvrpx=false&bvrpp=&dt=2021%2F4%2F2%2017%3A27#_Toc59456921)

[11.1 APPENDIX 1- FREE AND INFORMED CONSENT TERM](https://www.translatoruser.net/bvsandbox.aspx?&from=pt&to=en&csId=7cbd1804-770d-45ff-8393-cfcf92b9f09c&usId=4cad987a-2541-46e6-8bda-ed588642f2c0&ac=true&bvrpx=false&bvrpp=&dt=2021%2F4%2F2%2017%3A27#_Toc59456922)

**TCLE - Free and informed Consent Form for Participation in Clinical Research:**

Participant name:______

Address:_______________________ZIP CODE _____________City_____________

Email: ___________________________________RG:____

**1.Title of the Experimental Work:**Study of the achievement of the association of photobiomodulation therapy and the intralesional application of corticosteroids in the pre and postoperative exeresis of queloids: a controlled, randomized and double-blind study.

**2.Objective:**The main objective of this study is to analyze the effect of photobiomodulation (use of light) as a treatment method to prevent the loloid from returning after being removed by surgery. As secondary objectives check if the scar after the use of this treatment looks better.

**3.Justification:**Healing errors such as loloides are difficult to treat, there is today in the medical area no treatment that says it will be cured in 100% of cases. Among the best treatments we have the withdrawal by surgery and together use other methods to help that the loloides do not return. These procedures used together with surgery besides not giving certainty that the loloides will not return is often painful and can bring not very good effects. Photobiomodulation (use of light devices) has been shown to be a good tool to be used as a treatment for these scars, so based on other studies the use of it along with surgery can help that keloids do not return, besides being a procedure that does not hurt and does not bring bad side effects like the other treatments that are also used.

**4. Experimental Phase Procedures:**The Lord (a) is being invited to participate in a study in which the scar scar of the type of loloide will be performed through surgery. The surgery procedure will be performed in a surgical room, and local anesthesia can be used, with or without sedation-so as not to feel pain and discomfort, depending on the size of the scar (small and medium scars only with local anesthesia and large if necessary sedation). Before the day of surgery for 30 days once a week will have to attend the outpatient unit to perform the use of light therapy and application in the corticosteroid lesion, for approximately 15 min each week, in the first consultation will also be performed the modeling with silicone of the loloide to analyze its measures (painless and risk-free procedure) and repeated on the day of surgery before the removal of the loloide. After surgery also during the first 30 days will have to attend weekly for approximately 30 min each week for light treatment and corticosteroid application on the scar that remained, and revisions of the surgery. You will also have to return after completing 3 months of the procedure, 6 months of the procedure and 1 year of the procedure, and each time you will have to make available about 30 min for consultation. In all consultations, photographs of the operated site will be taken and questionnaires will be provided to answer about the quality of the scar (PSAQ questionnaire) and quality of life (Qualifibro-UNIFESP). It is emphasized that the study consists of 2 groups, and the participants of each of them will be chosen by lot. One of the groups will be the experimental in which the same therapy will be performed above being light with power for biological effect and another group, called control, where the identical therapy will be used however the light will only have the same color without any effect to simulate the procedure. It is also notepoint that all the removed loloides will go to the laboratory for analysis and then be discarded.

**5th**. **Discomfort or Expected Risks:**The risks and discomforts that the participant may have are: Purple spots at the site of surgery and where some medication is needed; discomfort in the application of the local anesthetic; bleeding after surgery, opening of the scar before even removing the stitches; infections at the operated site; reappearance of the loloide and changes in skin color.

**6. Risk protective measures:**Protective measures related to the risks and discomforts mentioned above are: At any time when one of the changes mentioned in item 5 arises, the participant may go directly to the Emergency Room of the Hospital do Mandaqui at any time, where a member of the research team will be in contact with the evaluating physician of the emergency room, and if necessary will provide assistance in person. All necessary procedures will be carried out to repair and treat any event that occurs.

**7. Research Benefits: As**a direct benefit the patient will perform the removal of the scar of the type of loloide and all follow-up and treatment.

**8. Existing Alternative Methods:**There are several alternative methods to treatment described, none of which guarantees 100% of the cure. Among the main ones are: Use of radiotherapy (use of devices that apply rays), corticosteroid injection, silicone adhesives, corticosteroid adhesives and use of high-power lasers.

**9. Withdrawal of Consent:**In case of withdrawal at any time of the survey, this consent may be withdrawn by the participant at any time, without any prejudice to the participant.

**10. Guarantee of Confidentiality:**All data from this research will be kept confidential, the computers used, as well as photographs, files, questionnaires will only be accessed by analysts and study members, no data will be provided to other institutions or third parties.

**11. Forms of Reimbursement of Expenses arising from Participation in the Survey:**No additional cash resources will be provided to participants. Standard postoperative snack will be provided at the hospital.

**12. Research Site: The**research will be carried out in the Hospital Set of Mandaqui, being the surgeries inside the main building, on the 6th floor, the application of light in the department of biophotonics on the 1st floor and the reviews and evaluations in the specialty outpatient clinic, from Monday to Friday from 7am to 7pm. Address: Rua Voluntários da Pátria, 4301, Mandaqui, São Paulo-SP. Contact phone: (11) 2281-5000.

**13.**Consultations with medical record data contained in your hospital records, such as clinical and laboratory data, will be required. No personal and medical data will be used improperly or passed on to third parties, by signing this term will authorize the researcher to access this information.

**14.**Research Ethics Committee (CEP) is an interdisciplinary and independent collegiate, which must exist in institutions that conduct research involving human beings in Brazil, created to defend the interests of research participants in their integrity and dignity and to contribute to the development of research within ethical standards (Standards and Regulatory Guidelines for Research involving Human Beings - Res. CNS no. 466/12 and Res. CNS 510/2016). The Ethics Committee is responsible for the evaluation and monitoring of research protocols in terms of ethical aspects. Address of **the Research Ethics Committee (CEP) of the Mandaqui Hospital Complex: Rua Voluntários da Pátria, number 4301, prédio 04, casa azul, Santana, São Paulo-SP, CEP 02401-400, phone (11) 2281-5147 or (11) 2281-5179, e-mail:**[**cepchm@gmail.com**](mailto:cepchm@gmail.com)**. Opening hours: Monday to Friday - from 08:00 to 17:00.**

**Address of the Ethics Committee of Uninove: Rua. Vergueiro nº 235/249 - 12º andar - Liberdade - São Paulo - SP CEP. 01504-001. Phone: 3385-9010. E-mail:**[comitedeetica@uninove.br](mailto:comitedeetica@uninove.br)

**Hours of attendance of the Ethics Committee: Monday to Friday - From 11:30 am to 1:00 pm and from 3:30 pm to 7:00 pm.**

**15. Full Name and Telephone Numbers of Researchers (Advisor and Students) for Contact:**Prof. Dr. Raquel Agnelli Mesquita Ferrari - (011) 99919-2988, Dr. Jefferson André Pires- (11) 98181-4120. The participant in case of doubts or complications may call at any time on these phones.

**16th**. Any complications that may arise during the research may be discussed by their own means. If losses or harms are observed during the research, it will be interrupted and all assistance will be provided to the participant. The participant can go directly to the Emergency Room of the Hospital do Mandaqui at any time if emergencies, where a member of the research team will contact the evaluator doctor of the emergency room, and if necessary will provide assistance in person.

**17. Post-Information Consent:**

I, _________ I confirm that I have received a path of this consent form, and I authorize the realization of the research work and the dissemination of the data obtained only in this study in the scientific environment.

_______________________________

Participant's Signature

**18.**I, ______
research), I certify that:

a) This research will only begin after the approval of the aforementioned Research Ethics Committee(s) to which the project was submitted.

b) Whereas ethics in research implies respect for human dignity and the protection due to participants in scientific research involving human beings;

c) This study has scientific merit and the team of professionals duly cited in this term is trained, trained and competent to perform the procedures described in this term;

_________________________________
Signature of the Responsible Researcher

São Paulo,

[11.2 APPENDIX2- DOCUMENT FOR STANDARDIZATION OF SURGERY.](https://www.translatoruser.net/bvsandbox.aspx?&from=pt&to=en&csId=7cbd1804-770d-45ff-8393-cfcf92b9f09c&usId=4cad987a-2541-46e6-8bda-ed588642f2c0&ac=true&bvrpx=false&bvrpp=&dt=2021%2F4%2F2%2017%3A27#_Toc59456923)

**TO THE PLASTIC SURGEONS OF THE HOSPITAL ASSEMBLY OF MANDAQUI**

**STANDARDIZATION FOR THE SURGICAL PROCEDURE OF THE RESEARCH PROJECT**

**TITLE**: Study of the Effect of the association of photobiomodulation therapy and intralesional application of corticosteroids in the pre and postoperative period of the exeresis of fallays: a controlled, randomized and double-blind study.

**RESEARCHER:**Jefferson André Pires

**ADVISOR:**Prof. Dr. Raquel Agnelli Mesquita Ferrari

**1) Application of local anesthesia**

**Solution:**local anesthetic at a concentration of 1:100,000, containing 20 ml of 2% lidocaine (20mg/ml) without vasoconstrictor, 20 ml of bupivacaine hydrochloride 0.5% (5ml/ml), 1ml of epinephrine (1mg/ml) and 60ml of saline solution (0.9%). The solution may change if adequacy is required for the patient's weight, obeying the use of 5mg/kg of lidocaine and 3mg/km of bupivacaine.

**Application:**Use sterile and disposable 10ml syringe with 13x45mm surface needles and 25x7mm needles for depth. The amount of anesthetic will be divided proportionally into the entire length of the scar, and equal amounts are injected into areas of 1cm².

**2) Exeresis procedure**

Use scalpel blades of no. 15, being dried in the form of spindle or ellipse with margins of 5 mm, of healthy skin, measured after the end of the scar. As for depth, the entire scar should be dried until healthy fibrosis-free tissue should be obtained.

**3) Hemostasis procedure**

Perform hemostasis with monopolar or bipolar electrocautery at low intensities (up to 30). It should be performed judiciously only where there is bleeding and if necessary to avoid unnecessary tissue injury.

**4) Skin synthesis procedure**

**Wires:**For subcutaneous cellular tissue, when necessary use polyglecaprone wire 25 (caprofyl®), with diameter 4-0 and circular needle. Subdermal stitches if necessary should use nylon threads with a diameter of 4-0 to 6-0, where the thinnest diameters (5-0 and 6-0) will be used in anatomical sites with less tension and thinner skin and the larger one (4-0) in sites with higher tension and thicker skin.

For skin synthesis, intradermal suture with nylon thread with diameters of 4-0 and 5-0 is used, the first in areas with higher tension and thicker dermis and the second in anatomical sites without tension and with thinner dermis.

**5) Dressing**

After the synthesis procedure, clean the scar with 0.9% saline solution. After performing microporagem in "X".

We appreciate your participation. Best regards

|  |  |
| --- | --- |
|  | 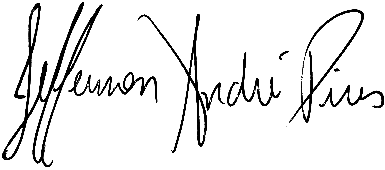 |

**_____________________________________**

[11.3 APPENDIX 3- CONFIDENTIALITY TERM](https://www.translatoruser.net/bvsandbox.aspx?&from=pt&to=en&csId=7cbd1804-770d-45ff-8393-cfcf92b9f09c&usId=4cad987a-2541-46e6-8bda-ed588642f2c0&ac=true&bvrpx=false&bvrpp=&dt=2021%2F4%2F2%2017%3A27#_Toc59456924)

**CONFIDENTIALITY TERMS**

**Project title**: Study of the effect of the association of *photobiomodulation therapy and intralesional application of corticosteroids in the pre and postoperative period of the exeresis of fallaides: a controlled, randomized and double-blind study.*

**Institution/Department: Hospital**Complex of Mandaqui and Nove de Julho University - UNINOVE. Department of Biophonics applied to Health Sciences.

**Data collection site**: Outpatient Clinic of the Plastic SurgeryService, ward and surgical center of the Hospital Complex of Mandaqui. Laboratories of the University Nove de Julho.

The researchers of this project undertake to preserve the privacy of patients whose data will be collected in the outpatient clinic of the plastic surgery service, ward and surgical center of the Hospital Group of Mandaqui. There will also be data collected in the laboratories of the Nove de Julho University. They also agree that such information will be used solely and exclusively for the implementation of this project.

The information will only be disclosed anonymously and maintained in the Department of Biophonics of the Nove de Julho University (UNINOVE) for a period of five years. After this period, the data will be destroyed. As for biological samples, these will be discarded according to the rules of disposal control for biological materials existing in the department.

This research project was reviewed and approved by the Research Ethics Committee of the Hospital Complex of Mandaqui and UNINOVE.

|  |  |
| --- | --- |
|  | **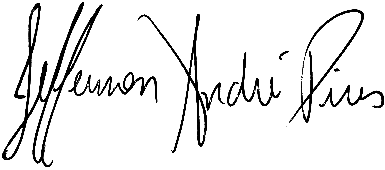** |

**_____________________________________**

[**12 ANNEXES**](https://www.translatoruser.net/bvsandbox.aspx?&from=pt&to=en&csId=7cbd1804-770d-45ff-8393-cfcf92b9f09c&usId=4cad987a-2541-46e6-8bda-ed588642f2c0&ac=true&bvrpx=false&bvrpp=&dt=2021%2F4%2F2%2017%3A27#_Toc59456926)

[12.1 ANNEX 1- CLASSIFICATION OF THE SKIN PHOTOTYPE (FITZPATRICK, 1988)](https://www.translatoruser.net/bvsandbox.aspx?&from=pt&to=en&csId=7cbd1804-770d-45ff-8393-cfcf92b9f09c&usId=4cad987a-2541-46e6-8bda-ed588642f2c0&ac=true&bvrpx=false&bvrpp=&dt=2021%2F4%2F2%2017%3A27#_Toc59456927)

**Fitzpatrick's Skin Phototype Assessment Questionnaire**

Appraiser name:______

Patient ID: _______

Scar location: ______

Date: __/__/____.

| **I-PHYSICAL COMPOSITION** | **0** | **1** | **2** | **3** | **4** |
| --- | --- | --- | --- | --- | --- |
| 1-What color are your eyes? | Blue, green or light gray | Blue, green or gray | Dark blue or green, light brown (hazelnut) | Dark brown | Brownish black |
| 2- What is the natural color of your hair? | red | blond | Dark brown or blonde | Dark brown | black |
| 3-What color is the skin in unexposed areas? | rose | Very pale | Light brown | brown | Dark brown |
| 4- Do you have twill in unexposed areas? | many | Several | few | Rare | No |
| **II-REACTION TO SUN EXPOSURE** | **0** | **1** | **2** | **3** | **4** |
| 1-What happens on the skin if you stay in the sun for an extended period? | Burns a lot, forms bubbles and descama | Moderately burn, form blisters and flaking | Burns sometimes and flaking | Rarely burns | Does not burn |
| 2-Do you get tanned after exposing yourself to the sun? | never | rarely | Sometimes | frequently | always |
| 3-How much tan? | Don't tan | Light tanning | Medium Tanning | Dark Tanning | Too dark |
| 4-Is your face sensitive to the sun? | Very sensitive | sensitive | Slightly sensitive | resistant | Very sturdy |
| **II-REACTION INTENTIONAL EXPOSURE TO THE SUN** | **0** | **1** | **2** | **3** | **4** |
| 1-How often do you tan? | never | rarely | Sometimes | frequently | always |
| 2-How long does tanning last? | >3 months | 2-3 months | 1-2 months | < 1 month | < 2 weeks |

| **Score** | **kind** |
| --- | --- |
| 0-7 | I |
| 8-16 | II |
| 17-25 | III |
| 25-30 | IV |
| 31-34 | V |
| 35 0U MORE | VI |

PATIENT PHOTOTYPE: ___.

[12.2 ANNEX 2 - QUALIFIBRO-UNIFESP QUESTIONNAIRE (FURTADO, 2008)](https://www.translatoruser.net/bvsandbox.aspx?&from=pt&to=en&csId=7cbd1804-770d-45ff-8393-cfcf92b9f09c&usId=4cad987a-2541-46e6-8bda-ed588642f2c0&ac=true&bvrpx=false&bvrpp=&dt=2021%2F4%2F2%2017%3A27#_Toc59456928)

**QUALIFIBRO/PLASTIC SURGERY QUESTIONNAIRE-UNIFESP**

Appraiser name:______

Patient ID: _______

Scar location: ______

Date: __/__/____.

| **Completely false** | **false** | **To some extent true** | **Almost true** | **true** | **Completely true** |  |
| --- | --- | --- | --- | --- | --- | --- |
| 1. Climate changes greatly affect my scars (pain, tension sensation). |  |  |  |  |  |  |
| 2. My scars restrict  (hinder) my  Movements. |  |  |  |  |  |  |
| 3. I can ignore the way  that people look at me for  cause of my scars. |  |  |  |  |  |  |
| 4. The itching in my  scars bothers me  frequently. |  |  |  |  |  |  |
| 5. Because of my scars,  sometimes I'm ashamed of  be sexually active. |  |  |  |  |  |  |
| 6. I find it difficult to bear the  itch in my  scarring. |  |  |  |  |  |  |
| 7. I do my best to avoid  that people close to me  know that I have scars. |  |  |  |  |  |  |
| 8. When my scars  itch, I can't get  without coering them. |  |  |  |  |  |  |
| 9. I don't feel physically  attractive or sexually  desirable when I think of  my scars. |  |  |  |  |  |  |
| 10. I find it difficult to accept my  scarring. |  |  |  |  |  |  |
| 11. I do not go to the pool or  beach because other people  may be disgusted by the  my scars. |  |  |  |  |  |  |
| 12. I never feel  embarrassed or  embarrassed because of the  of my scars. |  |  |  |  |  |  |
| 13. I have less  self-confidence because  of my scars. |  |  |  |  |  |  |
| 14. I don't feel good when  ask me about  my scars. |  |  |  |  |  |  |
| 15. I've thought about committing  suicide because of  my scars. |  |  |  |  |  |  |

[12.3 ANNEX 3- PSAQ QUESTIONNAIRE (OTA, 2016)](https://www.translatoruser.net/bvsandbox.aspx?&from=pt&to=en&csId=7cbd1804-770d-45ff-8393-cfcf92b9f09c&usId=4cad987a-2541-46e6-8bda-ed588642f2c0&ac=true&bvrpx=false&bvrpp=&dt=2021%2F4%2F2%2017%3A27#_Toc59456929)

**Patient Scar Assessment Questionnaire (PSAQ)**

**Part II - Satisfaction classification**

Appraiser name:______

Patient ID: _______

Scar location: ______

Date: __/__/____.

| **I-SATISFACTION WITH THE PARADECA** | **Very pleased** | **satisfied** | **unsatisfied** | **Very Dissatisfied** |
| --- | --- | --- | --- | --- |
| 1. Are you satisfied with the color of your scar compared to the skin around it? |  |  |  |  |
| 2. Are you satisfied with the redness of your scar? |  |  |  |  |
| 3. Are you satisfied with the length of your scar? |  |  |  |  |
| 4. Are you satisfied with the width of your scar? |  |  |  |  |
| 5. Are you satisfied with the height of your scar compared to the skin around it? |  |  |  |  |
| 6. Are you satisfied with the texture of your scar (touch sensation)? |  |  |  |  |
| 7. Are you satisfied with the 'lumps' of your scar? |  |  |  |  |
| 8. Are you satisfied with the 'glow' of your scar? |  |  |  |  |
| 9. Overall, are you satisfied with the appearance of your scar? |  |  |  |  |
| **II-SATISFACTION WITH SYMPTOMS** | **Very pleased** | **satisfied** | **unsatisfied** | **Very Dissatisfied** |
| 1. Are you satisfied with the itching caused by your scar? |  |  |  |  |
| 2. Are you satisfied with the pain caused by your scar? |  |  |  |  |
| 3. Are you satisfied with the discomfort caused by your scar? |  |  |  |  |
| 4. Are you satisfied with the numbness caused by your scar? |  |  |  |  |
| 5. Are you satisfied with the strange sensations caused by your scar? |  |  |  |  |
| 6. Overall, you are satisfied with the problems caused by your  scar? |  |  |  |  |

[12.4 ANNEX 4- VANCOUVER SCAR SCALE QUESTIONNAIRE (SANTOS, 2014)](https://www.translatoruser.net/bvsandbox.aspx?&from=pt&to=en&csId=7cbd1804-770d-45ff-8393-cfcf92b9f09c&usId=4cad987a-2541-46e6-8bda-ed588642f2c0&ac=true&bvrpx=false&bvrpp=&dt=2021%2F4%2F2%2017%3A27#_Toc59456930)

**VANCOUVER SCAR SCALE**

Appraiser name:______

Patient ID: _______

Scar location: ______

Date: __/__/____.

| **pigmentation**  0.    normal  1.    HYPOPIGMENTATION  2.    Hyperpigmentation | **Vascularity**  0.    NORMAL -Similar to the rest of the body  1.    Pink  2.    Reddish  3.    purple |
| --- | --- |
| **flexibility**  0.    normal  1.    MALLEABLE - Flexible to minimum resistance.  2.    DEFORMATION- Gives in under pressure.  3.    FIRM- Inflexible, does not move easily resistant to manual pressure.  4.    BANDS- Shape of whitish rope in its extension.  5.    CONTRACTURE- Shortening of the scar, producing deformity or distortion. | **height**  0.    NORMAL - Flat  1.    < 2mm  2.    < 5mm  3.    > 5mm |
