# Supplementary material for: Effect of the combination of photobiomodulation therapy and the intralesional administration of corticoid in the preoperative and postoperative periods of keloid surgery: A randomized, controlled, double-blind trial protocol study
Source: PLoS One. 2022 Feb 15;17(2):e0263453. doi: 10.1371/journal.pone.0263453 (PMC8846523; doi:10.1371/journal.pone.0263453)
Supplement: S3 File — (DOCX) [file pone.0263453.s003.docx]

CONJUNTO HOSPITALAR DE

MANDAQUI - CHM

**PARECER CONSUBSTANCIADO DO CEP**

**DADOS DO PROJETO DE PESQUISA**

**Título da Pesquisa:**TRATAMENTO DA SÍNDROME DO NEVO BASOCELULAR COM MÚLTIPLAS CIRURGIAS- RELATO DE CASO E REVISÃO DE LITERATURA

**Pesquisador:** JEFFERSON ANDRE PIRES

**Área Temática:**

**Versão:** 1

**CAAE:** 36967820.1.0000.5551

**Instituição Proponente:** SAO PAULO SECRETARIA DA SAUDE

**Patrocinador Principal:** Financiamento Próprio

**DADOS DO PARECER**

**Número do Parecer:** 4.281.616

**Apresentação do Projeto:**

A síndrome do nevo basocelular ou síndrome de Gorlin é uma patologia genética rara. Seu diagnóstico é clínico e pode apresentar diversas manifestações. O tratamento desta patologia deve ser multidisciplinar se houver manifestações clínicas além dos múltiplos carcinomas basocelulares (CBC’s), e na presença apenas destes a exérese cirúrgica ou tratamentos para o câncer de pele como já preconizado. O objetivo deste artigo é o relato de caso do tratamento com múltiplas cirurgias sequenciadas de um paciente com diagnóstico clínico da síndrome de Gorlin, apresentando como única manifestação os CBC’s. O tratamento preconizado tem sido efetivo e seguro com satisfação do paciente do ponto de vista oncológico, estético e funcional.

**Objetivo da Pesquisa:**

O objetivo deste trabalho é realizar o relato de caso de uma apresentação da síndrome do nevo basocelular sem outras manifestações clínicas além dos múltiplos carcinomas basocelures, bem como relatar o tratamento cirúrgico realizado no paciente e realizar uma revisão da literatura.

**Avaliação dos Riscos e Benefícios:**

Riscos: Possibilidade de desconforto ou constrangimento do paciente pela exposição da história clínica e cirúrgica em eventos científicos.

Benefícios: Esse relato de caso poderá contribuir indiretamente para o paciente ou grupo de indivíduos que se encontram na mesma situação, bem como auxiliar outros profissionais da

**Endereço:** VOLUNTARIOS DA PATRIA 4301 - Prédio 4 - Casa Azul

| **Bairro:** SANTANA | | **CEP:** 02.401-400 | |
| --- | --- | --- | --- |
| **UF:** SP | **Município:** | SAO PAULO |  |
| **Telefone:** | (19)2281-5147 | **Fax:** (19)2281-5179 | **E-mail:** cepchm@gmail.com |

Página 01 de 03


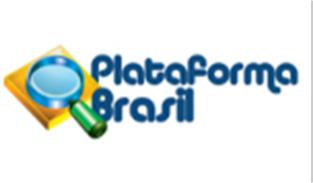
CONJUNTO HOSPITALAR DE

MANDAQUI - CHM

Continuação do Parecer: 4.281.616

cirurgia plástica quanto ao tratamento realizado.

**Comentários e Considerações sobre a Pesquisa:**

Fundamentação racional: A síndrome do nevo basocelular também denominada síndrome do carcinoma nevóide de células basais ou síndrome de Gorlin ou ainda síndrome de Gorlin-Goltz é uma patologia rara, descrita pela primeira vez em 1864 e posteriormente melhor descrita em 1960 por Gorlin e Goltz. Essa patologia é de etiologia genética com caráter autossômico dominante causada por mutações que inativam o gene supressor de tumor PATCH 1 no cromossomo 9q22.3 ou infrequentemente mutações na proteína SUFU no cromossomo 10q24.32, também é descrita variações não patogênicas. A prevalência dessa síndrome é estimada em 1:31.000-164.000 pessoas. O diagnóstico é clínico e pode apresentar diversas manifestações além dos múltiplos carcinomas basocelulares, como tumores odontogênicos, alterações músculo-esqueléticas, neurológicas, endócrinas e oftalmológicas.

Material e métodos estão descritos no projeto, com detalhamento de todo processo e análise de dados. Haverá uso de fontes secundárias de dados (prontuários) e não haverá retenção de amostras para armazenamento em banco.

**Considerações sobre os Termos de apresentação obrigatória:**

Foram apresentados todos os termos considerados obrigatórios.

**Recomendações:**

Comunicar toda e qualquer alteração do projeto e termo de consentimento livre e esclarecido.

**Conclusões ou Pendências e Lista de Inadequações:**

Foram apresentados todos os termos obrigatórios, e após análise do projeto apresentado, concluo pela APROVAÇÃO DO PRESENTE PROJETO DE PESQUISA.

**Considerações Finais a critério do CEP:**

O Colegiado acolheu na íntegra o parecer emitido pelo membro relator.

**Este parecer foi elaborado baseado nos documentos abaixo relacionados:**

|  | Tipo Documento | |  | Arquivo |  | Postagem |  | Autor | Situação | |
| --- | --- | --- | --- | --- | --- | --- | --- | --- | --- | --- |
|  |  | |  |  | |  |  |  |  |  |
|  | Informações | |  | PB_INFORMAÇÕES_BÁSICAS_DO_P | | 13/08/2020 |  |  | Aceito | |
|  |  | |  | | |  |  |  |  |  |
|  |  | | | | |  |  |  |  |  |
|  | **Endereço:** VOLUNTARIOS DA PATRIA 4301 - Prédio 4 - Casa Azul | | | | |  |  |  |  |  |
|  | **Bairro:** SANTANA | | | **CEP:** 02.401-400 | |  |  |  |  |  |
|  | **UF:** SP | **Município:** SAO PAULO | | |  |  |  |  |  |  |
|  | **Telefone:** | (19)2281-5147 | | **Fax:** (19)2281-5179 | **E-mail:** | cepchm@gmail.com | |  |  |  |
|  |  |  |  |  |  |  |  |  |  |  |

Página 02 de 03


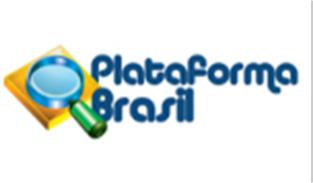
CONJUNTO HOSPITALAR DE

MANDAQUI - CHM

Continuação do Parecer: 4.281.616

| Básicas do Projeto | ETO_1611226.pdf | 15:53:08 |  | Aceito |
| --- | --- | --- | --- | --- |
|  |  |  |  |  |
| Folha de Rosto | Folha_de_rosto.pdf | 13/08/2020 | JEFFERSON | Aceito |
|  |  | 15:52:11 | ANDRE PIRES |  |
| TCLE / Termos de | TERMO_DE_CONFIDENCIALIDADE.do | 13/08/2020 | JEFFERSON | Aceito |
| Assentimento / | cx | 12:19:48 | ANDRE PIRES |  |
| Justificativa de |  |  |  |  |
| Ausência |  |  |  |  |
| TCLE / Termos de | TERMO_DE_AUTORIZACAO_PARA_U | 13/08/2020 | JEFFERSON | Aceito |
| Assentimento / | SO_DE_IMAGEM_EM_PUBLICACAO_ | 12:19:38 | ANDRE PIRES |  |
| Justificativa de | CIENTIFICA.docx |  |  |  |
| Ausência |  |  |  |  |
| TCLE / Termos de | TCLE.docx | 13/08/2020 | JEFFERSON | Aceito |
| Assentimento / |  | 12:19:23 | ANDRE PIRES |  |
| Justificativa de |  |  |  |  |
| Ausência |  |  |  |  |
| Projeto Detalhado / | projeto_sindrome_de_gorlin.docx | 13/08/2020 | JEFFERSON | Aceito |
| Brochura |  | 12:19:09 | ANDRE PIRES |  |
| Investigador |  |  |  |  |

**Situação do Parecer:**

Aprovado

**Necessita Apreciação da CONEP:**

Não

SAO PAULO, 16 de Setembro de 2020

**Assinado por:**

**Sergio Makabe**

**(Coordenador(a))**

**Endereço:** VOLUNTARIOS DA PATRIA 4301 - Prédio 4 - Casa Azul

| **Bairro:** SANTANA | | **CEP:** 02.401-400 | |
| --- | --- | --- | --- |
| **UF:** SP | **Município:** | SAO PAULO |  |
| **Telefone:** | (19)2281-5147 | **Fax:** (19)2281-5179 | **E-mail:** cepchm@gmail.com |

Página 03 de 03
