# Supplementary material for: Effect of the combination of photobiomodulation therapy and the intralesional administration of corticoid in the preoperative and postoperative periods of keloid surgery: A randomized, controlled, double-blind trial protocol study
Source: PLoS One. 2022 Feb 15;17(2):e0263453. doi: 10.1371/journal.pone.0263453 (PMC8846523; doi:10.1371/journal.pone.0263453)
Supplement: S4 File — (DOCX) [file pone.0263453.s004.docx]

HOSPITAL ASSEMBLY OF

MANDAQUI - CHM

**OPINION OF THE CEP**

**RESEARCH PROJECT DATA**

**Search Title:**TREATMENT OF BASOCELLULAR NEvuS SYNDROME WITH MULTIPLE SURGERIES - CASE REPORT AND LITERATURE REVIEW

**Researcher:** JEFFERSON ANDRE PIRES

**Subject Area:**

**Version:** 1

**CAAE:** 36967820.1.0000.5551

**Proposing Institution:** SAO PAULO SECRETARY OF HEALTH

**Main Sponsor:** Own financing

**OPINION DATA**

**Opinion Number:** 4,281,616

**Project presentation:**

Basal cell nevus syndrome or Gorlin syndrome is a rare genetic pathology. Its diagnosis is clinical and can present several manifestations. The treatment of this pathology must be multidisciplinary if there are clinical manifestations in addition to multiple basal cell carcinomas (BCC's), and in the presence of these only, surgical excision or treatments for skin cancer, as already recommended. The aim of this article is to report a case of treatment with multiple sequential surgeries in a patient with a clinical diagnosis of Gorlin syndrome, with the only manifestation of BCC's. The recommended treatment has been effective and safe, with patient satisfaction from an oncological, aesthetic and functional point of view.

**Research Objective:**

The objective of this work is to carry out a case report of a presentation of the basal cell nevus syndrome without other clinical manifestations besides multiple basal cell carcinomas, as well as to report the surgical treatment performed on the patient and to carry out a literature review.

**Risk and Benefit Assessment:**

Risks: Possibility of patient discomfort or embarrassment due to exposure of clinical and surgical history in scientific events.

Benefits: This case report may indirectly contribute to the patient or group of individuals who are in the same situation, as well as help other professionals of the

**Address:** PATRIA VOLUNTEERS 4301 - Building 4 - Blue House

| **Neighborhood:** SANTANA | | **ZIP CODE:** 02.401-400 | |
| --- | --- | --- | --- |
| **State:** SP | **County:** | SAO PAULO |  |
| **Telephone:** | (19) 2281-5147 | **Fax:** (19) 2281-5179 | **Email:** cepchm@gmail.com |

Page 01 of 03


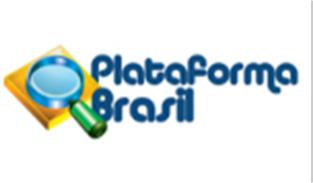
HOSPITAL ASSEMBLY OF

MANDAQUI - CHM

Continuation of Opinion: 4,281,616

plastic surgery as to the treatment performed.

**Research Comments and Considerations:**

Rationale: Basal cell nevus syndrome also called basal cell nevoid carcinoma syndrome or Gorlin syndrome or Gorlin-Goltz syndrome is a rare condition, first described in 1864 and later better described in 1960 by Gorlin and Goltz. This pathology has an autosomal dominant genetic etiology caused by mutations that inactivate the PATCH 1 tumor suppressor gene on chromosome 9q22.3 or, infrequently, mutations in the SUFU protein on chromosome 10q24.32, non-pathogenic variations are also described. The prevalence of this syndrome is estimated at 1:31,000-164,000 people. The diagnosis is clinical and can present several manifestations in addition to multiple basal cell carcinomas, such as odontogenic tumors, musculoskeletal, neurological, endocrine and ophthalmological alterations.

Material and methods are described in the project, with details of the entire process and data analysis. There will be use of secondary data sources (medical records) and there will be no retention of samples for storage in a bank.

**Considerations for Mandatory Submission Terms:**

All terms considered mandatory were presented.

**Recommendations:**

Communicate any and all changes to the project and informed consent form.

**Conclusions or Pending Issues and List of Inadequacies:**

All mandatory terms were presented, and after analyzing the project presented, I conclude by APPROVAL OF THIS RESEARCH PROJECT.

**Final Considerations at the discretion of the CEP:**

The Board fully accepted the opinion issued by the reporting member.

**This opinion was prepared based on the documents listed below:**

|  | Document Type | |  | Archive |  | Post |  | Author | Situation | |
| --- | --- | --- | --- | --- | --- | --- | --- | --- | --- | --- |
|  |  | |  |  | |  |  |  |  |  |
|  | Information | |  | PB_BASIC_INFORMATIONS_DO_P | | 08/13/2020 |  |  | Accepted | |
|  |  | |  | | |  |  |  |  |  |
|  |  | | | | |  |  |  |  |  |
|  | **Address:** PATRIA VOLUNTEERS 4301 - Building 4 - Blue House | | | | |  |  |  |  |  |
|  | **Neighborhood:** SANTANA | | | **ZIP CODE:** 02.401-400 | |  |  |  |  |  |
|  | **State:** SP | **County:** SAO PAULO | | |  |  |  |  |  |  |
|  | **Telephone:** | (19) 2281-5147 | | **Fax:** (19) 2281-5179 | **Email:** | cepchm@gmail.com | |  |  |  |
|  |  |  |  |  |  |  |  |  |  |  |

Page 02 of 03


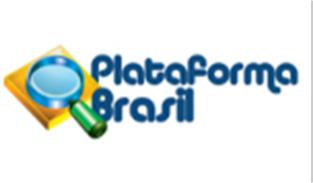
HOSPITAL ASSEMBLY OF

MANDAQUI - CHM

Continuation of Opinion: 4,281,616

| Project Basics | ETO_1611226.pdf | 15:53:08 |  | Accepted |
| --- | --- | --- | --- | --- |
|  |  |  |  |  |
| cover sheet | Cover Sheet.pdf | 08/13/2020 | JEFFERSON | Accepted |
|  |  | 15:52:11 | ANDRE PIRES |  |
| TCLE / Terms of | TERM_OF_CONFIDENTIALITY.do | 08/13/2020 | JEFFERSON | Accepted |
| Assent / | cx | 12:19:48 | ANDRE PIRES |  |
| Justification of |  |  |  |  |
| Absence |  |  |  |  |
| TCLE / Terms of | TERM_DE_AUTORIZACAO_PARA_U | 08/13/2020 | JEFFERSON | Accepted |
| Assent / | SO_DE_IMAGEM_IN_PUBLICATION_ | 12:19:38 | ANDRE PIRES |  |
| Justification of | SCIENTIFIC.docx |  |  |  |
| Absence |  |  |  |  |
| TCLE / Terms of | TCLE.docx | 08/13/2020 | JEFFERSON | Accepted |
| Assent / |  | 12:19:23 | ANDRE PIRES |  |
| Justification of |  |  |  |  |
| Absence |  |  |  |  |
| Detailed project / | gorlin_syndrome_project.docx | 08/13/2020 | JEFFERSON | Accepted |
| Brochure |  | 12:19:09 | ANDRE PIRES |  |
| Investigator |  |  |  |  |

**Status of Opinion:**

Approved

**Needs Assessment from CONEP:**

No

SAO PAULO, September 16, 2020

**Signed by:**

**Sergio Makabe**

**(Coordinator)**

**Address:** PATRIA VOLUNTEERS 4301 - Building 4 - Blue House

| **Neighborhood:** SANTANA | | **ZIP CODE:** 02.401-400 | |
| --- | --- | --- | --- |
| **State:** SP | **County:** | SAO PAULO |  |
| **Telephone:** | (19) 2281-5147 | **Fax:** (19) 2281-5179 | **Email:** cepchm@gmail.com |

Page 03 of 03
