# Supplementary material for: Effect of the combination of photobiomodulation therapy and the intralesional administration of corticoid in the preoperative and postoperative periods of keloid surgery: A randomized, controlled, double-blind trial protocol study
Source: PLoS One. 2022 Feb 15;17(2):e0263453. doi: 10.1371/journal.pone.0263453 (PMC8846523; doi:10.1371/journal.pone.0263453)
Supplement: S5 File — (DOCX) [file pone.0263453.s005.docx]

UNIVERSIDADE NOVE DE JULHO - UNINOVE

**PARECER CONSUBSTANCIADO DO CEP**

**Elaborado pela Instituição Coparticipante**

**DADOS DO PROJETO DE PESQUISA**

**Título da Pesquisa:**ESTUDO DO EFEITO DA ASSOCIAÇÃO DA TERAPIA DE FOTOBIOMODULAÇÃO E

1. APLICAÇÃO INTRALESIONAL DE CORTICOIDE NO PRÉ E PÓS-OPERATÓRIO

DA EXÉRESE DE QUELOIDES: UM ESTUDO CONTROLADO, RANDOMIZADO E DUPLO-CEGO

**Pesquisador:** JEFFERSON ANDRE PIRES

**Área Temática:**

**Versão:** 2

**CAAE:** 42419420.3.3001.5511

**Instituição Proponente:** ASSOCIACAO EDUCACIONAL NOVE DE JULHO

**Patrocinador Principal:** Financiamento Próprio

**DADOS DO PARECER**

**Número do Parecer:** 4.594.799

**Apresentação do Projeto:**

As informações elencadas nos campos "Apresentação do Projeto", "Objetivo da Pesquisa" e "Avaliação dos Riscos e Benefícios" foram retiradas do arquivo Informações Básicas da Pesquisa ("PB _ INFORMAÇÕES _ BÁSICAS _ DO _ PROJETO _ 1702723 . pdf" de 24/02/2021) .

As cicatrizes do tipo queloide são caracterizadas pela proliferação excessiva de fibroblastos e pela quebra do balanço entre a produção e degradação do colágeno, com seu aumento na derme. A gênese dessa patologia ainda não está totalmente elucidada, além dos aspectos genéticos também se sabe a que ela está relacionada ao aumento da expressão de TGF-. Ainda não existe um tratamento padrão ouro definido, e a recidiva está presente em todos preconizados. O tratamento mais estudado é a aplicação intralesional de corticoide sozinha ou associado no pré e pós- operatório da retirada do queloide. Devido a isso, novas alternativas de tratamento devem ser buscadas. A fotobiomodulação (FBM) com a luz azul tem demonstrado em estudos in vitro diminuição da velocidade de multiplicação e da quantidade de fibroblastos bem como do TGF- . É uma terapia de baixo custo, não invasivo e sem efeitos colaterais, mostrando-se uma boa ferramenta para associar ao tratamento mais preconizado. Dessa maneira o objetivo deste estudo é avaliar o efeito da luz azul associado ao tratamento com corticoide no pré e pós-operatório da

| **Endereço:** | VERGUEIRO nº 235/249 | |
| --- | --- | --- |
| **Bairro:** LIBERDADE | | **CEP:** 01.504-001 |
| **UF:** SP | **Município:** | SAO PAULO |
| **Telefone:** | (11)3385-9010 | **E-mail:** comitedeetica@uninove.br |
|  |  |  |

Página 01 de 06


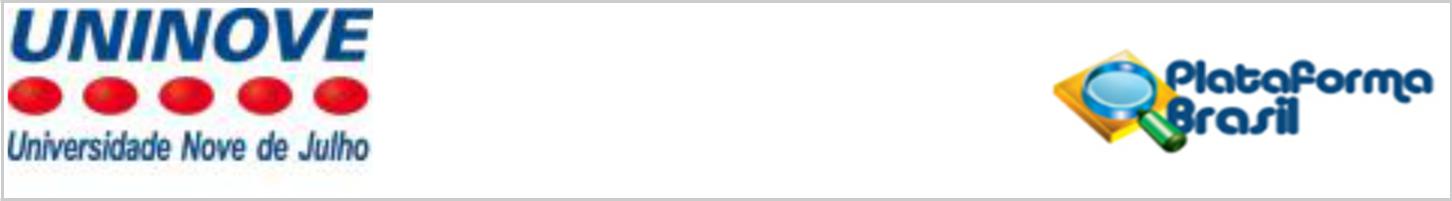
UNIVERSIDADE NOVE DE JULHO - UNINOVE

Continuação do Parecer: 4.594.799

exérese de queloides. Será realizado um ensaio clínico randomizado, controlado e duplo cego divididos em dois grupos: 1) Sham (N=29): aplicação intralesional de corticoide (AIC) no pré e pós-operatório da exérese de queloides e 2) FBM associado à AIC (N=29) no pré e pós-operatório da exérese dos queloides. A FBM será realizada de forma pontual transcutânea sobre o queloide no pré-operatório e na cicatriz remanescente no pós operatório utilizando caneta de LED azul (470nm, 400mW, 6,6mJ por ponto, sendo 10 pontos lineares). Os pacientes responderão a dois questionários, um para avaliação da qualidade de vida (Qualifibro-UNIFESP), e um para avaliação da satisfação da cicatriz (PSAQ) e a equipe de cirurgiões plásticos preencherão o questionário de Vancouver para avaliação da cicatriz (VSS), todos serão respondidos com 01, 03, 06 e 12 meses. Os queloides serão moldados no início do tratamento com silicone e antes da ressecção para avaliar o tamanho da área pré e pós-tratamento e da mesma maneira a cicatriz remanescente em 01, 03, 06 e 12 meses do pós-operatório. O queloide retirado será enviado para análises histopatológicas incluindo a quantidade de fibroblastos e a organização e distribuição de colágeno (coloração de picrossirius), e TGF-. Todos os dados serão submetidos a análise estatística.

**Objetivo da Pesquisa:**

Objetivo Primário:

Verificar os efeitos da combinação da FBM e da aplicação de corticoide intralesional sobre a taxa de recidiva de queloides após ressecção cirúrgica e sobre a qualidade da cicatriz neoformada.

Objetivo Secundário: avaliar os efeitos da FBM aplicada previamente a ressecção sobre a quantidade e organização de fibroblastos e colágenos, sobre a expressão gênica de TGF- e sobre a qualidade de vida dos participantes.

**Avaliação dos Riscos e Benefícios:**

Riscos:

Os riscos e desconfortos que o participante poderá ter são relacionados ao procedimento cirúrgico e

podem incluir manchas roxas no local da cirurgia e no local onde seja necessário aplicar alguma medicação;

desconforto na aplicação do anestésico local; sangramentos após a cirurgia, abertura da cicatriz antes

mesmo de retirar os pontos; infecções no local operado; reaparecimento do queloide e alterações na

coloração da pele.

Benefícios:

Como benefício direto o paciente realizará a retirada da cicatriz do tipo queloide e todo o

| **Endereço:** | VERGUEIRO nº 235/249 | |
| --- | --- | --- |
| **Bairro:** LIBERDADE | | **CEP:** 01.504-001 |
| **UF:** SP | **Município:** | SAO PAULO |
| **Telefone:** | (11)3385-9010 | **E-mail:** comitedeetica@uninove.br |
|  |  |  |

Página 02 de 06


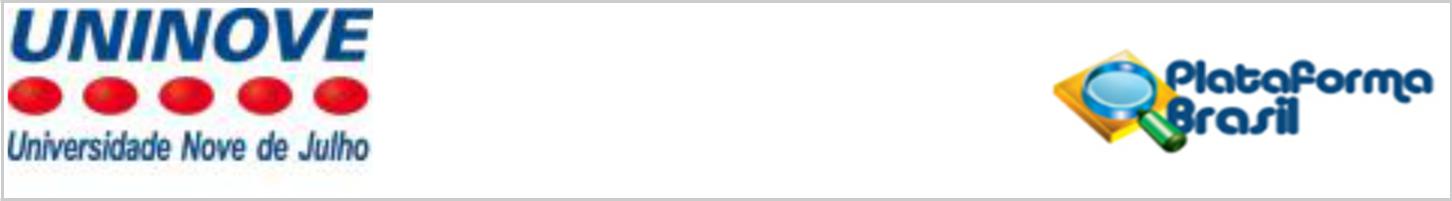
UNIVERSIDADE NOVE DE JULHO - UNINOVE

Continuação do Parecer: 4.594.799

acompanhamento e tratamento.

**Comentários e Considerações sobre a Pesquisa:**

Versão 2 do projeto.

Trata-se de um projeto de pesquisa já aprovado pelo CEP do Conjunto Hospitalar do Mandaqui (CAAE: 42419420.3.0000.5551) em que a UNINOVE foi incluída como instituição co-participante. Em breves palavras, é um ensaio clínico randomizado controlado e duplo cego em que 58 sujeitos, de ambos os sexos, com idade entre 18 e 65 anos, serão divididos em dois grupos: (1) Sham (n=29): aplicação intralesional de corticoide (AIC) no pré e pós-operatório da exérese de queloides + Luz placebo e (2) FBM associado à AIC (N=29) no pré e pós-operatório da exérese dos queloides. A fotobiomodulação será realizada semanalmente no primeiro mês de pós-operatório, a cada 15 dias no segundo mês de pós-operatório e uma aplicação no 3o mês de pós-operatório. Os pacientes com queloide serão recrutados no ambulatório do Serviço de Cirurgia Plástica do Conjunto Hospitalar do Mandaqui, na cidade de São Paulo-SP. Os pacientes responderão a dois questionários, um para avaliação da qualidade de vida (Qualifibro-UNIFESP), e um para avaliação da satisfação da cicatriz (PSAQ) e a equipe de cirurgiões plásticos preencherão o questionário de Vancouver para avaliação da cicatriz (VSS). Serão realizadas fotografias digitais da cicatriz do tipo queloide nas seguintes incidências: anterior; posterior, superior e laterais esquerda e direita com intuito de dar uma visão global da cicatriz. Os queloides serão moldados no início do tratamento com silicone e antes da ressecção para avaliar o tamanho da área pré e pós-tratamento e da mesma maneira a cicatriz remanescente. Todas essas avaliações serão realizadas com com 01, 03, 06 e 12 meses do pós-operatório. O queloide retirado será enviado para análises histopatológicas incluindo a quantidade de fibroblastos e a organização e distribuição de colágeno (coloração de picrossirius), e TGF-.

**Considerações sobre os Termos de apresentação obrigatória:**

- Folha de rosto: adequada (datada, assinada pelo diretor com carimbo do diretor – A instituição proponente é São Paulo Secretaria da Saúde e está assinada pelo Coordenador da Residência Médica do Hospital Mandaqui).
- Projeto de pesquisa - Na parte da metodologia, a UNINOVE não está incluída como local de coleta e consta do Termo de Confidencialidade que "também haverá dados coletados nos laboratórios da Universidade Nove de Julho". PENDÊNCIA ATENDIDA.

| **Endereço:** | VERGUEIRO nº 235/249 | |
| --- | --- | --- |
| **Bairro:** LIBERDADE | | **CEP:** 01.504-001 |
| **UF:** SP | **Município:** | SAO PAULO |
| **Telefone:** | (11)3385-9010 | **E-mail:** comitedeetica@uninove.br |
|  |  |  |

Página 03 de 06


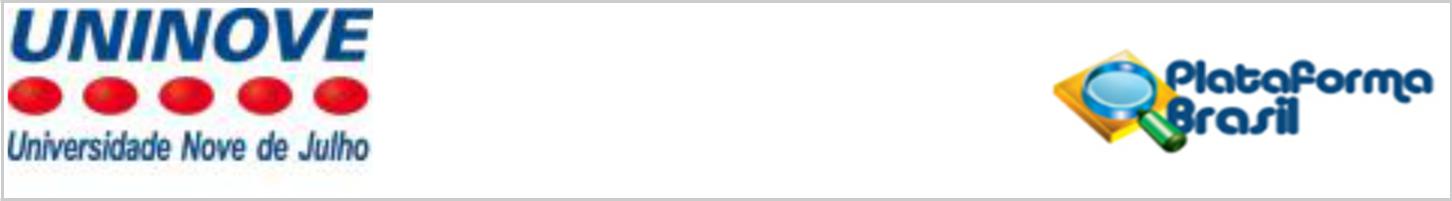
UNIVERSIDADE NOVE DE JULHO - UNINOVE

Continuação do Parecer: 4.594.799

- Cronograma – consta com data de início de recrutamento dos participantes em 01/02/2021. Já foi solicitado pelo parecer do CEP do Conjunto Hospitalar do Mandaqui que retificasse o cronograma (item Recomendações). PENDÊNCIA ATENDIDA.
- TCLE – Não consta do item 4 as seguintes informações:

1. que os participantes terão que responder os questionários de qualidade de vida (Qualifibro) e de satisfação da cicratiz (PSAQ) e que serão repetidos com 3, 6 e 12 meses; PENDÊNCIA ATENDIDA.
2. que os quelóides serão moldados no início do tratamento com silicone e antes da ressecção para avaliar o tamanho da área pré e pós-tratamento e da mesma maneira a cicatriz remanescente. PENDÊNCIA ATENDIDA.
3. que o quelóide retirado será enviado para biópsia. PENDÊNCIA ATENDIDA.
4. que as amostras do quelóide serão descartadas após análise. PENDÊNCIA ATENDIDA.

- Termo de confidencialidade - Adequado

Carta de anuência da instituição coparticipante – consta e está assinada pela Diretora do Programa de Pós-graduação em Biofotônica Aplicada às Ciências da Saúde da nossa universidade (UNINOVE).

**Recomendações:**

Não há recomendações.

**Conclusões ou Pendências e Lista de Inadequações:**

Projeto aprovado. Todas as pendências anteriores foram sanadas.

**Considerações Finais a critério do CEP:**

O pesquisador deverá se apresentar na instituição de realização da pesquisa (que autorizou a realização do estudo) para início da coleta dos dados.

O participante da pesquisa (ou seu representante) e o pesquisador responsável deverão rubricar todas as folhas do Termo de Consentimento Livre e Esclarecido - TCLE apondo sua assinatura na

| **Endereço:** | VERGUEIRO nº 235/249 | |
| --- | --- | --- |
| **Bairro:** LIBERDADE | | **CEP:** 01.504-001 |
| **UF:** SP | **Município:** | SAO PAULO |
| **Telefone:** | (11)3385-9010 | **E-mail:** comitedeetica@uninove.br |
|  |  |  |

Página 04 de 06


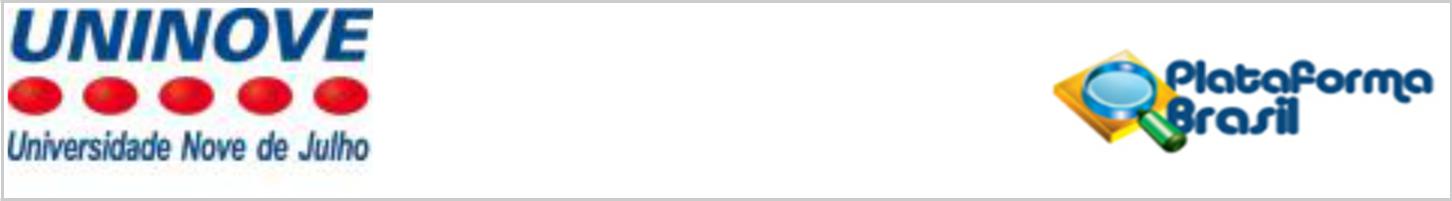
UNIVERSIDADE NOVE DE JULHO - UNINOVE

Continuação do Parecer: 4.594.799

última página do referido Termo, conforme Carta Circular no 003/2011 da CONEP/CNS.

Salientamos que o pesquisador deve desenvolver a pesquisa conforme delineada no protocolo aprovado.

Eventuais modificações ou emendas ao protocolo devem ser apresentadas ao CEP de forma clara e sucinta, identificando a parte do protocolo a ser modificada e suas justificativas. Lembramos que esta modificação necessitará de aprovação ética do CEP antes de ser implementada. De forma objetiva com justificativa para nova apreciação, os documentos alterados devem ser evidenciados para facilitar a nova análise.

Ao pesquisador cabe manter em arquivo, sob sua guarda, por 5 anos, os dados da pesquisa, contendo fichas individuais e todos os demais documentos recomendados pelo CEP (Res. CNS 466/12 item X1. 2. f).

De acordo com a Res. CNS 466/12, X.3.b), o pesquisador deve apresentar a este CEP/SMS os relatórios semestrais. O relatório final deverá ser enviado através da Plataforma Brasil, ícone Notificação. Uma cópia digital do projeto finalizado deverá ser enviada à instância que autorizou a realização do estudo, via correio, e-mail ou entregue pessoalmente, logo que o mesmo estiver concluído.

**Este parecer foi elaborado baseado nos documentos abaixo relacionados:**

|  | Tipo Documento | |  | Arquivo | Postagem | Autor | Situação | |
| --- | --- | --- | --- | --- | --- | --- | --- | --- |
|  |  | |  |  |  |  |  |  |
|  | Informações Básicas | |  | PB_INFORMAÇÕES_BÁSICAS_DO_P | 24/02/2021 |  | Aceito | |
|  | do Projeto | |  | ROJETO_1702723.pdf | 14:47:33 |  |  |  |
|  | TCLE / Termos de | |  | TCLE_doutorado_final.pdf | 24/02/2021 | JEFFERSON | Aceito | |
|  | Assentimento / | |  |  | 14:46:50 | ANDRE PIRES |  |  |
|  | Justificativa de | |  |  |  |  |  |  |
|  | Ausência |  |  |  |  |  |  |  |
|  | Projeto Detalhado / | |  | projeto_completo_corrigido.docx | 24/02/2021 | JEFFERSON | Aceito | |
|  | Brochura |  |  |  | 14:46:21 | ANDRE PIRES |  |  |
|  | Investigador | |  |  |  |  |  |  |
|  | Outros |  |  | CARTA_DE_ANUENCIA.pdf | 21/12/2020 | JEFFERSON | Aceito | |
|  |  |  |  |  | 15:34:45 | ANDRE PIRES |  |  |
|  | TCLE / Termos de | |  | CONFIDENCIALIDADE.pdf | 21/12/2020 | JEFFERSON | Aceito | |
|  | Assentimento / | |  |  | 15:34:00 | ANDRE PIRES |  |  |
|  |  |  | | |  |  |  |  |
|  | **Endereço:** | VERGUEIRO nº 235/249 | | |  |  |  |  |
|  | **Bairro:** LIBERDADE | | | **CEP:** 01.504-001 |  |  |  |  |
|  | **UF:** SP | **Município:** SAO PAULO | | |  |  |  |  |
|  | **Telefone:** | (11)3385-9010 | | **E-mail:** | comitedeetica@uninove.br | |  |  |
|  |  |  |  |  |  |  |  |  |

Página 05 de 06


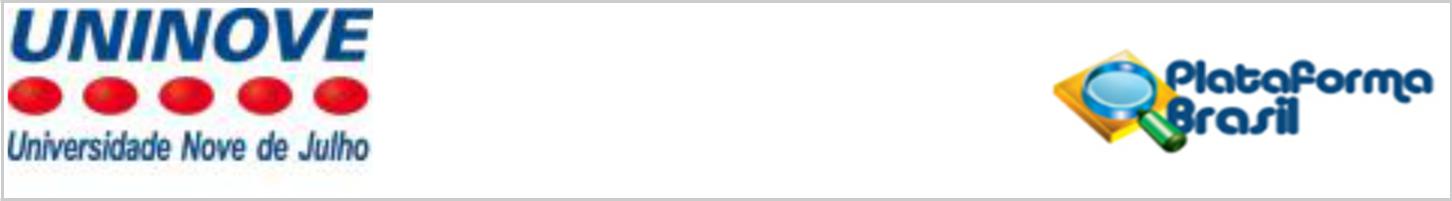
UNIVERSIDADE NOVE DE JULHO - UNINOVE

Continuação do Parecer: 4.594.799

| Justificativa de | CONFIDENCIALIDADE.pdf | 21/12/2020 | JEFFERSON | Aceito |
| --- | --- | --- | --- | --- |
| Ausência |  | 15:34:00 | ANDRE PIRES |  |
| TCLE / Termos de | TCLE_doutorado.pdf | 21/12/2020 | JEFFERSON | Aceito |
| Assentimento / |  | 15:33:31 | ANDRE PIRES |  |
| Justificativa de |  |  |  |  |
| Ausência |  |  |  |  |
| Projeto Detalhado / | projeto_completo.docx | 21/12/2020 | JEFFERSON | Aceito |
| Brochura |  | 15:32:33 | ANDRE PIRES |  |
| Investigador |  |  |  |  |

**Situação do Parecer:**

Aprovado

**Necessita Apreciação da CONEP:**

Não

SAO PAULO, 16 de Março de 2021

**Assinado por:**

**MARILIA DE ALMEIDA CORREIA**

**(Coordenador(a))**

| **Endereço:** | VERGUEIRO nº 235/249 | |
| --- | --- | --- |
| **Bairro:** LIBERDADE | | **CEP:** 01.504-001 |
| **UF:** SP | **Município:** | SAO PAULO |
| **Telefone:** | (11)3385-9010 | **E-mail:** comitedeetica@uninove.br |
|  |  |  |

Página 06 de 06
