# Supplementary material for: Effect of the combination of photobiomodulation therapy and the intralesional administration of corticoid in the preoperative and postoperative periods of keloid surgery: A randomized, controlled, double-blind trial protocol study
Source: PLoS One. 2022 Feb 15;17(2):e0263453. doi: 10.1371/journal.pone.0263453 (PMC8846523; doi:10.1371/journal.pone.0263453)
Supplement: S6 File — (DOCX) [file pone.0263453.s006.docx]

NINE OF JULY UNIVERSITY - UNINOVE

**OPINION OF THE CEP**

**Prepared by the Co-participating Institution**

**RESEARCH PROJECT DATA**

**Search Title:**STUDY OF THE EFFECT OF THE ASSOCIATION OF PHOTOBIOMODULATION AND

1. PRE- AND POST-OPERATIVE INTRALESIONAL CORTICOID APPLICATION

KELOID EXERESIS: A CONTROLLED, RANDOMIZED AND DOUBLE-BLIND STUDY

**Researcher:** JEFFERSON ANDRE PIRES

**Subject Area:**

**Version:** two

**CAAE:** 42419420.3.3001.5511

**Proposing Institution:** NINE DE JULY EDUCATIONAL ASSOCIATION

**Main Sponsor:** Own financing

**OPINION DATA**

**Opinion Number:** 4,594,799

**Project presentation:**

The information listed in the fields "Project Presentation", "Research Objective" and "Assessment of Risks and Benefits" were taken from the Basic Research Information file ("PB _ BASIC INFORMATION _ ON _ PROJECT _ 1702723 .pdf" of 24 02/2021) .

Keloid-type scars are characterized by the excessive proliferation of fibroblasts and the break in the balance between collagen production and degradation, with its increase in the dermis. The genesis of this pathology is not fully elucidated, in addition to the genetic aspects, it is also known that it is related to the increased expression of TGF-. There is still no defined gold standard treatment, and relapse is present in all recommended. The most studied treatment is the intralesional application of corticosteroids alone or in association in the pre- and postoperative period of keloid removal. Because of this, new treatment alternatives must be sought. Photobiomodulation (FBM) with blue light has been shown in in vitro studies to decrease the multiplication rate and the amount of fibroblasts as well as TGF- . It is a low-cost, non-invasive and without side effects therapy, proving to be a good tool to associate with the most recommended treatment. Thus, the aim of this study is to evaluate the effect of blue light associated with corticosteroid treatment in the pre- and postoperative period.

| **Address:** | VERGUEIRO No. 235/249 | |
| --- | --- | --- |
| **Neighborhood:** FREEDOM | | **ZIP CODE:** 01.504-001 |
| **State:** SP | **County:** | SAO PAULO |
| **Telephone:** | (11)3385-9010 | **Email:** comitedeetica@uninove.br |
|  |  |  |

Page 01 of 06


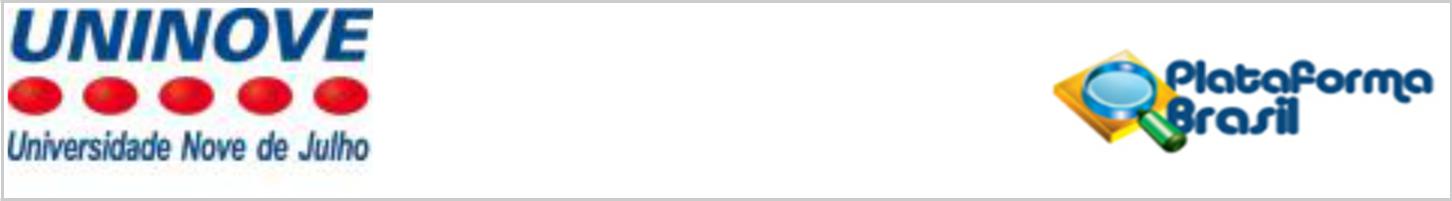
NINE OF JULY UNIVERSITY - UNINOVE

Continuation of Opinion: 4,594,799

exeresis of keloids. A randomized, controlled and double-blind clinical trial will be carried out, divided into two groups: 1) Sham (N=29): intralesional application of corticosteroids (AIC) in the pre- and postoperative period of keloid excision and 2) FBM associated with AIC ( N=29) in the pre- and postoperative period of keloid exeresis. FBM will be performed in a punctual transcutaneous way on the keloid in the preoperative period and on the scar remaining in the postoperative period using a blue LED pen (470nm, 400mW, 6.6mJ per point, with 10 linear points). Patients will answer two questionnaires, one to assess quality of life (Qualifibro-UNIFESP), and one to assess scar satisfaction (PSAQ) and the team of plastic surgeons will complete the Vancouver questionnaire for scar assessment (VSS), all will be answered with 01, 03, 06 and 12 months. Keloids will be molded at the beginning of the silicone treatment and before resection to assess the size of the pre- and post-treatment area and similarly the remaining scar at 01, 03, 06 and 12 months after surgery. The removed keloid will be sent for histopathological analysis including the amount of fibroblasts and the organization and distribution of collagen (picrosirius staining), and TGF-. All data will be subjected to statistical analysis.

**Research Objective:**

Primary Objective:

To verify the effects of the combination of FBM and the application of intralesional corticosteroids on the rate of keloid recurrence after surgical resection and on the quality of the newly formed scar.

Secondary Objective: to evaluate the effects of FBM applied prior to resection on the quantity and organization of fibroblasts and collagens, on the gene expression of TGF- and on the quality of life of the participants.

**Risk and Benefit Assessment:**

Scratchs:

The risks and discomforts that the participant may have are related to the surgical procedure and

may include bruises at the surgery site and where medication is needed;

discomfort in the application of the local anesthetic; bleeding after surgery, opening the scar before

even removing the stitches; infections at the operated site; reappearance of keloid and changes in

skin coloration.

Benefits:

As a direct benefit, the patient will remove the keloid scar and the entire

| **Address:** | VERGUEIRO No. 235/249 | |
| --- | --- | --- |
| **Neighborhood:** FREEDOM | | **ZIP CODE:** 01.504-001 |
| **State:** SP | **County:** | SAO PAULO |
| **Telephone:** | (11)3385-9010 | **Email:** comitedeetica@uninove.br |
|  |  |  |

Page 02 of 06


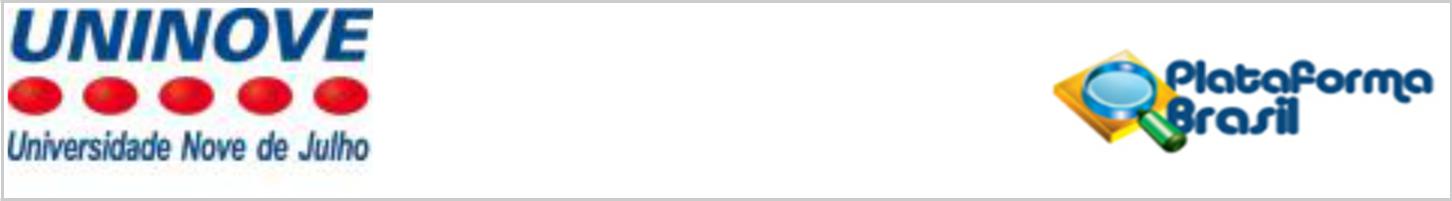
NINE OF JULY UNIVERSITY - UNINOVE

Continuation of Opinion: 4,594,799

monitoring and treatment.

**Research Comments and Considerations:**

Version 2 of the project.

This is a research project already approved by the CEP of the Conjunto Hospitalar do Mandaqui (CAAE: 42419420.3.0000.5551) in which UNINOVE was included as a co-participating institution. In brief, it is a randomized, double-blind, controlled clinical trial in which 58 subjects, of both sexes, aged between 18 and 65 years, will be divided into two groups: (1) Sham (n=29): intralesional application of corticosteroids (AIC) in the pre- and postoperative period of keloid exeresis + placebo light and (2) FBM associated with AIC (N=29) in the pre- and postoperative period of keloid exeresis. Photobiomodulation will be performed weekly in the first month after surgery, every 15 days in the second month after surgery and one application in the 3rd month after surgery. Patients with keloids will be recruited at the Plastic Surgery Service of the Conjunto Hospitalar do Mandaqui, in the city of São Paulo-SP. Patients will answer two questionnaires, one for assessment of quality of life (Qualifibro-UNIFESP), and one for assessment of scar satisfaction (PSAQ) and the team of plastic surgeons will complete the Vancouver questionnaire for scar assessment (VSS). Digital photographs of the keloid scar will be taken in the following views: anterior; posterior, superior and left and right sides in order to give a global view of the scar. Keloids will be molded at the beginning of the silicone treatment and before resection to assess the size of the pre- and post-treatment area and similarly the remaining scar. All these evaluations will be carried out with 01, 03, 06 and 12 months after surgery. The removed keloid will be sent for histopathological analysis including the amount of fibroblasts and the organization and distribution of collagen (picrosirius staining), and TGF-.

**Considerations for Mandatory Submission Terms:**

- Cover sheet: adequate (dated, signed by the director with the director's stamp – The proposing institution is São Paulo Health Secretariat and is signed by the Coordinator of the Medical Residency of Hospital Mandaqui).
- Research project - In the methodology part, UNINOVE is not included as a collection site and the Confidentiality Agreement states that "data will also be collected in the laboratories of Universidade Nove de Julho". PENDING ATTENDED.

| **Address:** | VERGUEIRO No. 235/249 | |
| --- | --- | --- |
| **Neighborhood:** FREEDOM | | **ZIP CODE:** 01.504-001 |
| **State:** SP | **County:** | SAO PAULO |
| **Telephone:** | (11)3385-9010 | **Email:** comitedeetica@uninove.br |
|  |  |  |

Page 03 of 06


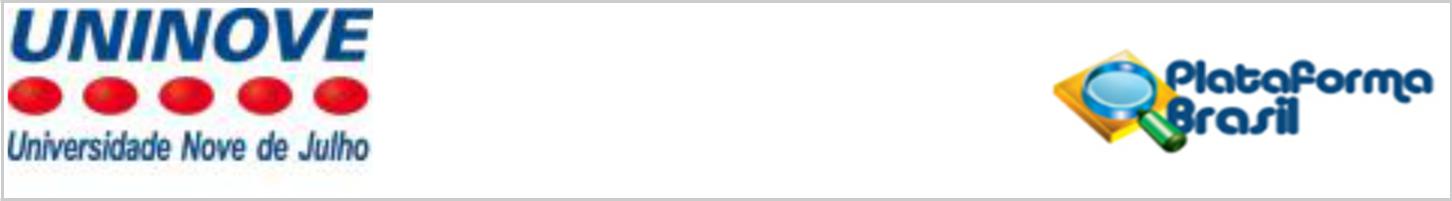
NINE OF JULY UNIVERSITY - UNINOVE

Continuation of Opinion: 4,594,799

- Schedule - includes the starting date of recruitment of participants on 02/01/2021. It has already been requested by the opinion of the CEP of Conjunto Hospitalar do Mandaqui to rectify the schedule (Recommendations item). PENDING ATTENDED.
- TCLE - Item 4 does not contain the following information:

1. that the participants will have to answer the quality of life (Qualifibro) and cicratiz satisfaction (PSAQ) questionnaires, which will be repeated at 3, 6 and 12 months; PENDING ATTENDED.
2. that the keloids will be molded at the beginning of the silicone treatment and before the resection to assess the size of the pre- and post-treatment area and likewise the remaining scar. PENDING ATTENDED.
3. that the removed keloid will be sent for biopsy. PENDING ATTENDED.
4. that the keloid samples will be discarded after analysis. PENDING ATTENDED.

- Confidentiality Agreement - Appropriate

Letter of agreement from the co-participating institution – it appears and is signed by the Director of the Graduate Program in Biophotonics Applied to Health Sciences at our university (UNINOVE).

**Recommendations:**

There are no recommendations.

**Conclusions or Pending Issues and List of Inadequacies:**

Approved project. All previous issues have been resolved.

**Final Considerations at the discretion of the CEP:**

The researcher must report to the institution where the research was carried out (which authorized the study to be carried out) to begin data collection.

The research participant (or their representative) and the responsible researcher must initial all the sheets of the Free and Informed Consent Term - TCLE affixing their signature on the

| **Address:** | VERGUEIRO No. 235/249 | |
| --- | --- | --- |
| **Neighborhood:** FREEDOM | | **ZIP CODE:** 01.504-001 |
| **State:** SP | **County:** | SAO PAULO |
| **Telephone:** | (11)3385-9010 | **Email:** comitedeetica@uninove.br |
|  |  |  |

Page 04 of 06


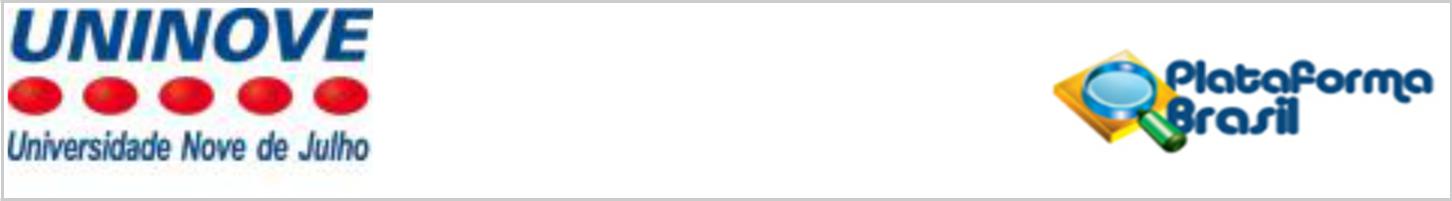
NINE OF JULY UNIVERSITY - UNINOVE

Continuation of Opinion: 4,594,799

last page of said Term, according to Circular Letter No. 003/2011 of CONEP/CNS.

We emphasize that the researcher must carry out the research as outlined in the approved protocol.

Any modifications or amendments to the protocol must be presented to the CEP in a clear and succinct manner, identifying the part of the protocol to be modified and its justifications. We remind you that this modification will require CEP ethical approval before being implemented. Objectively, with justification for a new assessment, the changed documents must be evidenced to facilitate the new analysis.

The researcher is responsible for keeping the research data on file, under his/her custody, for 5 years, containing individual files and all other documents recommended by the CEP (Res. CNS 466/12 item X1. 2. f).

In accordance with Res. CNS 466/12, X.3.b), the researcher must submit semiannual reports to this CEP/SMS. The final report must be sent through Plataforma Brasil, Notification icon. A digital copy of the completed project must be sent to the body that authorized the study, via mail, e-mail or delivered in person, as soon as it is completed.

**This opinion was prepared based on the documents listed below:**

|  | Document Type | |  | Archive | Post | Author | Situation | |
| --- | --- | --- | --- | --- | --- | --- | --- | --- |
|  |  | |  |  |  |  |  |  |
|  | Basic information | |  | PB_BASIC_INFORMATIONS_DO_P | 02/24/2021 |  | Accepted | |
|  | from the project | |  | ROJETO_1702723.pdf | 14:47:33 |  |  |  |
|  | TCLE / Terms of | |  | TCLE_doctorado_final.pdf | 02/24/2021 | JEFFERSON | Accepted | |
|  | Assent / | |  |  | 14:46:50 | ANDRE PIRES |  |  |
|  | Justification of | |  |  |  |  |  |  |
|  | Absence |  |  |  |  |  |  |  |
|  | Detailed project / | |  | project_complete_corrected.docx | 02/24/2021 | JEFFERSON | Accepted | |
|  | Brochure |  |  |  | 14:46:21 | ANDRE PIRES |  |  |
|  | Investigator | |  |  |  |  |  |  |
|  | Others |  |  | CARTA_DE_ANUENCIA.pdf | 12/21/2020 | JEFFERSON | Accepted | |
|  |  |  |  |  | 15:34:45 | ANDRE PIRES |  |  |
|  | TCLE / Terms of | |  | CONFIDENTIALITY.pdf | 12/21/2020 | JEFFERSON | Accepted | |
|  | Assent / | |  |  | 15:34:00 | ANDRE PIRES |  |  |
|  |  |  | | |  |  |  |  |
|  | **Address:** | VERGUEIRO No. 235/249 | | |  |  |  |  |
|  | **Neighborhood:** FREEDOM | | | **ZIP CODE:** 01.504-001 |  |  |  |  |
|  | **State:** SP | **County:** SAO PAULO | | |  |  |  |  |
|  | **Telephone:** | (11)3385-9010 | | **Email:** | comitedeetica@uninove.br | |  |  |
|  |  |  |  |  |  |  |  |  |

Page 05 of 06


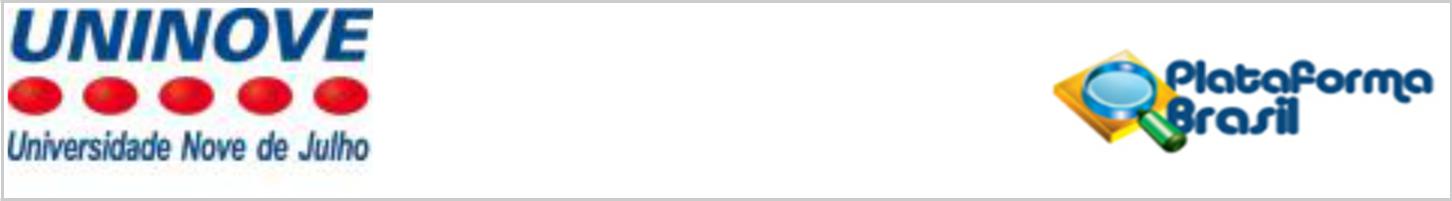
NINE OF JULY UNIVERSITY - UNINOVE

Continuation of Opinion: 4,594,799

| Justification of | CONFIDENTIALITY.pdf | 12/21/2020 | JEFFERSON | Accepted |
| --- | --- | --- | --- | --- |
| Absence |  | 15:34:00 | ANDRE PIRES |  |
| TCLE / Terms of | TCLE_doctorate.pdf | 12/21/2020 | JEFFERSON | Accepted |
| Assent / |  | 15:33:31 | ANDRE PIRES |  |
| Justification of |  |  |  |  |
| Absence |  |  |  |  |
| Detailed project / | complete_project.docx | 12/21/2020 | JEFFERSON | Accepted |
| Brochure |  | 15:32:33 | ANDRE PIRES |  |
| Investigator |  |  |  |  |

**Status of Opinion:**

Approved

**Needs Assessment from CONEP:**

No

SAO PAULO, March 16, 2021

**Signed by:**

**MARILIA DE ALMEIDA CORREIA**

**(Coordinator)**

| **Address:** | VERGUEIRO No. 235/249 | |
| --- | --- | --- |
| **Neighborhood:** FREEDOM | | **ZIP CODE:** 01.504-001 |
| **State:** SP | **County:** | SAO PAULO |
| **Telephone:** | (11)3385-9010 | **Email:** comitedeetica@uninove.br |
|  |  |  |

Page 06 of 06
